# A Subcutaneous Implant of Tenofovir Alafenamide Fumarate Causes Local Inflammation and Tissue Necrosis in Rabbits and Macaques

**DOI:** 10.1128/AAC.01893-19

**Published:** 2020-02-21

**Authors:** Jonathan T. Su, Solange M. Simpson, Samuel Sung, Ewa Bryndza Tfaily, Ronald Veazey, Mark Marzinke, Jiang Qiu, David Watrous, Lakmini Widanapathirana, Elizabeth Pearson, M. Melissa Peet, Dipu Karunakaran, Brooke Grasperge, Georgina Dobek, Charlette M. Cain, Thomas Hope, Patrick F. Kiser

**Affiliations:** a Department of Biomedical Engineering, Northwestern University, Evanston, Illinois, USA; b Department of Physics and Engineering, Elon University, Elon, North Carolina, USA; c Department of Cell and Molecular Biology, Northwestern University, Chicago, Illinois, USA; d Division of Comparative Pathology, Tulane National Primate Research Center, Covington, Louisiana, USA; e Division of Clinical Pharmacology, Johns Hopkins University School of Medicine, Baltimore, Maryland, USA; f CONRAD, Contraceptive Research and Development, Department of Obstetrics and Gynecology, Eastern Virginia Medical School, Arlington, Virginia, USA; g Tulane University School of Medicine, New Orleans, Louisiana, USA; h Feinberg School of Medicine, Northwestern University, Chicago, Illinois, USA

**Keywords:** antiretroviral agents, histopathology, implant, preexposure prophylaxis

## Abstract

We describe the *in vitro* and *in vivo* evaluation of a subcutaneous reservoir implant delivering tenofovir alafenamide hemifumarate (TAF) for the prevention of HIV infection. These long-acting reservoir implants were able to deliver antiretroviral drug for over 90 days *in vitro* and *in vivo*. We evaluated the implants for implantation site histopathology and pharmacokinetics in plasma and tissues for up to 12 weeks in New Zealand White rabbit and rhesus macaque models. A dose-ranging study in rabbits demonstrated dose-dependent pharmacokinetics and local inflammation up to severe necrosis around the active implants. The matched placebos showed normal wound healing and fibrous tissue encapsulation of the implant. We designed a second implant with a lower release rate and flux of TAF and achieved a median cellular level of tenofovir diphosphate of 42 fmol per 10^6^ rhesus macaque peripheral blood mononuclear cells at a TAF dose of 10 μg/kg/day. This dose and flux of TAF also resulted in adverse local inflammation and necrosis near the implant in rhesus macaques. The level of inflammation in the primates was markedly lower in the placebo group than in the active-implant group. The histological inflammatory response to the TAF implant at 4 and 12 weeks in primates was graded as a severe reaction. Thus, while we were able to achieve a sustained target dose, we observed an unacceptable inflammatory response locally at the implant tissue interface.

## Abstract (precis)

We describe the *in vitro* and *in vivo* evaluation of a subcutaneous reservoir implant delivering tenofovir alafenamide hemifumarate (TAF) for the prevention of HIV infection. These long-acting reservoir implants were able to deliver antiretroviral drug for over 90 days *in vitro* and *in vivo*. We evaluated the implants for implantation site histopathology and pharmacokinetics in plasma and tissues for up to 12 weeks in New Zealand White rabbit and rhesus macaque models.

## INTRODUCTION

Clinical availability of antiretroviral (ARV) delivery systems that provide durable protection from HIV transmission could revolutionize the way that we fight the global HIV/AIDS pandemic (1). Once-daily oral Truvada (emtricitabine [FTC] at 200 mg and tenofovir disoproxil fumarate [TDF] at 300 mg) prevents the sexual transmission of HIV when used before sexual exposure to HIV (2–6). However, not all preexposure prophylaxis (PrEP) trials with oral Truvada have been efficacious (2–6), likely because of poor adherence to the regimen (7, 8). Therefore, many groups in the HIV prevention field are striving to develop long-acting, acceptable, and effective methods of HIV prevention for use in high-risk populations. For example, a recent clinical study demonstrated effective drug levels of a subdermal drug-eluting implant releasing islatravir (MK-8591) for the prevention of sexual transmission of HIV (9).

Long-acting drug delivery systems are fundamentally easier for individuals to use than once-daily oral pills. Studies on adherence to methods of contraception generally show that the increased duration and the subsequent reduced need for daily repeated action by the user are correlated with increased contraceptive efficacy (10–13). Subcutaneous contraceptive implants generate durable, sustained progestin exposure over several years and allow the recipient to undergo a minimally invasive procedure for implant placement without any further clinical follow-up until removal; therefore, contraceptive implants are the most effective contraceptives. Similarly, long-acting delivery systems for ARVs have the potential to be highly effective.

Long-acting drug delivery systems require the most potent and slowly eliminated ARVs to enable durable ARV exposure and protection for durations on the order of months to a year. Tenofovir alafenamide hemifumarate (TAF) (or GS-7340-03) is a caspase-activated prodrug of tenofovir (TFV) (14). Based on *in vitro* analysis, the 50% effective concentration (EC_50_) of TAF is in the low-nanomolar range (5 to 11.2 nM) (15, 16). TAF is intracellularly converted by kinases into TFV diphosphate (TFV-DP); this is the active form of the drug that competitively inhibits HIV reverse transcriptase and the generation of viral transcripts. The diphosphate, due to its charge and pK_a_, is highly impermeative to cellular membranes and is therefore trapped in the intracellular volume versus the parent species. TFV-DP cellular half-lives in humans have been measured at ∼150 h (17). For several other molecular reasons that have been reviewed previously (18, 19), TAF is a more potent prodrug than TDF, resulting in low TFV drug exposures and reduced side effects compared to TDF (14). Together, these characteristics make TAF one of the leading drug molecules for long-acting ARV delivery because TAF is so potent and cellularly long acting that one can plausibly load many ARV daily doses inside a small controlled-release device to achieve durable protection from HIV infection.

Accordingly, four subcutaneous implants delivering TAF exist in the literature. The first implant using the free base of TAF, presented by Gunawardana et al., delivered 0.92 mg/day (∼80 μg/kg/day) and was evaluated in beagle dogs for over 40 days (20). This implant consisted of 1.9-mm-diameter silicone tubing with 14 poly(vinyl alcohol)-coated 1.0-mm-diameter delivery channels punched into the walls and filled with pure TAF powder (20). The second implant was presented by Schlesinger et al. and consisted of a heat-sealed poly(caprolactone) film cylinder containing TAF and polyethylene glycol 300 at a 1:2, 1:1, or 2:1 (wt/wt) ratio (21). Release rates ranging from 0.5 to 4.4 mg/day were demonstrated *in vitro* (21). A third implant was presented by Johnson et al. and was a reservoir formed from extruded poly(caprolactone) filled with TAF and a castor oil excipient; release rates of 0.15 to 0.91 mg/day were demonstrated *in vitro* (22). The fourth implant, presented by Chua et al., consisted of a refillable titanium device that delivered TAF and FTC through silicon nanochannels (23). This refillable implant demonstrated a sustained release of TAF of ∼0.2 mg/day (∼20 μg/kg/day) for 83 days in rhesus macaques (23). They rapidly achieved TFV-DP benchmark levels in macaque peripheral blood mononuclear cells (PBMCs) with means of 72 fmol/10^6^ PBMCs early in the pharmacokinetic (PK) study to 533 fmol/10^6^ PBMCs to day 70. None of the studies have reported placebo-controlled histopathology, but all authors have suggested that the implants are safe (20, 23, 24).

When a subcutaneous implant is placed under the skin, the cells and tissues surrounding the implant respond to the presence of the foreign-body implant and potentially to the drug near the implantation site. In the normal foreign-body response, the implant is walled off at its site of implantation by a fibrin-containing capsule (25). Focal toxicity can result in inflammation and necrosis, potentially leading to skin disruption and potential infection (26). Ultimately, any process that disrupts the multistage wound-healing response can result in a nonbiocompatible drug delivery implant. It is also possible that these toxic effects are dependent on the route of administration, thus driving the need for careful evaluation of the biological and cellular responses at the implant-tissue interface.

We evaluated the potential viability of TAF for systemic, long-term drug delivery, using a subcutaneous implant made of a heat-sealed polyurethane rate-controlling membrane. Our objective was to develop a subcutaneous TAF reservoir implant that, after implantation, would show no signs of pathology at the implant site yet provide levels of TFV-DP that could prevent sexual transmission of HIV (26). In this work, we evaluated TAF implants in both New Zealand White (NZW) rabbit and rhesus macaque models. We describe the conducted studies below to assess the local biological reaction to active TAF implants versus matched placebo implants and TFV-DP pharmacokinetics and *in vivo* release rates in both NZW rabbits and rhesus macaques for up to 12 weeks.

## RESULTS

### Implant design and *in vitro* performance.

**FIG 1:**
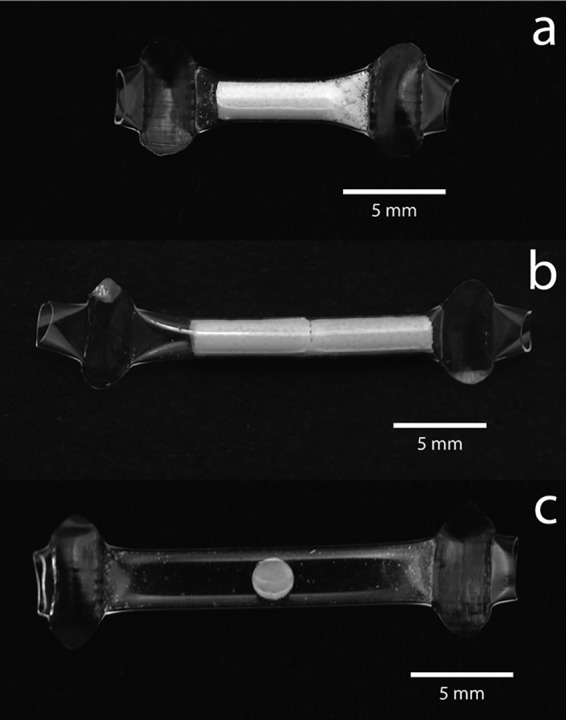
Photos of representative generation A TAF long-acting reservoir implants with lumen lengths of 0.8 cm (a) and 1.6 cm (b) and a placebo implant that is empty except for a pellet of NaCl and magnesium stearate (c).

TAF reservoir implants were formed by compressing the TAF drug substance and small amounts of NaCl and magnesium stearate into a pellet that was impulse sealed into a 150- to 170-μm thin, medical-grade polyurethane tube (Fig. 1). The tube wall acts as a mechanical capsule and rate-controlling membrane whose composition or thickness can be changed to tune the drug release rate. Accordingly, we were able to successfully control the release of TAF by changing the geometry of the implant as well as the composition of the polyurethane membrane. Drug release from implants was evaluated *in vitro* (see Fig. S1 in the supplemental material) using the shake flask method. We observed low rates of TAF degradation (27) in phosphate-buffered saline (PBS) in *in vitro* release testing medium (half-life of 2.8 days at pH 7.4 at 37°C in PBS) as well as in the implant (see Supplemental 1 and 2 in the supplemental material file), although until the end of the release curve, we observed that >90% of the internal contents were in the parent form. Molar amounts of TAF and its main TAF-related substances [(*R*)-9-(2-phosphonomethoxypropyl)adenine (PMPA) monoamidate and tenofovir] were calculated and converted to a mass of TAF depleted from the core. Two generations of implants are described in this study: generation A and generation B.

Two types of generation A implants were created: one with a 0.8-cm lumen length and one with a 1.6-cm lumen length. The average TAF equivalent *in vitro* release rates over days 7 to 91 from the generation A implant were 0.13 mg/day for the 0.8-cm-long implants and 0.26 mg/day for the 1.6-cm-long implants, with fluxes of 0.24 mg TAF/cm^2^/day and 0.23 mg TAF/cm^2^/day, respectively. We were able to obtain a sustained release of the drug *in vitro* (Fig. S1a and b) for over 100 days. Guided by our pharmacokinetics and histopathology from studies on the generation A implants, we developed a second-generation implant, called generation B, that was designed to release a smaller amount and flux of drug but also to provide a steady release of TAF *in vitro* (Fig. S1c). The mean TAF equivalent *in vitro* release rate over days 7 to 91 from the generation B implants was 0.13 mg/day, with an average flux of 0.08 mg TAF/cm^2^/day.

### Pharmacokinetic and local safety evaluation in rabbits.

Both generations of TAF implants were evaluated in animals. Four studies were performed: (i) a PK and safety dose-ranging study using generation A implants in NZW rabbits using four dose groups, (ii) a PK study using generation A implants in rhesus macaques, (iii) a PK and local-response study using generation B implants in rhesus macaques, and (iv) an exploratory study in rhesus macaques to assess the local reaction to generation B implants when inserted by trocar.

**FIG 2:**
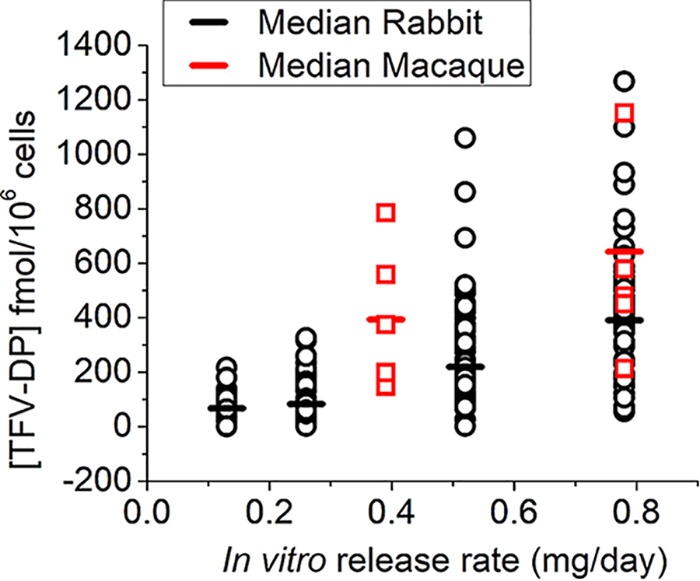
TFV-DP concentrations determined in NZW rabbits and macaques for generation A TAF implants. The median was determined from data points from weeks 1 to 12.

**FIG 3:**
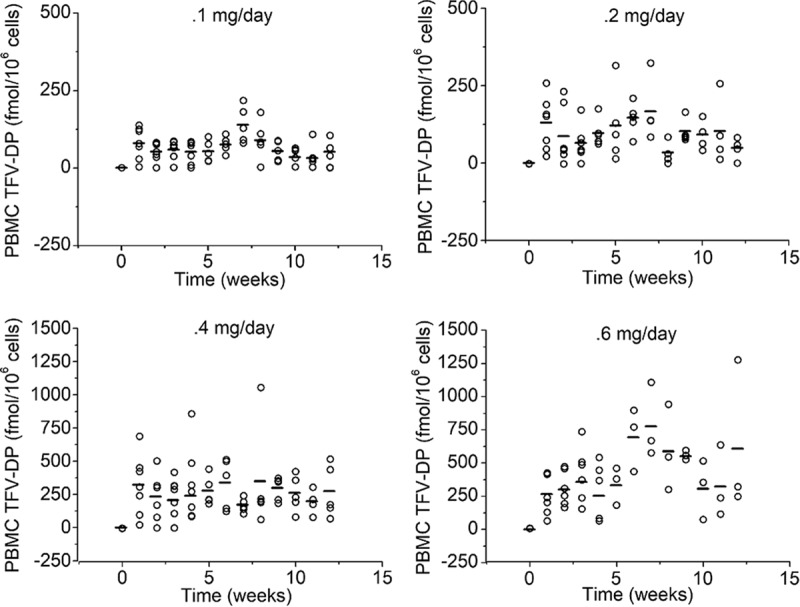
Dose-dependent PK observed in our NZW rabbit experiments, with higher average TFV-DP levels leading to higher plasma levels of TFV-DP in circulating heterophils. TFV-DP levels increased within a week of implantation. BLQ values are plotted as 1/10 of the calculated limit-of-quantification (LOQ) value. Drug levels for all placebo implants were BLQ. The horizontal bar is the mean TFV-DP concentration. As per Table 1, *in vitro* release from group 1 was 0.13 mg/day, that from group 2 was 0.26 mg/day, that from group 3 was 0.52 mg/day, and that from group 4 was 0.78 mg/day.

Table 1 summarizes the results from the first three animal studies in this series of analogous implants delivering TAF. A series of four doses was evaluated in NZW rabbits (Table 1). TFV-DP was found in blood heterophils for all active-implant-treated animals after week 1, and concentrations were quantifiable throughout the study (Fig. 2 and 3). In general, increases in the *in vitro* dose were correlated with increasing median TFV-DP concentrations. Group 1, receiving our lowest *in vitro* dose, 0.13 mg/day, corresponded to a median TFV-DP level of 68 fmol/10^6^ cells (range from below the level of quantification [BLQ] to 218 fmol/10^6^ cells); the median was calculated from cellular PK data from weeks 1 to 12. Group 4, receiving our highest *in vitro* dose of 0.78 mg/day, 6 times higher than the lowest dose, provided a median TFV-DP level of nearly 391 fmol/10^6^ cells over weeks 1 to 12 of the study. This implant gave a median cellular TFV-DP level 5.75 times higher than the lower dose.

**TABLE 1:** PK and pathohistological scores in NZW rabbits and rhesus macaques^*a*^

Implant	Group	Avg *in vitro* TAF flux^*b*^ (mg/cm^2^/day)	Avg *in vitro* release rate^*c*^ (mg/day)	Avg *in vivo* dose^*d*^ (μg/kg/day)	Mean cellular TFV-DP concn^*e*^ (fmol/10^6^ cells) ± SD	Median cellular TFV-DP concn^*e*^ (fmol/10^6^ cells) (range)	Mean total histological characteristic score ± SD^*f*^
Generation A, NZW rabbits	Placebo	NR	NR	NR	NR	NR	1.5 ± 0.6^*g*^
	1	0.24	0.13	18.5	66 ± 45	68 (BLQ–218)	17.5 ± 0.6^*g*^
	2	0.24	0.26	35.4	100 ± 75	84 (BLQ–326)	21.4 ± 4.8^*g*^
	3	0.23	0.52	69.4	277 ± 191	220 (BLQ–1,060)	23.0 ± 5.1^*g*^
	4	0.23	0.78	106.2	412 ± 271	391 (56–1,268)	21.3 ± 5.5^*g*^

Generation A, rhesus macaques		0.24	0.39	28.6	377 ± 289	394 (62–1,912)	NP
		0.23	0.78	56.3	431 ± 155	643 (38–4,769)	NP

Generation B, rhesus macaques		Placebo	NR	NR	NR	NR	6.5^*h*^
							11.3 ± 8.2^*g*^
		0.08	0.13	10^*i*^	60 ± 54	42 (BLQ–255)	19.5^*h*^
							24.7 ± 9.7^*g*^

a NP, not performed in this exploratory PK study; NR, not relevant.

b Calculated from geometry and *in vitro* release rates.

c Calculated from the average release rate (see Fig. S1a to c in the supplemental material) from days 7 to 91.

d Calculated from depleted TAF from the implant over the study duration and group average body mass.

e Cellular TFV-DP concentrations were averaged over all time points after time zero. NZW rabbits have heterophils, and rhesus macaques have PBMCs.

f Scores ranged from 0 to 32.

g Slides and scores obtained at 12 weeks.

h Slides and scores obtained at 4 weeks (*n* = 2). No standard deviation was calculated.

i Estimated value from *in vitro* release data in Fig. S1c from days 7 to 91.

Plasma TFV concentrations remained low for all NZW rabbits, with TFV concentrations ranging from BLQ to 20 ng/ml (lower limit of quantification [LLOQ] = 0.31 ng/ml [weeks 1 to 12]) (Supplemental 3 and Table S3). Similarly, drug and metabolite concentrations in vaginal and rectal tissue were generally low in NZW rabbits (Supplemental 3 and Table S2). Values of TFV-DP in vaginal tissues and rectal tissues ranged from BLQ to 169 fmol/mg and from BLQ to 50 fmol/mg (LLOQ = 50 fmol/sample [weeks 1 to 12]), respectively. Samples were taken near the implant site at necropsy, and the TFV-DP levels were scattered; high concentrations of TFV-DP were found at week 12. Local tissue TFV-DP concentrations ranged from 0.86 to 69,941 fmol/mg of TFV-DP.

**FIG 4:**
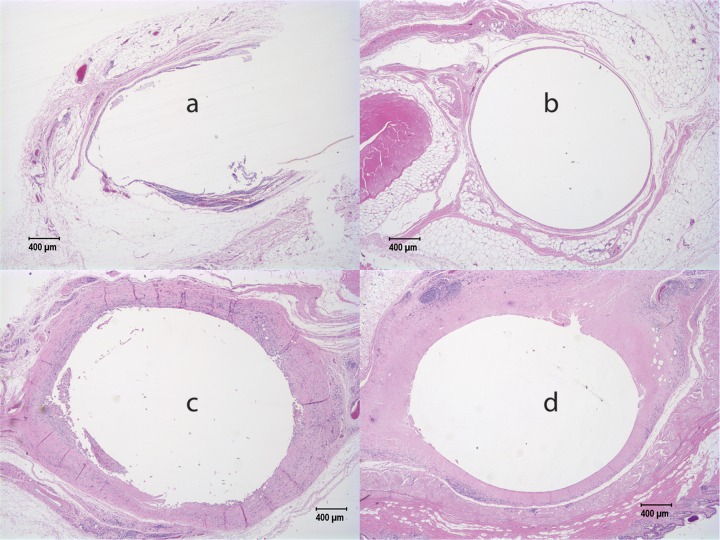
Contralateral sections from NZW rabbits after 12 weeks with the generation A implant. Sections from two animals (animals M29051 [a and c] and M29049 [b and d]) are shown. Both animals came from the lowest dose group 1. Minimal inflammation was observed for our placebo implants (a and b); however, extensive inflammation with some necrosis was observed for implants containing the active drug (c and d).

All NZW rabbits appeared healthy, with no superficial observations of poor tolerability at the implant site throughout the study. Figure 4 shows representative sections of histology from NZW rabbits with generation A implants after 12 weeks. In the placebo implants (Fig. 4a and b), the implant region was demarcated by a thin fibrous tissue capsule, 2 to 5 cells thick, but most of the sections showed no or mild inflammation in the area around the implant, with two implants showing mild to moderate inflammation. We saw that some rabbits appeared to have inflammation near the end of the implant adjacent to the sutures. The active implants shown in Fig. 4c and d displayed severe granulomatous and suppurative inflammation with necrosis and abundant necrotic cell debris and proteinaceous fluid in the implant space, which is lined by marked infiltrations of lymphocytes and heterophils. Marked infiltrations of lymphocytes and macrophages into the adjacent muscle tissues were seen. There was also abundant eosinophilic fluid-like material with pockets of necrotic cellular debris with associated granulomatous inflammation and giant cells. Some slides showed scattered yet diffuse infiltrations of lymphocytes, heterophils, and macrophages in the dermis and muscle fibers. Additionally, in some NZW rabbits, chronic granulomatous inflammation and necrosis were observed around the implant.

Histopathological characteristics were scored semiquantitatively on an animal and implant basis from multiple slides taken from the ends and the center of fixed tissue containing implants. The scoring system (0 to 4) logged the presence of five cellular characteristics (polymorphonuclear cells, lymphocytes, plasma cells, macrophages, and giant cells) and three tissue characteristics (necrosis, capsule thickness, and tissue infiltrate). High levels of inflammation were observed in the peri-implant space from all drug-loaded implants at all doses in NZW rabbits (ranging from an average total histological characteristic score ± standard deviation [SD] of 17.5 ± 0.6 to 23.0 ± 5.1, averaged over all time points from weeks 1 to 12) (Table 1). These total histological characteristic scores were far higher than those observed for the placebo implants, which had an average score ± SD of 1.5 ± 0.6 (averaged over all time points from weeks 1 to 12) (Table 1) (see Supplemental 4 for score tables and micrographs for all animals). There was no statistically significant difference (*P* > 0.05) in total histological characteristic scores with TAF-exposed NZW rabbit groups. The 0.8- and 1.6-cm generation A implants obtained a reactivity grade of severe reaction (see Materials and Methods and Supplemental 6 in the supplemental material).

After our *in vivo* experiments, implants were placed on *in vitro* release to verify that the implants were intact, were not leaking, and were releasing drug at the moment of removal (see Fig. 6). The release rate after explantation from the 1.6-cm-lumen-length implants was roughly double that of the 0.8-cm-lumen-length implants, as expected. Additionally, we extracted these implants for the calculation of an average *in vivo* release rate (Fig. S6). Comparison of our *in vivo* release rates with our *in vitro* release rates demonstrated correlations of close to 0.8 at 4 weeks and approximately 0.7 to 0.8 at 12 weeks (Fig. S6). All returned implants remained intact, and the wall was not compromised. We investigated if any molecular weight changes had occurred in the polymers during their residence *in vivo*. No notable changes in molecular weight distributions that would indicate *in vivo* polymer degradation were detected (Supplemental 7 and Table S16).

### Pharmacokinetic and local safety evaluation in rhesus macaques.

**FIG 5:**
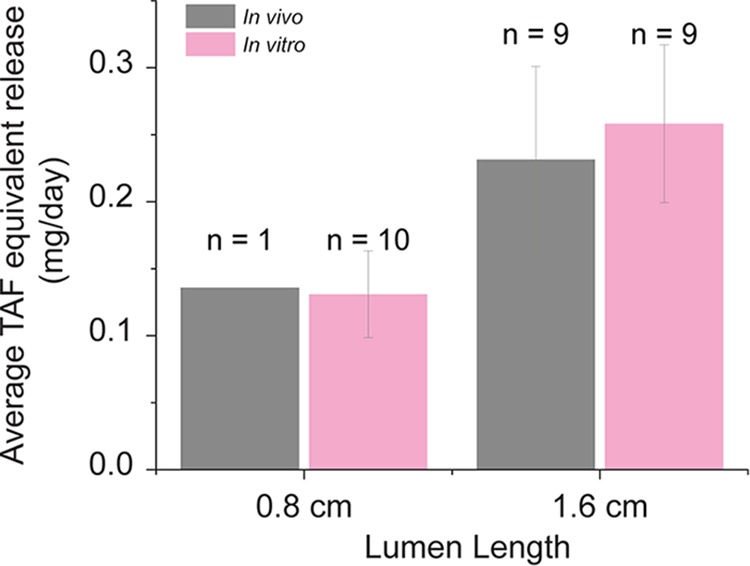
*In vitro* and *in vivo* comparison of average daily release rates from TAF generation A implants in rhesus macaques after 12 weeks. The *in vitro* release is the average over days 7 to 91 from representative implants in the same batch as the *in vivo* implants.

Following experiments with generation A implants in NZW rabbits, we conducted a dose-finding pharmacokinetic experiment in rhesus macaques with the same implant system (Table 1). In generation A-implanted macaques, there was no statistically significant difference found (*P* > 0.05) for TFV-DP levels for either our 0.39-mg/day *in vitro* dose or our 0.78-mg/day *in vitro* dose (Fig. 2). This study allowed us to design a lower-dose and -flux generation B implant to achieve lower levels of TFV-DP. For the low and high doses, one animal each lost an implant due to abscess formation. A comparison of our *in vivo* release rates with our *in vitro* release rates demonstrated a close correlation (Fig. 5).

**FIG 6:**
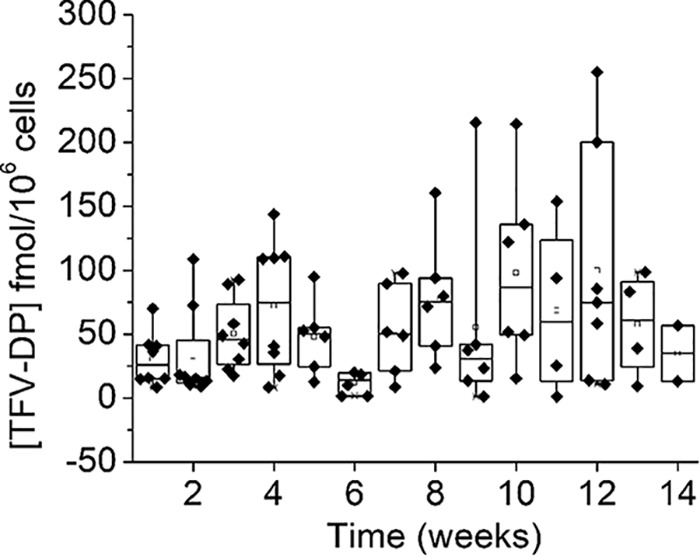
TFV-DP levels in macaque PBMCs from the generation B implants.

For generation B implants releasing an average of 0.13 mg TAF/day *in vitro*, the median TFV-DP concentrations in rhesus macaque PBMCs were 42 fmol/10^6^ cells (range, BLQ to 255 fmol/10^6^ cells), calculated from weeks 1 to 12 data (Fig. 6 and Table 1). Plasma TFV and TAF concentrations remained low for all rhesus macaques, with TFV concentrations ranging from BLQ to 7 ng/ml (LLOQ = 0.35 ng/ml) and TAF concentrations ranging from BLQ to 4 ng/ml (TAF LLOQ = 0.03 ng/ml) (Supplemental 8 and Table S19). Similarly, TFV and TFV-DP levels in tissue were generally low for all macaques, with full data provided in Supplemental 8 and Table S18. Samples were taken near the implant site and from the vagina and the rectum. All TFV concentrations near the implant site were BLQ (LLOQ = 0.05 ng/sample), and TFV-DP concentrations near the implant site were low, ranging from BLQ to 27 fmol/mg (LLOQ = 5 fmol/sample). Rectal concentrations were similarly low, with a TFV range from BLQ to 0.24 ng/mg; the TFV-DP level ranged from BLQ to 12 fmol/mg. In the vagina, concentrations were again low: the TFV level ranged from BLQ to 0.02 ng/mg, while the TFV-DP level ranged from BLQ to 6 fmol/mg.

**FIG 7:**
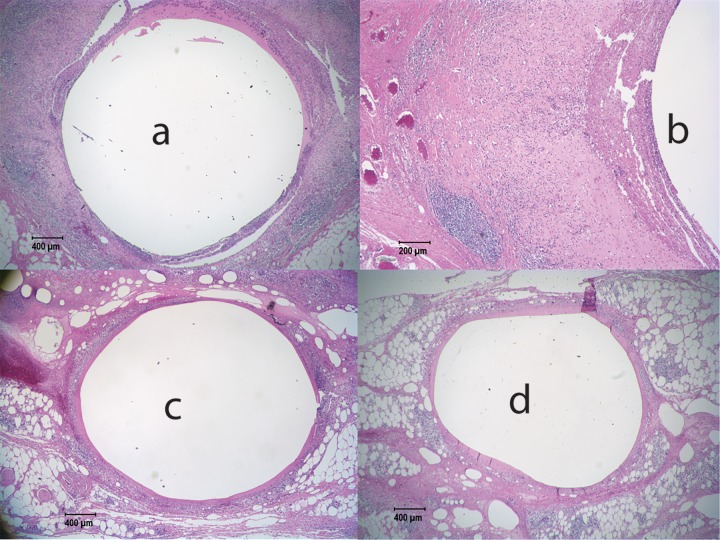
Representative histology slides from rhesus macaque FC48 after 12 weeks with a generation B implant. Minimal inflammation and a distinct fibrous tissue capsule were observed for the placebo implant (c and d); however, extensive inflammation was observed for the implant containing the active drug (a and b) despite a lower *in vitro* release rate for generation B implants (0.13-mg/day *in vitro* release rate) than for generation A implants.

Despite the lower flux of TAF release in the generation B implants, the level of inflammation remained high in all rhesus macaques exposed to the active implants. For the active generation B implants in macaques, we observed gross redness around the implant and neovascularization indicative of inflammation. All placebos showed visually clear encapsulation with no neovascularization and redness around the placebo implant. Representative pathohistology for the generation B implants in macaques is shown in Fig. 7, with the full set of images in Supplemental 5 and Fig. S27 to S32. Histologically, we observed moderate to severe inflammation in the peri-implant space, with thick fibrous capsules filled with neutrophils, plasma cells, necrotic cellular debris, proteinaceous fluid, and occasional multinucleated giant cells. Multifocal aggregates of densely packed lymphocytes were observed in surrounding tissues. In two of the four macaques, we observed an abscess above the implant site, where it appeared that the implant caused a topical wound. The two animals that were necropsied at 4 weeks also displayed markedly more inflammation than the animals with placebo contralateral implants, but the necrosis scores for the active-implant sites were mild at 4 weeks (Supplemental 5) and became moderate to severe by 12 weeks.

In contrast, two out of four placebo implants showed minimal fibrosis or inflammation, with a few neutrophils and plasma cells in the implant lumen, except in one case, where marked accumulations of macrophages and lymphocytes with moderate numbers of giant cells were observed around the placebo implant. In this animal (EC74) (Table S12), moderate inflammation was observed around the placebo implant adjacent to the sutures, and the active paired implant demonstrated a purulent hemorrhagic abscess with fibrosis resulting in the loss of the drug-loaded implant before necropsy. Thus, while the score of the placebo implant (20) was relatively higher than the score of the other placebo implants, it was still lower than the score of the corresponding active implant (28). Overall, high levels of inflammation were observed in the peri-implant space in rhesus macaques (ranging from an average total histological characteristic score ± SD of 24.7 ± 9.7 at week 12) (Table 1). These total histological characteristic scores were far higher than those observed for the placebo implants, which had an average score of 11.3 ± 8.2 at 12 weeks (see Supplemental 5 for score tables and micrographs for all animals in these studies). Finally, the computed reaction grade indicated a severe reaction to the reduced-dose and -flux generation B implant (Supplemental 6 and Table S15).

We sought to determine if the use of a trocar instead of surgical-pocket formation would modify the local inflammation. Here, two male and two female rhesus macaques were implanted with contralateral matched placebos and single generation B implants. We observed that subdermal wounds in several animals developed into abscesses and surface lesions (Supplemental 9 and Fig. S33). The use of a trocar allowed more efficient implant insertion with less trauma but did not reduce or eliminate the local reaction to the generation B implants.

## DISCUSSION

The dosing calculations for the design of the generation A and B TAF subcutaneous implants were targeted to cellular benchmark concentrations of TFV-DP in PBMCs, established in humans (20, 29, 30). Median PBMC TFV-DP concentrations in the STRAND trial were 42 fmol/10^6^ cells with once-daily oral dosing (29). Due to upwards of 66% TFV-DP losses during cryopreservation, using iPrEX analysis, Gunawardana et al. estimated a conservative EC_90_ of 24 to 48 fmol/10^6^ cells for PBMC TFV-DP concentrations (20). This TFV metabolite level was the benchmark applied in this work, as described below, for which drug exposure is too low to likely protect from HIV transmission.

Both generation A and generation B implant formulations demonstrated the controlled release of TAF with sustained concentrations of intracellular TFV-DP throughout implant exposure (12 weeks). We hypothesized that this inflammatory reaction might be TAF dose (milligrams per day) and TAF flux (milligrams per square centimeter per day) dependent. By reducing both, we hoped to attenuate the cellular inflammatory reactions described above while achieving protective levels of TFV-DP in PBMCs of primates. We also thought that there might be species-dependent toxicity in NZW rabbits that might not manifest itself in nonhuman primates. Thus, in our design of the generation B subcutaneous TAF implants, we reduced our *in vitro* release rate from 0.39 mg/day to 0.13 mg/day and reduced TAF flux from 0.24 to 0.08 mg TAF/cm^2^/day (Table 1). Assuming linear dose scaling from the generation A implant in rhesus macaques, we expected this implant to yield roughly 100 fmol/10^6^ cells of TFV-DP.

While we achieved our TFV-DP cellular benchmark PK levels in rhesus macaques, and the flux and dose were significantly reduced in the generation B implant, we continued to observe significant inflammation at 4 and 12 weeks in the peri-implant volume in rhesus macaques (Fig. 7; for full histology reports, see Supplemental 5 in the supplemental material). Levels of local inflammation and necrosis around the implant in all cases were much higher in the TAF implant arms than for the matched placebos, suggesting that inflammation is caused by TAF exposure to the local cells and tissue around the implant. This local inflammation occurred even with the lowest-TAF-dose and -flux implant that achieves commonly used cellular TFV-DP benchmark levels. Furthermore, the lower level of inflammation surrounding the placebo implants strongly suggests that the local histopathology is due to neither the polymer nor the surgical procedure of blunt dissection or trocar administration.

Chua et al. are the only group to report data on TAF implants in rhesus macaques, and they exceeded benchmark levels of TFV-DP in PBMCs. With a dose in rhesus macaques of 200 μg/day of TAF or approximately 20 μg/kg/day, they report a mean TFV-DP level of 533 fmol/10^6^ PBMCs (23). Gunawardana et al. delivered controlled doses of 920 μg/day in beagle dogs, with a corresponding dose of 85 μg/kg/day (20). We delivered a lower dose of 130 μg/day in rhesus macaques, or 10 μg/kg/day, and obtained lower median TFV-DP levels than Chua et al., yet we still observed severe histopathological reactions and inflammation around the active implants at a 10-μg/kg/day TAF dose and not with the placebos (Table 1).

Although we are the first to report extensive histological data on TAF implants, we are not the first to report on implant safety or tissue pathology near an active TAF implant. When Chua et al. evaluated their system in rhesus macaques (23), they reported the presence of wound formation along the surgical incision or “dehiscence” over their TAF implant in two out of the three macaques, in addition to skin ulceration over the implant in two animals at day 70 (23). Ultimately, Chua et al. concluded that histopathology of punch biopsy specimens of skin sampled adjacent to the implant was normal in rhesus macaques, but no control implant data were collected (23). It is not clear from Chua et al. if the whole implant was removed and fixed for the two reported tissue sections. Using a poly(caprolactone) implant similar to the one described by Schlesinger et al. (21), Gatto et al. reported on a “cutaneous response” to the devices in rabbits (24). In the study by Gunawardana et al., safety in beagle dogs was “evaluated by body weight and cage observations” (20). No histopathological analysis data were provided, but from animal weight and cage observations, they concluded that the implant was safe. However, in later unpublished pharmacokinetic studies of beagle dogs, this implant was associated with “erythema and/or edema at the implantation site” as well as “multiple instances of discharge” (31). Those authors then argued that these reactions would not be observed at doses of less than 1 mg/day of TAF (31); our findings belie this expectation, as we observe inflammation at doses well below 1 mg/day (∼100 μg/kg/day) in rhesus macaques.

TAF is safe when administered orally (14), and TFV has been safely administered vaginally, both as a gel (28, 32) and as an intravaginal ring (33). It is well known that the route of administration can significantly modify the toxicological response to the drug delivery system. For example, entecavir is an approved oral antiviral used for the treatment of chronic hepatitis B (34). However, Henry et al. evaluated polymer-coated pellets of entecavir in rats and observed local swelling, scab formation, and necrosis in their drug-loaded implants but not their placebos (35). Similarly, we have observed little inflammation around the placebo implants but observed significant inflammation around the drug-containing implants. Much like the studies by Anderson et al. and Henry et al., we removed the entire implantation site for histopathological analysis after exposure to the drug delivery system.

TAF undergoes pH-dependent hydrolysis into two main related substances, PMPA monoamidate and TFV (Table S1). Over 70 days, upwards of ∼90% of the mass flux from the generation B implant is TAF, with about half of PMPA amidate being generated over the 24 h that the compound is sitting in *in vitro* release testing medium. Furthermore, both of these compounds have been observed in the portal and jugular veins of dogs orally exposed to TAF (36). Our work has not eliminated the possibility that differences in the interior microenvironment of other implant compositions could lead to other implants releasing a mixture of TAF-related substances that differs from that of the implants described here. These different mixtures of TAF-related substances could result in modifications in the toxicokinetics of a TAF implant system. Because we have not studied this implant system side by side with the other reported TAF implants, it remains a possibility that other differently constructed TAF implants will not suffer the same local toxicity issues as the ones that we observed in our reservoirs.

### Conclusion.

We describe a reservoir implant capable of delivering TAF in the subcutaneous space for a period of several months, and we tested this implant system in NZW rabbits and rhesus macaques for up to 12 weeks. Our main finding in this work is that this TAF implant always induces local inflammation around the implant, even at a low dose of the drug (∼10 μg/kg/day). We felt that we could not reasonably decrease the dose or flux further and still generate a viable TAF implant that would protect from HIV transmission and be a reasonable size in humans. More importantly, our results could not exclude the possibility that this reservoir TAF implant, loaded with hundreds of milligrams of the drug, could leak from a manufacturing or mechanical failure and cause tissue damage to the user because of exposure to a large acutely applied dose of TAF in the subcutaneous volume. Together, these factors caused us to conclude that this implant is unsafe and to terminate preclinical development efforts toward this TAF implant for long-acting HIV prevention and treatment.

Our work was informed by guidance for studying local inflammation around an implanted device described by ISO 10993-6 for the biological evaluation of medical devices (37). ISO 10993-6 directs us to “excise the implant site together with sufficient unaffected surrounding tissue to enable evaluation of the local histopathological response” (37). Leaving the implant in the tissue allows histological evaluation of the pericapsular space around the implant without disturbing the fragile cellular and biopolymer structures that are used to measure the histopathological response versus the control. We followed this procedure, and our histological analysis clearly shows a local toxic response with the TAF-loaded implant and a considerable reduction of such a response in our matched placebos. Because of the locally high drug concentrations and long drug exposures in the local tissues around the implant (36), we urge that the international standard (37) is followed for all future ARV implant local-reaction studies. We also observed that the inflammation and necrosis around the implants became more severe at later time points, suggesting that a 1-month local safety study would be insufficient for these long-acting devices. Finally, we recommend extensive stress testing of TAF- and other ARV-eluting devices to exclude rupture, device failure, and dose dumping.

While the oral route of administration of TAF (14, 38) is clinically proven to be safe, the TAF implants described here are unsafe. Alternatively, another potent, long-acting, small-molecule ARV, like cabotegravir (39–41) or GS-6207 (42), could be progressed with studies like those shown in this work to achieve the goal of a long-acting subcutaneous implant for the treatment and prevention of HIV infection. 4′-Ethynyl-2-fluoro-2′-deoxyadenosine (EFdA) (islatravir; MK-8591) (39–41) is a highly potent nucleoside reverse transcriptase translocation inhibitor: once-weekly oral dosing has demonstrated its ability to protect male rhesus macaques in challenge studies (43). Islatravir implants have demonstrated prophylactic concentrations in a human trial (9, 44). We further suggest that long-acting ARV drug substances should be evaluated for biocompatibility at the site of administration earlier in the preclinical development process. For example, an Alzet osmotic pump delivering a set dose would permit the screening of local inflammation as a function of the dose in an animal without requiring the full design of a drug delivery device capable of long-acting durations. Thus, similar reactions can be studied and avoided in future devices using other ARV drugs.

## MATERIALS AND METHODS

### Materials.

TAF (CAS 1392275-56-7; GS-7340-03) and TFV (CAS 147127-20-6) were obtained from Gilead Sciences (Foster City, CA). Tecoflex polyurethane was obtained from Lubrizol (Wickliffe, OH). Tips, die, and the die head used for extrusion were sourced from Guill Tool (West Warwick, RI). The dies used to press pellets for TAF and placebo implants were purchased from Natoli (St Charles, MO). Sodium chloride and magnesium stearate (USP grade) used to manufacture implants were obtained from Spectrum Chemical (New Brunswick, NJ). Barium sulfate (USP grade) used for manufacturing radiopaque rods was obtained from Fisher Scientific (Fair Lawn, NJ). Sodium azide, ammonium acetate, a phosphate-buffered saline solution, and solvents used for high-performance liquid chromatography (HPLC) and mass spectrometry (MS) (32) work were obtained from Fisher Scientific (Fair Lawn, NJ). Isotopically labeled adenine-[U-^13^C]TFV (TFV*) was obtained from Moravek Biochemicals (Brea, CA). Syringe filter tips, weighing dishes, and centrifuge tubes were obtained from Fisher (New Hampton, NH). Scintillation vials for *in vitro* release were obtained from Wheaton (Rockford, TN).

### Animal care and welfare.

All animal studies were conducted in accordance with protocols approved by Northwestern University and Tulane National Primate Research Center Local Institutional Animal Care and Use Committees, Northwestern protocol IS00006125 and Tulane protocol P0307R. This study was carried out in accordance with the *Guide for the Care and Use of Laboratory Animals* of the Institute of Laboratory Resources, National Research Council (45). All procedures were performed under anesthesia using ketamine-xylazine, and all efforts were made to minimize stress, improve housing conditions, and provide enrichment opportunities. Animals were euthanized by sedation with ketamine-xylazine injection followed by intravenous barbiturate overdose in accordance with the recommendations of the panel on euthanasia of the American Veterinary Medical Association.

### Description of implant manufacturing.

Implant manufacturing was completed in a nonsterile environment. However, all drug product-contacting surfaces, including the benchtop surfaces, machines, and floors, were cleaned with a 3% hydrogen peroxide solution and ethanol. All materials used in manufacturing were depyrogenated by heating glass and stainless steel materials to 250°C or rinsing heat-incompatible materials with a 3% hydrogen peroxide solution. During manufacturing, all staff wore face masks, hairnets, disposable gowns, gloves, and shoe covers to minimize contamination. Further description of implant manufacturing is provided in the supplemental material. The implants were shipped to Steri-tek Inc. (Fremont, CA) for electron beam sterilization with a radiation dose of 25 kGy. Implants received back after sterilization were tested for endotoxin levels.

### Endotoxin testing.

Endotoxin levels of all raw materials and pre-/post-e-beam-sterilized implants were quantified by chromogenic detection of lipopolysaccharide (LPS) using the method provided with the Pierce *Limulus* amebocyte lysate chromogenic endotoxin quantitation kit. The FDA predefines endotoxin units (EU) (3) as 20.0 EU/device, which is approximately 0.5 EU/ml from a 40-ml rinse volume. All materials and implants used in the studies had levels below the assay LLOQ of 0.15 EU/ml. All materials used in this assay were purchased endotoxin or pyrogen free. Samples were first fully submerged in 3 ml of Ficoll-Plaque Plus endotoxin-free water (GE Healthcare, Uppsala, Sweden) in either a 6-well culture plate or a 3-ml test tube for 1 h at 37°C at 800 rpm on a Multitherm benchmark (Benchmark, Edison, NJ). Samples were analyzed (*n* = 3) alongside a positive-control spiked sample to a concentration of 0.8 EU/ml on a 96-well, polystyrene-bottom plate at a 405- to 410-nm wavelength.

### Initial selection of dose in NZW rabbits.

To start our dose-ranging studies, we allometrically scaled the lowest dose in our rabbit PK study to roughly the maximum estimate by Gunawardana et al. (20) for efficacious TAF exposure in humans; this turns out to be 0.1 mg/day in a 3.2-kg rabbit. The other rabbit doses that we chose were scaled in multiples from this dose and are 0.2 mg/day, 0.4 mg/day, and 0.6 mg/day (Table 1). This would provide a range of doses starting at their maximum estimate of a TAF dose, twice that, and up to six times that in rabbits. Allometrically, in rabbits, our highest dose exceeds the dose that Gunawardana et al. tested in beagle dogs. This range should allow us to develop PK dose-response and toxicokinetic curves and ranges over the doses that should be required for TAF. We manufactured implant systems that achieve the TAF doses shown in Table 2, and these are described below.

**TABLE 2:** Generation A implant systems used in the NZW rabbit PK and safety study

Animal group	*In vitro* release rate^*a*^ (mg/day)	Material	Pellet diam (mm)	Implant diam (mm)	Implant lumen length (cm)	Membrane thickness (cm)	Mean implant strength of TAF (mg) ± SD	No. of active implants/animal
1	0.13	Tecoflex EG-85A	1.8	2.2	0.8	0.015	16.8 ± 0.3	1
2	0.26	Tecoflex EG-85A	1.8	2.2	0.8	0.015	16.8 ± 0.3	2
3	0.52	Tecoflex EG-85A	1.8	2.2	1.6	0.015	34.0 ± 0.3	2
4	0.78	Tecoflex EG-85A	1.8	2.2	1.6	0.015	34.0 ± 0.3	3
5	Placebo	Tecoflex EG-85A	1.8	2.2	1.6	0.015	NR	1 or 2

a Calculated from the average release rate (Fig. S1) once the implants reached steady state over days 7 to 91.

### TAF implant formulation characteristics.

We evaluated two generations of TAF implants: generation A and generation B. Generation A implants were evaluated in NZW rabbits for PK and safety and in rhesus macaques for PK. Characteristics and dimensions of the generation A implants are shown in Tables 2 and 3. Based on results from generation A implants, generation B implants with lower doses were manufactured and evaluated in rhesus macaques. Dimensions and characteristics for generation B implants are given in Table 4.

**TABLE 3:** Generation A formulation characteristics and description of manufacturing used for macaques

Animal group	*In vitro* release rate^*a*^ (mg/day)	Material	Pellet diam (mm)	Implant diam (mm)	Membrane thickness (cm)	Implant lumen length (cm)	Mean implant strength of TAF (mg) ± SD	No. of active implants/animal
1	0.39	Tecoflex EG-85A	1.8	2.2	0.015	0.8	16.8 ± 0.3	1
						1.6	34.0 ± 0.3	1
2	0.78	Tecoflex EG-85A	1.8	2.2	0.015	1.6	34.0 ± 0.3	3

a Calculated from the average release rate (Fig. S1) once the implants reached a pseudo-steady state over days 7 to 91.

**TABLE 4:** Generation B formulation characteristics

*In vitro* release rate^*a*^ (mg/day)	Materials (ratio)	Pellet diam (mm)	Implant diam (mm)	Membrane thickness (mm)	Implant lumen length (cm)	Mean implant strength of TAF (mg) ± SD
0.13	Tecoflex EG-85A–EG-93A (50:50)	2.0	2.6	0.17	2.0	44.4 ± 1.7
Placebo	Tecoflex EG-85A–EG-93A (50:50)	2.0	2.6	0.17	2.0	NR

a Calculated from the average release rate (Fig. S1) once the implants reached steady state over days 7 to 91.

### Animal study design, group sizes, and controls.

Overall, as mentioned above, we conducted four animal PK studies. The first two sets of studies used the generation A TAF implant in both NZW rabbits and rhesus macaques. The third study used the findings from the first studies to justify a dose reduction and evaluate a generation B TAF implant in rhesus macaques. The fourth study used a trocar to implant the devices. The sampling schedule for all studies remained the same (Table 5).

**TABLE 5:** Design of PK and safety studies^*a*^

Procedure(s)	Schedule at time point (wk)												
	0	1	2	3	4	5	6	7	8	9	10	11	12
Implant	X												
Plasma TFV, TAF	X	X	X	X	X	X	X	X	X	X	X	X	X
PBMC TFV-DP	X	X	X	X	X	X	X	X	X	X	X	X	X
Vaginal swab	X		X		X		X		X		X		X
Rectal swab	X		X		X		X		X		X		X
Vaginal biopsy	X				X				X				X
Rectal biopsy	X				X				X				X
Necropsy^*b*^					X								X

a PK and safety studies followed the same schedule for TAF generation A in NZW rabbits and TAF generation B in macaques. Necropsy of the animals was performed at weeks 4 and 12. All necropsy was accompanied by histology and staining. For generation A implant studies with rhesus macaques, only a PK study was performed, with no necropsy at the end of the study.

b Necropsy of the rhesus macaques was not performed in the generation A nonhuman primate implant study, and therefore, this was a PK study only.

The rabbit study had five groups of female NZW rabbits. In the placebo group, there were three animals. In group 1, seven animals each received a 0.8-cm active implant and a contralateral placebo. In groups 2 and 3, seven animals each received two 0.8-cm or two 1.6-cm active implants, respectively. In group 4, six animals received three 1.6-cm implants. Group 5 acted as the control group and consisted of three animals implanted with two placebos each. Two of seven animals in groups 1, 2, and 3 and three of six in group 4 were sacrificed during week 4. Five of seven animals in groups 1, 2, and 3; three of six in group 4; and all three placebo controls were sacrificed during week 12. The average body mass of the rabbits was 3.37 ± 0.21 kg, with a range of 2.84 to 3.81 kg.

The macaque generation A exploratory PK study included two groups, each of which contained three females. The first group received one 0.8-cm implant and one 1.6-cm implant. The second group received three 1.6-cm implants. Animals were maintained on study for 12 weeks, and the mean mass of the animals was 6.5 ± 0.3 kg, with a range of 6.2 to 7.0 kg. The macaque generation B study had four groups of animals with two animals each. Each animal received two implants, one placebo and one active implant (both 2 cm), contralaterally. Group 1 (one male and one female) was necropsied at 4 weeks, and group 2 (two females) was necropsied at 12 weeks. Blood, tissue, and PBMCs were still sampled per the overall schedule. All animals had at least one active implant. The rhesus macaques had a mean body mass of 12.7 ± 4.4 kg, with a range of 7 to 19.6 kg.

The use of contralateral implantation of implants provided us several opportunities to simultaneously study *in vivo* drug release rates of the implants and local irritation. Contralateral implantation of an active implant and a placebo implant was used in the generation A rabbit studies (group 1) and the generation B rhesus macaque studies (all macaques). In animals with two or more contralateral active implants, one implant was resected in a block of tissue with the intact implant, fixed, and sectioned to evaluate local inflammation around the implant histologically. The other implant was removed and tested for *in vitro* performance. These implants were subjected to *in vitro* release and leak testing, and the polymer molecular weight distribution was determined (see Supplemental 7 in the supplemental material). For each implant, we tracked its initial mass, the strength of the pellet, and the total pellet mass; this allowed us to determine the total drug content after explantation and obtain the average daily release rate of the devices over the 3-month study by subtraction.

Finally, we conducted a fourth exploratory PK and safety study in rhesus macaques to evaluate if the insertion of the generation B device with a trocar would modify the biological response to the implant. Here, there were two groups of two males and two females implanted contralaterally with an active implant and a matched placebo using a 4.5-mm trocar kit. Other than the method of insertion and the number of animals, the study design was identical. The macaques had an average mass of 14.24 ± 3.6 kg and a mass range of 9.4 to 18.0 kg.

### Surgical subcutaneous implantation procedure.

Before surgery, the animals were anesthetized with ketamine-xylazine, and both blood as well as vaginal and rectal swabs were collected. For implants in the first three studies, two small incisions were made in the skin, and a blunt probe was gently inserted under the skin to ensure room for the implant. The implants were atraumatically and gently inserted between the shoulder blades, and the wound was sutured closed. In the case of the fourth study in rhesus macaques, a 4.5-mm trocar kit (Elemis Corp., Carson City, NV) was used to insert the devices, and the wound was sutured closed.

### Blood collection.

Blood samples were collected before the surgical subcutaneous implantation procedure from the ear vein (rabbits) and femoral vein (macaques) and then weekly for up to 12 weeks. Approximately 8 ml of blood was taken from the ear or femoral vein, transferred into EDTA-coated tubes, and immediately placed on ice. Additionally, 1 ml of blood was collected before the surgeries and every 4 weeks for complete blood counts and chemistries.

### Plasma isolation.

Whole-blood samples taken from the ear (rabbits) or femoral (macaques) vein were collected into EDTA-coated tubes and immediately placed on ice. The tubes were centrifuged (2,100 rpm for 20 min at 4°C) to separate plasma from the cells. Isolated plasma, present in the upper layer of the sample, was transferred to new tubes and stored at −80°C for further PK analysis.

### Cell isolation and TFV-DP extraction.

Rabbit heterophils and macaque mononuclear cells were isolated from peripheral blood obtained from the ear (rabbits) or femoral (macaques) vein. Briefly, following blood collection, the samples were placed immediately on ice and then centrifuged (2,100 rpm for 20 min at 4°C). Next, plasma was removed, and PBS (∼1 ml) was added to the remaining blood. The samples were then layered over lymphocyte separation medium and centrifuged for 20 min at 2,100 rpm to remove red blood cells. The layer containing the PBMCs was collected and transferred into 15-ml centrifuge tubes. Cells were pelleted by centrifugation (1,500 rpm for 7 min) and resuspended in 2 ml of PBS. Next, the cells were counted at a 1:10 dilution using Turk’s solution in a counting chamber. The cells were pelleted again (1,500 rpm for 7 min) and resuspended in 2 ml of ice-cold 70% methanol. This solution was then split into two 1-ml vials and stored at −80°C for further PK analysis.

### Histology.

Tissue samples located near the implantation site as well as samples of liver, lung, spleen, kidney, vagina, and rectum were collected, fixed in z-fix, embedded in paraffin, and cut into tissue sections. Paraffin-embedded specimens were stained with hematoxylin and eosin. Stained tissue sections were evaluated for signs of the presence and severity of inflammation. Tissue samples from naive animals were used as controls. Samples were blinded and scored by a trained pathologist.

### Histology and scoring algorithm.

We used a semiquantitative histological scoring system to identify and characterize the presence of cellular and tissue responses in high-power fields (HPFs) of slides of the peri-implant space. Table 6 displays the semiquantitative scoring system derived from recommendations of ISO 10993-6:2016 annex E (37). Blinded slides were provided to a pathologist for scoring. Cells were counted per HPF, and a table with scores was filled out as a summary from multiple slides along the length of each fixed implant (e.g., see Supplemental 4 in the supplemental material). Key characteristics that were evaluated were polymorphonuclear cells (heterophils in rabbits and neutrophils in macaques), lymphocytes, plasma cells, macrophages, giant cells, necrosis, capsule thickness, and tissue infiltrate (Table 6).

**TABLE 6:** Histological characteristic scoring scheme used to evaluate the cellular and tissue characteristics observed near the implants

Characteristic	Criterion for score of:					Reactive inflammation multiplier (*m_j_*_,_*_r_*)
	0	1	2	3	4	
Cells						
Polymorphonuclear cells	0/HPF	Rare, 1–5/HPF	5–10/HPF	Heavy infiltrate	Packed	2
Lymphocytes	0/HPF	Rare, 1–5/HPF	5–10/HPF	Heavy infiltrate	Packed	2
Plasma cells	0/HPF	Rare, 1–5/HPF	5–10/HPF	Heavy infiltrate	Packed	2
Macrophages	0/HPF	Rare, 1–5/HPF	5–10/HPF	Heavy infiltrate	Packed	2
Giant cells	0/HPF	Rare, 1–2/HPF	3–5/HPF	Heavy infiltrate	Sheets	2

Tissue						
Necrosis	0	Minimal	Mild	Moderate	Severe	2
Capsule thickness	0	Narrow band (<5 cells)	Moderate (5–10 cells)	Thick band (10–20 cells)	Extensive thick band	1
Tissue infiltrate	0	Minimal focal invasion of local tissue	Mild to multifocal inflammation in adjacent tissues	Moderate inflammation in adjacent tissues	Marked inflammation in adjacent tissues	1

We report the scores of the characteristics in two ways. In Table 1, we simply sum the cellular and tissue characteristic scores and take the average across all the implants in a group to compute the total histological characteristic score. Second, we computed an implant reactivity grade for each implant type tested using the following equation:

$${\bar{S}}_{\mathrm{pair}}=\frac{1}{N_{a}}\overset{N_{a}}{\underset{i=1}{\sum }}\overset{8}{\underset{j=1}{\sum }}S_{i,j,a}\times m_{j,r}-\frac{1}{N_{p}}\overset{N_{p}}{\underset{i=1}{\sum }}\overset{8}{\underset{j=1}{\sum }}S_{i,j,p}\times m_{j,r}$$

To compute the implant reactivity grade, we first summed the product of each histological characteristic score (*S_j_*_,_*_a_* for an active implant’s and *S_j_*_,_*_p_* for a placebo implant’s characteristic scores, with *j* equal to 1 to 8 for all 8 characteristics used in the histological analysis) and each characteristic’s reactive inflammation multiplier (*m_j_*_,_*_r_*) (Table 6) for each implant. This sum was then averaged over all implants of that type (*N_p_* for the number of placebos and *N_a_* for the number of active implants in each group). We call this score for each type the average implant reactivity score, which varies from 0 to 48. Inflammatory cellular infiltrate characteristics and tissue characteristics of necrosis receive an inflammatory reaction multiplier of 2 to represent the greater importance of inflammation in the endpoints of these studies. Next, the average implant reactivity scores for the active and placebo treatments were subtracted to compute the average placebo-adjusted implant reactivity score (${\bar{S}}_{\mathrm{pair}}$). Finally, the implant reactivity grade was determined by lookup as follows: minimal to no reaction (${\bar{S}}_{\mathrm{pair}}$ from 0.0 up to 2.9), slight reaction (${\bar{S}}_{\mathrm{pair}}$ from 3.0 up to 8.9), moderate reaction (${\bar{S}}_{\mathrm{pair}}$ from 9.0 up to 15.0), and severe reaction (${\bar{S}}_{\mathrm{pair}}$ of >15.1) as per the reported standard (37) (Supplemental 6 and Table S15).

### Extracting and analyzing TFV in rabbit plasma.

TFV was separated from rabbit plasma through liquid/liquid extraction and analyzed by ultraperformance liquid chromatography-tandem mass spectrometry (UPLC-MS/MS) analysis. All calibration standards and quality controls (QCs) were prepared using 100 μl of sterile rabbit plasma obtained from GeneTex (Irvine, CA), 50 μl of a 100.8 nM solution of TFV* in water, 250 μl of MS-grade acetonitrile, and 50 μl of TFV dissolved in MS-grade water. This solution was used to generate a standard curve from 0.2 nM to 2,000 nM with low, middle, and high QCs at 111 nM, 222 nM, and 1,111 nM. Samples were similarly prepared by spiking 100 μl of collected rabbit plasma with 50 μl of TFV*, 50 μl of MS-grade water, and 250 μl of MS-grade acetonitrile. Spiked solutions were mixed at 400 rpm for 10 min, centrifuged at 10,000 rpm for 10 min, and passed through a 0.2-μm nylon syringe filter tip into a centrifuge tube. Finally, 200 μl of each extract was transferred to a 2-ml, 96-well Nunc DeepWell plate (Thermo Scientific, Waltham, MA) and vacuum dried at 50°C for 2 h in a Savant SPD111V SpeedVac concentrator (Thermo Scientific, Waltham, MA). The final plate was reconstituted in 200 μl of MS-grade water, capped with a silicone mat (Axygen, Corning, NY), and mixed at 400 rpm for 30 min at 37°C.

Samples were analyzed by UPLC-MS/MS with 5-μl injection volumes on a Zorbax RRHD Eclipse Plus C_18_ column (2.1 by 50 mm, 1.8 μm; Agilent, Santa Clara, CA) using a Shimadzu Nexera X2 ultrahigh-performance liquid chromatography (UHPLC) system (Shimadzu, Columbia, MD) with a SciEx Qtrap 6500^+^ mass spectrometer (SciEx, Redwood City, CA). Analytes were separated by gradient at a flow rate of 0.75 ml/min using 0.5% acetic acid in water (mobile phase A) and 0.5% acetic acid in methanol (mobile phase B) over 3.55 min (gradient of 0% mobile phase B at 0 min, 0% mobile phase B at 0.5 min, 100% mobile phase B at 2.0 min, and 0% mobile phase B at 2.1 min). The column thermostat was held at 40°C, while the sample chamber in the autosampler was cooled to 15°C.

The SciEx detector was set to positive-ion mode. A multiple-reaction monitoring scan was used to detect transitions for TFV from *m/z* 288.1 to 176.1, for TFV* from *m/z* 293.1 to 181.2, for TAF from *m/z* 477.1 to 346.1, for monophenyl PMPA from *m/z* 364.1 to 176.2, and for PMPA monoamidate from *m/z* 401.2 to 270.1. The dwell time was set to 100 ms for all analytes except TFV*, which was set for 50 ms. The collision energies were set to 30 eV for TAF, 25 eV for PMPA monoamidate, and 34 eV for TFV, TFV*, and monophenyl PMPA. Curtain gas, nebulizer gas, and auxiliary gas were set to 25 lb/in^2^, 50 lb/in^2^, and 55 lb/in^2^, respectively. The ion spray voltage and source temperature were set to 5,500 V and 400°C. The declustering potential was set to 55 V. The entrance potential and collision cell exit potential were both set to 10 V. Data were acquired, processed, and quantified using Analyst software (SciEx, Redwood City, CA). TFV in samples was quantified by using linear regression of area-under-the-curve ratios of TFV and TFV* from standard preparations.

### Pharmacokinetic measurements.

Quantification of TAF, TFV, and TFV-DP in all matrices except TFV rabbit plasma (see above) was conducted by the Johns Hopkins University School of Medicine Clinical Pharmacology Analytical Laboratory, and measurements were conducted using previously described LC-MS/MS approaches (46). TFV-DP quantification in PBMCs and tissue was conducted using a previously described, indirect enzymatic approach (47). All assays were validated in accordance with FDA guidance for industry on bioanalytical method validation, and assay calibrators and QCs were prepared using human material (48). Assay lower limits of quantification were as follows: 0.31 ng/ml for plasma TFV, 0.03 ng/ml for plasma TAF, 0.05 ng/sample for tissue TFV, and 50 fmol or 5 fmol/sample for PBMC and tissue TFV-DP. TFV-DP concentrations were normalized to several cells tested, for final reporting as femtomoles per 10^6^ cells.

## Supplementary Material

Supplemental file 1
