# Supplementary material for: A Subcutaneous Implant of Tenofovir Alafenamide Fumarate Causes Local Inflammation and Tissue Necrosis in Rabbits and Macaques
Source: Antimicrob Agents Chemother. 2020 Feb 21;64(3):e01893-19. doi: 10.1128/AAC.01893-19 (PMC7038301; doi:10.1128/AAC.01893-19)
Supplement: Supplemental file 1 [file AAC.01893-19-s0001.pdf]

Supplemental Material Index

Supplimental 1. Methods of implant manufacturing and in vitro release testing 2

Supplemental 2. HPLC chromatograms for TFV, monophenyl PMPA, PMPA monoamidate, and TAF parent from a generation B TAF implant on days 5 and 63 of in vitro release testing....4

Supplemental 3. TFV-DP Tissue and TFV plasma levels in New Zealand White rabbits in generation A implants ...6

Supplemental 4. Work up of implants and 12 week histology reports in New Zealand White rabbits in generation A implants....18

Supplemental 5. 4 week and 12-week histology report of TAF generation B implants in rhesus macaques.........41

Supplemental 6. Implant reactivity grade summary....49

Supplemental 7. Molecular weight analysis of polymer wall material before and after in vivo exposure 49

Supplemental 8. TFV-DP tissue and TAF & TFV plasma levels in rhesus macaques with generation B implants ...51

Supplemental 9. Use of a trocar for implantation in a PK and safety study of the generation B implant in rhesus macaques....55

Supplimental 1. Methods of implant manufacturing and in vitro release testing

Implant manufacturing

Tecoflex™ EG-85A, and EG-85A:EG-93A (50:50 ratio) tubing was manufactured by hot melt extrusion, much as in Clark et al. (1). The EG-85A tubing was extruded on an ATR Plasti-Corder(R) single screw extruder (C.W. Brabender, South Hackensack, NJ), at a draw down ratio of 26.44 and draw balance ratio of 0.99 The zone temperatures for extrusion were 140/185/185/165/140 (all °C). The EG-85A:EG-93A (50:50 ratio) was first extruded into a rod using a twin screw extruder (C.W. Brabender, South Hackensack, NJ), to ensure homogeneity of the blend, with the zone temperatures set to 145/175/180/170/150 (all °C). The extruded rods were pelletized using a micro pelletizer (Randcastle Extrusion Systems Inc., Cedar Grove, NJ), and the pelletized material was extruded on the ATR plasticorder single screw extruder at a draw down ratio of 27.61 and draw balance ratio of 1.02, with the zone temperatures for extrusion set at 150/165/190/180/130 (all °C). The extruded tubing was sized for consistency. Tubing deviating by more than 10% from the required diameter and wall thickness was rejected. Tubing meeting the specifications were cut to length using a single-sided razor blade. 40% barium sulfate loaded EG-93A rods were manufactured by hot melt extrusion. Barium sulfate (40 wt%) was added to EG-93A and then extruded on the twin extruder. The zone temperatures for extrusion were 145/180/180/165/100 (all °C). The resulting extrudate was pelletized on the micro pelletizer. The 40% barium sulfate/EG93A pellets were then extruded at a diameter of 2.2 mm on the ATR plasticorder single screw extruder. The zone temperatures for extrusion were 155/180/180/150/120 (all °C). The 40% barium sulfate loaded EG-93A rods were cut into 3 mm long pellets using a single-sided razor blade. TAF was wet-granulated with NaCl at a ratio of 98:2 wt/wt with ethanol and the vacuum dried granulate was dry-coated with 2% magnesium stearate wt/wt. This mixture of powder was pressed into either 1.8- or 2.0-mm diameter pellets using a Natoli NP-RD10A (Natoli, St. Charles, MO) pellet press fitted with a 1.8 mm x 9.4 mm or 2.0 mm x 10 mm die/punch with the compression force set to 1000 lbs, and a fill depth of 3.3 mm. One end of the cut tubing was heat sealed using a Packworld PW2200 impulse sealer (Packworld, Nazareth, NJ) with the sealing conditions set with the following parameters: sealing temperature 120 °C, sealing time of 4 seconds, and percentage cooling before the clamp deactivates at 50%, and a sealing pressure of 60 psi. Pellets were loaded into the tubing. 1.8 mm or 2.0 mm diameter pellets of an equivalent amount of NaCl and magnesium stearate at a 1:1 ratio (wt/wt) were used for the placebo. For Generation B active and placebo implants, a 3 mm pellet of 40% barium sulfate compounded with the polyurethane Tecoflex EG-93A was also loaded into the tubing. The implant was then sealed at the other end using the heat sealer with the same impulse-sealing conditions described earlier. The ends were trimmed using surgical scissors. The total implant mass was recorded, and the implant placed in a polyethylene backed aluminum pouch (U-line, Pleasant Prairie, WI) which was heat sealed using an AIE300CA vacuum impulse sealer (American International Electric, City of Industry, CA). Implants were then annealed for 15 hours at 40 °C.

In vitro release testing of implants

In vitro release was conducted under sink conditions in 20 mL scintillation vials at 37˚C and 80 RPM in an I26 Incubator/Shaker (New Brunswick Scientific, Edison, NJ) using 20 mL of 1xPBS with 0.02% (w/v) sodium azide. Media was changed daily. Sink conditions were maintained at TAF concentrations < 1/10 the maximum solubility of the drug. TAF from *in vitro* samples was measured on a Zorbax Eclipse Plus C18 column (4.6x100 mm, 3.5 µm Agilent, Santa Clara, CA) and a Zorbax Eclipse Plus C18 guard column (4.6x12.5 mm, 5 µm Agilent, Santa Clara, CA) on an Agilent 1200 series HPLC attached to a UV based diode array detector at 260 nm (Agilent, Santa Clara, CA). TAF was separated by gradient elution using 20 mM ammonium acetate buffer (mobile phase A) and acetonitrile (mobile phase B) over 12.5 minutes (gradient t=0: %B=0, t=8: %B=55, t= 9.6: %B=55, t=9.7: %B= 0) at 0.7 mL/min. The column thermostat was set to 25 °C and samples were injected at ambient temperature. Data were acquired and processed using Agilent Chemstation software (Agilent, Santa Clara, CA). Calibration curves were prepared using on column amounts of TAF (40 and 800 µg/mL) and TFV (10 and 100 µg/mL) standards injected at 1, 2, 5, and 10 µL volumes. Sample concentrations were determined using the linear regression of calibration curves to measure TAF and its TFV-related species (PMPA monoamidate, monophenyl PMPA, and TFV). The TAF calibration curve was used to quantitate TAF and monophenyl PMPA, and the TFV calibration curve was used to quantitate TFV and PMPA monoamidate. The sum of TAF, TFV-related species (PMPA monoamidate and monophenyl PMPA), and TFV were quantified as TAF equivalent and reported as the total mass of TAF release.

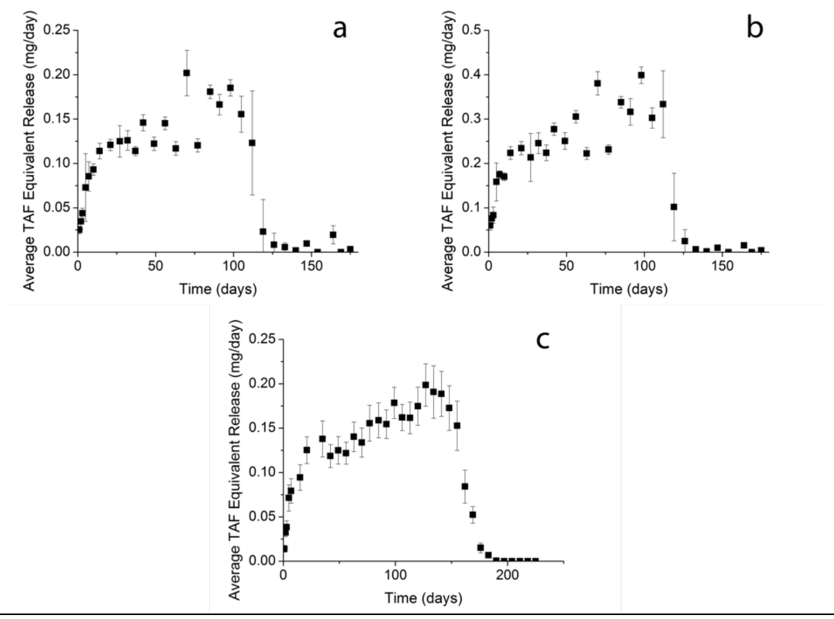

Figure S1. (a) TAF Gen A 0.8 cm lumen length implant in vitro release (n=10) error bars ± SD. (b) TAF Gen A 1.6 cm lumen length implant in vitro release (n=9) error bars ± SD. (c) TAF Gen B implant in vitro release (n=10) error bars ± SD.

Supplemental 2. HPLC chromatograms for TFV, monophenyl PMPA, PMPA monoamidate, and TAF parent from a generation B TAF implant on days 5 and 63 of in vitro release testing

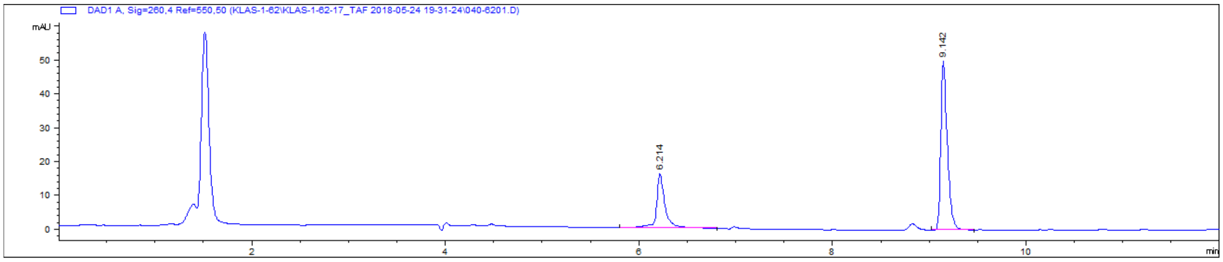

Figure S2. Chromatograph showing relative peaks areas and retention times for PMPA monoamidate (RT=6.21 min) and TAF parent (RT=9.14 min) from a generation B TAF implant on day 5 of *in vitro* release testing

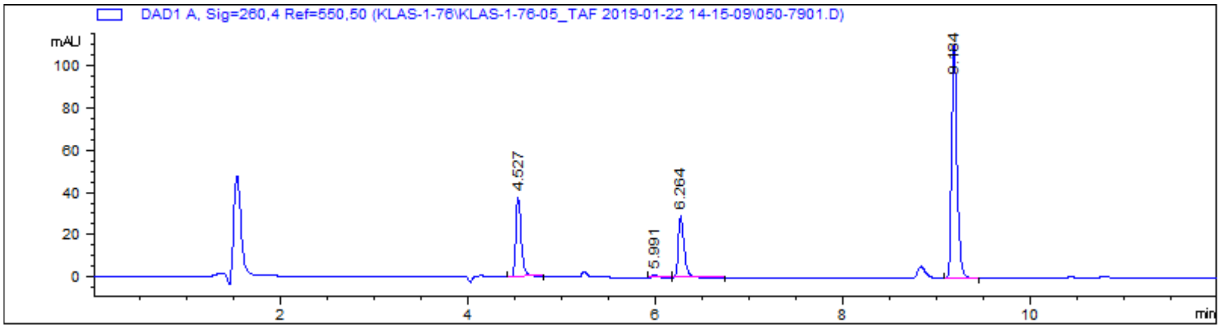

Figure S3. Chromatograph showing relative peak areas and retention times for TFV (RT=4.53 min), monophenyl PMPA (RT=5.99 min), PMPA monoamidate (RT=6.26 min), and TAF parent (RT=9.184 min) from a generation B TAF implant on day 63 of *in vitro* release testing

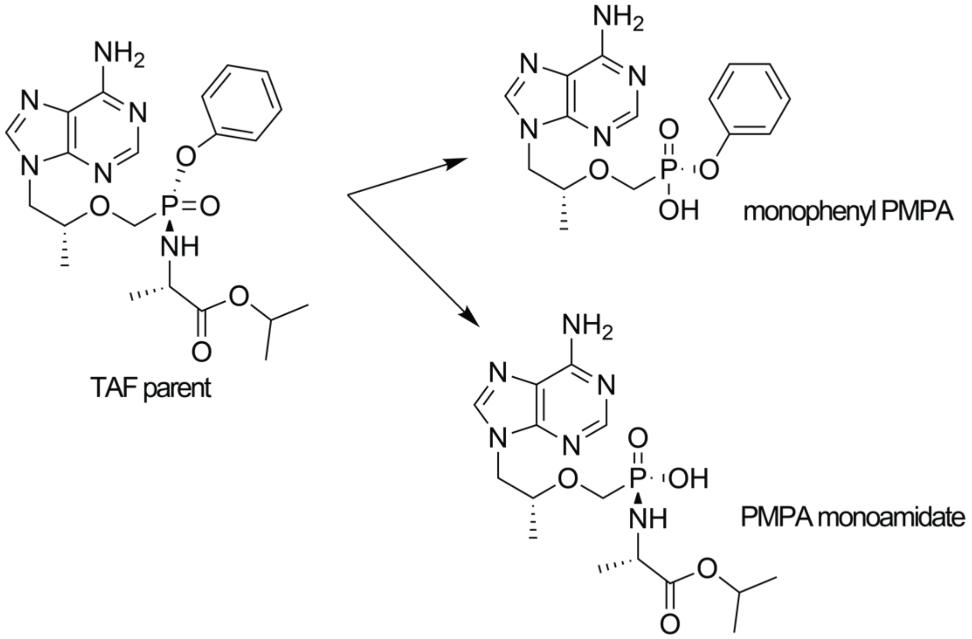

Figure S4. TAF parent is tenofovir alafenamide without the fumarate salt, which undergoes pH-dependent hydrolysis into two main related substances monophenyl PMPA and PMPA monoamidate.

Day	TAF (µg/day, using hemifumarate MW)	PMPA monoamidate (µg/day)	monophenyl PMPA (µg/day)	TFV (µg/day)
1	9.4 ± 3.6	3.5 ± 0.2	0.0 ± 0.0	0.0 ± 0.0
2	25.4 ± 4.0	5.1 ± 0.5	0.0 ± 0.0	0.0 ± 0.0
3	28.7 ± 5.9	6.4 ± 0.8	0.0 ± 0.0	0.7 ± 0.0
5	54.0 ± 11.2	11.8 ± 3.4	0.0 ± 0.0	0.9 ± 1.2
7	59.8 ± 10.2	12.7 ± 1.7	0.0 ± 0.0	1.4 ± 0.9
15	50.6 ± 7.7	28.8 ± 4.0	0.0 ± 0.0	2.8 ± 0.8
21	91.4 ± 10.9	24.6 ± 2.9	0.0 ± 0.0	0.6 ± 0.3
35	81.7 ± 11.0	26.0 ± 8.3	0.0 ± 0.0	14.4 ± 1.7
42	66.1 ± 8.6	17.5 ± 10.2	0.0 ± 0.0	19.1 ± 9.9
49	67.5 ± 7.6	12.6 ± 3.9	0.0 ± 0.0	25.2 ± 2.5
56	58.5 ± 5.2	10.6 ± 1.6	0.0 ± 0.0	30.0 ± 3.2
63	92.0 ± 11.0	17.1 ± 3.6	0.1 ± 0.1	16.2 ± 2.9
70	64.9 ± 10.0	17.5 ± 3.2	0.0 ± 0.0	27.9 ± 3.3
77	75.7 ± 12.3	35.5 ± 13.8	0.0 ± 0.0	20.4 ± 12.8
85	90.1 ± 21.4	31.0 ± 11.9	0.0 ± 0.0	17.2 ± 2.5
92	89.7 ± 10.6	30.1 ± 3.8	0.0 ± 0.0	15.8 ± 1.2
99	97.2 ± 13.6	25.6 ± 4.0	0.0 ± 0.0	27.6 ± 2.4
106	122.1 ± 9.9	6.5 ± 1.2	0.0 ± 0.0	18.3 ± 2.0

113	114.3 ± 9.3	30.4 ± 12.0	0.0 ± 0.0	5.4 ± 4.9
120	138.5 ± 16.9	9.8 ± 1.6	0.0 ± 0.1	13.5 ± 1.9
127	111.9 ± 11.7	7.1 ± 1.1	0.0 ± 0.0	43.2 ± 5.7
134	134.8 ± 21.8	14.8 ± 2.0	0.0 ± 0.1	20.5 ± 3.7
141	144.5 ± 19.3	13.3 ± 1.8	0.0 ± 0.0	14.9 ± 2.1
148	128.5 ± 17.8	13.7 ± 2.1	0.0 ± 0.0	15.3 ± 2.5
155	113.2 ± 19.1	13.8 ± 3.2	0.0 ± 0.0	13.2 ± 2.3
162	63.2 ± 13.4	9.5 ± 1.6	0.0 ± 0.0	8.1 ± 1.4
169	41.5 ± 6.0	10.5 ± 2.0	0.0 ± 0.0	2.6 ± 2.6
176	12.4 ± 2.2	3.5 ± 1.4	0.0 ± 0.0	4.3 ± 1.2
183	12.7 ± 2.2	2.6 ± 1.4	0.0 ± 0.0	0.6 ± 0.3
190	6.1 ± 0.8	0.6 ± 0.5	0.0 ± 0.0	0.0 ± 0.0
197	4.3 ± 0.7	0.2 ± 0.4	0.0 ± 0.0	0.0 ± 0.0
204	1.7 ± 0.3	0.0 ± 0.0	0.0 ± 0.0	0.0 ± 0.0
211	0.6 ± 0.4	0.0 ± 0.0	0.0 ± 0.0	0.0 ± 0.0
218	0.1 ± 0.2	0.0 ± 0.0	0.0 ± 0.0	0.0 ± 0.0
225	0.0 ± 0.0	0.0 ± 0.0	0.0 ± 0.0	0.0 ± 0.0

[a]Using tenofovir alafenamide hemifumarate molecular weight

Supplemental 3. TFV-DP Tissue and TFV plasma levels in New Zealand White rabbits in generation A implants

Table S2. Generation A NZW rabbit TFV-DP tissue levels

Animal ID	*In vitro* release rate (mg/day)	Time Point (weeks)	Specimen Type	Final [TFVdp] (fmol/mg)
M29035	0.13	4	Implant Tissue Left	2.06
M29036	0.13	4	Implant Tissue Left	BLQ
M29037	0.26	4	Implant Tissue Left	3.27
M29038	0.26	4	Implant Tissue Left	BLQ
M29039	0.48	4	Implant Tissue Left	32.22
M29040	0.48	4	Implant Tissue Left	1537.81
M29041	0.72	4	Implant Tissue Left	11930.48
M29042	0.72	4	Implant Tissue Left	168.26
M29043	0.72	4	Implant Tissue Left	11222.4
M29044	0	12	Implant Tissue Left	BLQ
M29045	0	12	Implant Tissue Left	BLQ
M29046	0	12	Implant Tissue Left	BLQ

M29047	0.13	12	Implant Tissue Left	0.859
M29048	0.13	12	Implant Tissue Left	1.62
M29049	0.13	12	Implant Tissue Left	0.851
M29050	0.13	12	Implant Tissue Left	1.587
M29051	0.13	12	Implant Tissue Left	5.66
M29052	0.26	12	Implant Tissue Left	4102
M29053	0.26	12	Implant Tissue Left	2.36
M29054	0.26	12	Implant Tissue Left	2938
M29055	0.26	12	Implant Tissue Left	10.5
M29056	0.26	12	Implant Tissue Left	114.6
M29057	0.48	12	Implant Tissue Left	44509
M29058	0.48	12	Implant Tissue Left	8.58
M29059	0.48	12	Implant Tissue Left	3427
M29060	0.48	12	Implant Tissue Left	30.7
M29062	0.72	12	Implant Tissue Left	16136
M29063	0.72	12	Implant Tissue Left	10386
M29064	0.72	12	Implant Tissue Left	120
M29036	0.13	4	Implant Tissue Right	2.38
M29037	0.13	4	Implant Tissue Right	BLQ
M29038	0.26	4	Implant Tissue Right	BLQ
M29039	0.26	4	Implant Tissue Right	948.60
M29040	0.48	4	Implant Tissue Right	2032.36
M29041	0.48	4	Implant Tissue Right	7291.24
M29042	0.72	4	Implant Tissue Right	305.66
M29044	0	12	Implant Tissue Right	BLQ
M29045	0	12	Implant Tissue Right	BLQ
M29046	0	12	Implant Tissue Right	BLQ
M29047	0.13	12	Implant Tissue Right	214
M29048	0.13	12	Implant Tissue Right	1644
M29049	0.13	12	Implant Tissue Right	126
M29050	0.13	12	Implant Tissue Right	69941
M29051	0.13	12	Implant Tissue Right	6371
M29052	0.26	12	Implant Tissue Right	1.31
M29053	0.26	12	Implant Tissue Right	86.3
M29054	0.26	12	Implant Tissue Right	56.7
M29055	0.26	12	Implant Tissue Right	9132
M29056	0.26	12	Implant Tissue Right	30.3
M29057	0.48	12	Implant Tissue Right	1726
M29058	0.48	12	Implant Tissue Right	27.5
M29059	0.48	12	Implant Tissue Right	8.18
M29060	0.48	12	Implant Tissue Right	7.75

M29061	0.72	12	Implant Tissue Right	12113
M29062	0.72	12	Implant Tissue Right	6422
M29064	0.72	12	Implant Tissue Right	360
M29035	0.13	4	Vaginal Tissue	3.67
M29036	0.13	4	Vaginal Tissue	4.25
M29037	0.26	4	Vaginal Tissue	8.08
M29038	0.26	4	Vaginal Tissue	3.29
M29039	0.48	4	Vaginal Tissue	34.81
M29040	0.48	4	Vaginal Tissue	17.14
M29041	0.72	4	Vaginal Tissue	169.36
M29042	0.72	4	Vaginal Tissue	63.35
M29043	0.72	4	Vaginal Tissue	98.74
M29044	0	12	Vaginal Tissue	BLQ
M29045	0	12	Vaginal Tissue	BLQ
M29046	0	12	Vaginal Tissue	BLQ
M29047	0.13	12	Vaginal Tissue	7.05
M29048	0.13	12	Vaginal Tissue	7.09
M29049	0.13	12	Vaginal Tissue	7.74
M29050	0.13	12	Vaginal Tissue	13.3
M29051	0.13	12	Vaginal Tissue	2.83
M29052	0.26	12	Vaginal Tissue	3.58
M29053	0.26	12	Vaginal Tissue	17
M29054	0.26	12	Vaginal Tissue	19.7
M29055	0.26	12	Vaginal Tissue	14.9
M29056	0.26	12	Vaginal Tissue	8.49
M29057	0.48	12	Vaginal Tissue	62.1
M29058	0.48	12	Vaginal Tissue	14.8
M29059	0.48	12	Vaginal Tissue	24.3
M29060	0.48	12	Vaginal Tissue	22.6
M29061	0.48	12	Vaginal Tissue	51.6
M29062	0.72	12	Vaginal Tissue	73
M29063	0.72	12	Vaginal Tissue	77.6
M29064	0.72	12	Vaginal Tissue	37.1
M29035	0.13	4	Rectal Tissue	2.79
M29036	0.13	4	Rectal Tissue	3.96
M29037	0.26	4	Rectal Tissue	6.33
M29038	0.26	4	Rectal Tissue	BLQ
M29039	0.48	4	Rectal Tissue	49.95
M29040	0.48	4	Rectal Tissue	10.22
M29042	0.72	4	Rectal Tissue	31.45
M29043	0.72	4	Rectal Tissue	46.97

M29044	0	12	Rectal Tissue	BLQ
M29045	0	12	Rectal Tissue	BLQ
M29046	0	12	Rectal Tissue	BLQ
M29047	0.13	12	Rectal Tissue	3.32
M29048	0.13	12	Rectal Tissue	2
M29049	0.13	12	Rectal Tissue	2.63
M29050	0.13	12	Rectal Tissue	4.26
M29051	0.13	12	Rectal Tissue	3.44
M29052	0.26	12	Rectal Tissue	3.08
M29053	0.26	12	Rectal Tissue	23.4
M29054	0.26	12	Rectal Tissue	8.49
M29055	0.26	12	Rectal Tissue	13.6
M29056	0.26	12	Rectal Tissue	7.08
M29057	0.48	12	Rectal Tissue	43.9
M29058	0.48	12	Rectal Tissue	30.1
M29059	0.48	12	Rectal Tissue	18.9
M29060	0.48	12	Rectal Tissue	6.51
M29061	0.48	12	Rectal Tissue	25.3
M29062	0.72	12	Rectal Tissue	23.5
M29063	0.72	12	Rectal Tissue	47.1
M29064	0.72	12	Rectal Tissue	29.6

Table S3. Gen A plasma TFV concentrations in NZW rabbits.

**Specimen ID**	***In vitro* release rate (mg/day)**	**Time Point (Week)**	**Calculated Concentration (ng/mL)**
M29035	0.13	Week 0	BLQ
M29035	0.13	Week 1	BLQ
M29035	0.13	Week 2	1.73
M29035	0.13	Week 3	BLQ
M29035	0.13	Week 4	BLQ
M29036	0.13	Week 0	BLQ
M29036	0.13	Week 1	BLQ
M29036	0.13	Week 2	BLQ
M29036	0.13	Week 3	BLQ
M29036	0.13	Week 4	BLQ
M29037	0.26	Week 0	BLQ
M29037	0.26	Week 1	4.33
M29037	0.26	Week 2	2.16
M29037	0.26	Week 3	2.8
M29037	0.26	Week 4	4.36

M29038	0.26	Week 0	BLQ
M29038	0.26	Week 1	1.98
M29038	0.26	Week 2	BLQ
M29038	0.26	Week 3	1.89
M29038	0.26	Week 4	1.65
M29039	0.48	Week 0	BLQ
M29039	0.48	Week 1	4.77
M29039	0.48	Week 2	4.97
M29039	0.48	Week 3	4.76
M29039	0.48	Week 4	4.79
M29040	0.48	Week 0	BLQ
M29040	0.48	Week 1	4.49
M29040	0.48	Week 2	3.51
M29040	0.48	Week 3	4.7
M29040	0.48	Week 4	5.16
M29041	0.72	Week 0	5.36
M29041	0.72	Week 1	4.95
M29041	0.72	Week 2	4.93
M29041	0.72	Week 3	6.45
M29041	0.72	Week 4	5.62
M29042	0.72	Week 0	BLQ
M29042	0.72	Week 1	4.07
M29042	0.72	Week 2	4.42
M29042	0.72	Week 3	5.43
M29042	0.72	Week 4	5.91
M29043	0.72	Week 0	BLQ
M29043	0.72	Week 1	4.9
M29043	0.72	Week 2	6.01
M29043	0.72	Week 3	6.75
M29043	0.72	Week 4	5.78
M29044	Placebo	Week 0	BLQ
M29044	Placebo	Week 1	BLQ
M29044	Placebo	Week 2	BLQ
M29044	Placebo	Week 3	BLQ
M29044	Placebo	Week 4	BLQ
M29044	Placebo	Week 5	BLQ
M29044	Placebo	Week 6	BLQ
M29044	Placebo	Week 7	BLQ

M29044	Placebo	Week 8	BLQ
M29044	Placebo	Week 9	BLQ
M29044	Placebo	Week 10	BLQ
M29044	Placebo	Week 11	BLQ
M29044	Placebo	Week 12	BLQ
M29045	Placebo	Week 0	BLQ
M29045	Placebo	Week 1	BLQ
M29045	Placebo	Week 2	BLQ
M29045	Placebo	Week 3	BLQ
M29045	Placebo	Week 4	11.5
M29045	Placebo	Week 6	BLQ
M29045	Placebo	Week 7	BLQ
M29045	Placebo	Week 8	BLQ
M29045	Placebo	Week 10	BLQ
M29045	Placebo	Week 11	BLQ
M29045	Placebo	Week 12	BLQ
M29046	Placebo	Week 0	BLQ
M29046	Placebo	Week 1	BLQ
M29046	Placebo	Week 2	BLQ
M29046	Placebo	Week 3	BLQ
M29046	Placebo	Week 4	BLQ
M29046	Placebo	Week 5	BLQ
M29046	Placebo	Week 6	BLQ
M29046	Placebo	Week 7	BLQ
M29046	Placebo	Week 8	BLQ
M29046	Placebo	Week 9	BLQ
M29046	Placebo	Week 10	BLQ
M29046	Placebo	Week 11	BLQ
M29046	Placebo	Week 12	BLQ
M29047	0.13	Week 0	BLQ
M29047	0.13	Week 1	BLQ
M29047	0.13	Week 2	2.15
M29047	0.13	Week 3	1.67
M29047	0.13	Week 4	BLQ
M29047	0.13	Week 5	BLQ
M29047	0.13	Week 6	BLQ
M29047	0.13	Week 7	3.29
M29047	0.13	Week 8	2.29
M29047	0.13	Week 9	2.31

M29047	0.13	Week 10	2.22
M29047	0.13	Week 11	1.79
M29047	0.13	Week 12	BLQ
M29048	0.13	Week 0	BLQ
M29048	0.13	Week 1	BLQ
M29048	0.13	Week 2	BLQ
M29048	0.13	Week 3	BLQ
M29048	0.13	Week 4	BLQ
M29048	0.13	Week 5	2.06
M29048	0.13	Week 6	2.09
M29048	0.13	Week 7	1.92
M29048	0.13	Week 8	BLQ
M29048	0.13	Week 9	BLQ
M29048	0.13	Week 10	5.23
M29048	0.13	Week 11	1.67
M29048	0.13	Week 12	BLQ
M29049	0.13	Week 0	BLQ
M29049	0.13	Week 1	BLQ
M29049	0.13	Week 2	BLQ
M29049	0.13	Week 3	BLQ
M29049	0.13	Week 4	BLQ
M29049	0.13	Week 5	1.55
M29049	0.13	Week 6	3.61
M29049	0.13	Week 7	BLQ
M29049	0.13	Week 8	BLQ
M29049	0.13	Week 9	3.3
M29049	0.13	Week 10	3.2
M29049	0.13	Week 11	BLQ
M29049	0.13	Week 12	BLQ
M29050	0.13	Week 0	BLQ
M29050	0.13	Week 1	BLQ
M29050	0.13	Week 2	1.68
M29050	0.13	Week 3	BLQ
M29050	0.13	Week 4	BLQ
M29050	0.13	Week 5	2.73
M29050	0.13	Week 6	3.02
M29050	0.13	Week 7	1.82
M29050	0.13	Week 8	BLQ
M29050	0.13	Week 9	1.65

M29050	0.13	Week 10	19.7
M29050	0.13	Week 11	BLQ
M29050	0.13	Week 12	BLQ
M29051	0.13	Week 0	BLQ
M29051	0.13	Week 1	BLQ
M29051	0.13	Week 2	1.93
M29051	0.13	Week 3	1.53
M29051	0.13	Week 4	BLQ
M29051	0.13	Week 5	BLQ
M29051	0.13	Week 6	1.59
M29051	0.13	Week 7	BLQ
M29051	0.13	Week 8	BLQ
M29051	0.13	Week 9	BLQ
M29051	0.13	Week 10	10.1
M29051	0.13	Week 11	2.48
M29051	0.13	Week 12	BLQ
M29052	0.26	Week 0	BLQ
M29052	0.26	Week 1	BLQ
M29052	0.26	Week 2	4.82
M29052	0.26	Week 3	2.55
M29052	0.26	Week 4	2.34
M29052	0.26	Week 5	4.47
M29052	0.26	Week 6	4.36
M29052	0.26	Week 7	3.55
M29052	0.26	Week 8	2.85
M29052	0.26	Week 9	3.39
M29052	0.26	Week 10	4.38
M29052	0.26	Week 11	2.64
M29052	0.26	Week 12	BLQ
M29053	0.26	Week 0	BLQ
M29053	0.26	Week 1	2.02
M29053	0.26	Week 2	2.38
M29053	0.26	Week 3	3.08
M29053	0.26	Week 4	1.94
M29053	0.26	Week 5	2.95
M29053	0.26	Week 6	3.32
M29053	0.26	Week 7	2.19
M29053	0.26	Week 8	1.84

M29053	0.26	Week 9	2.78
M29053	0.26	Week 10	7.32
M29053	0.26	Week 11	3.66
M29053	0.26	Week 12	2.47
M29054	0.26	Week 0	BLQ
M29054	0.26	Week 1	2.01
M29054	0.26	Week 2	4.04
M29054	0.26	Week 3	2.96
M29054	0.26	Week 4	2.78
M29054	0.26	Week 5	2.86
M29054	0.26	Week 6	5.72
M29054	0.26	Week 7	2.29
M29054	0.26	Week 8	2.52
M29054	0.26	Week 9	2.98
M29054	0.26	Week 10	7.99
M29054	0.26	Week 11	3.55
M29054	0.26	Week 12	1.53
M29055	0.26	Week 0	BLQ
M29055	0.26	Week 1	3.93
M29055	0.26	Week 2	4.28
M29055	0.26	Week 3	2.42
M29055	0.26	Week 5	4.84
M29055	0.26	Week 6	3.77
M29055	0.26	Week 7	2.41
M29055	0.26	Week 9	2.29
M29055	0.26	Week 10	1.59
M29055	0.26	Week 12	2.53
M29056	0.26	Week 0	BLQ
M29056	0.26	Week 1	2.53
M29056	0.26	Week 2	3
M29056	0.26	Week 3	3.03
M29056	0.26	Week 4	2.68
M29056	0.26	Week 5	7.26
M29056	0.26	Week 6	4.32
M29056	0.26	Week 7	2.5
M29056	0.26	Week 8	2.78
M29056	0.26	Week 9	3.17
M29056	0.26	Week 11	3.15
M29056	0.26	Week 12	3.54

M29057	0.48	Week 0	BLQ
M29057	0.48	Week 1	7.5
M29057	0.48	Week 2	6.72
M29057	0.48	Week 3	6.06
M29057	0.48	Week 4	5.75
M29057	0.48	Week 5	5.76
M29057	0.48	Week 6	7.59
M29057	0.48	Week 7	5.7
M29057	0.48	Week 8	5.8
M29057	0.48	Week 9	7.26
M29057	0.48	Week 10	6.46
M29057	0.48	Week 11	9.23
M29057	0.48	Week 12	5.28
M29058	0.48	Week 0	BLQ
M29058	0.48	Week 1	5.52
M29058	0.48	Week 2	3.58
M29058	0.48	Week 3	4.21
M29058	0.48	Week 4	3.65
M29058	0.48	Week 5	4.49
M29058	0.48	Week 6	7.43
M29058	0.48	Week 7	6.27
M29058	0.48	Week 8	6.69
M29058	0.48	Week 9	6.51
M29058	0.48	Week 10	6.26
M29058	0.48	Week 11	7.64
M29058	0.48	Week 12	7.55
M29059	0.48	Week 1	4.63
M29059	0.48	Week 3	7.22
M29059	0.48	Week 4	7
M29059	0.48	Week 5	6.56
M29059	0.48	Week 6	8.94
M29059	0.48	Week 7	5.27
M29059	0.48	Week 8	5.93
M29059	0.48	Week 9	8.54
M29059	0.48	Week 10	BLQ
M29059	0.48	Week 12	1.9
M29060	0.48	Week 0	BLQ
M29060	0.48	Week 1	2.74
M29060	0.48	Week 2	2.45

M29060	0.48	Week 3	4.17
M29060	0.48	Week 4	3.43
M29060	0.48	Week 5	4.96
M29060	0.48	Week 6	5.92
M29060	0.48	Week 7	4.37
M29060	0.48	Week 8	4.44
M29060	0.48	Week 9	5.65
M29060	0.48	Week 10	12.2
M29060	0.48	Week 11	6.08
M29060	0.48	Week 12	5.03
M29061	0.48	Week 0	BLQ
M29061	0.48	Week 1	3.49
M29061	0.48	Week 2	7.17
M29061	0.48	Week 3	5.99
M29061	0.48	Week 4	6.76
M29061	0.48	Week 5	7.88
M29061	0.48	Week 6	9.38
M29061	0.48	Week 7	6.09
M29061	0.48	Week 8	7.47
M29061	0.48	Week 9	10.2
M29061	0.48	Week 10	4
M29061	0.48	Week 11	6.67
M29061	0.48	Week 12	8.28
M29062	0.72	Week 0	BLQ
M29062	0.72	Week 1	5.29
M29062	0.72	Week 2	7.24
M29062	0.72	Week 3	6.47
M29062	0.72	Week 4	8.2
M29062	0.72	Week 5	11.3
M29062	0.72	Week 6	8.1
M29062	0.72	Week 7	8.04
M29062	0.72	Week 8	6.55
M29062	0.72	Week 9	5.35
M29062	0.72	Week 10	10.4
M29062	0.72	Week 11	7.33
M29062	0.72	Week 12	5.71
M29063	0.72	Week 0	BLQ
M29063	0.72	Week 1	4.48
M29063	0.72	Week 2	5.5

M29063	0.72	Week 3	5.63
M29063	0.72	Week 4	8.68
M29063	0.72	Week 5	8.4
M29063	0.72	Week 6	11.1
M29063	0.72	Week 7	7.29
M29063	0.72	Week 8	8.74
M29063	0.72	Week 9	6.82
M29063	0.72	Week 10	7.95
M29063	0.72	Week 11	6.32
M29063	0.72	Week 12	7.97
M29064	0.72	Week 0	BLQ
M29064	0.72	Week 1	4.22
M29064	0.72	Week 2	5.81
M29064	0.72	Week 3	6.83
M29064	0.72	Week 4	6.08
M29064	0.72	Week 5	11.5
M29064	0.72	Week 6	7.72
M29064	0.72	Week 7	5.58
M29064	0.72	Week 8	7.92
M29064	0.72	Week 9	7.74
M29064	0.72	Week 10	7.23
M29064	0.72	Week 11	5.41
M29064	0.72	Week 12	3.46

Supplemental 4. Work up of implants and 12 week histology reports in New Zealand White rabbits in generation A implants

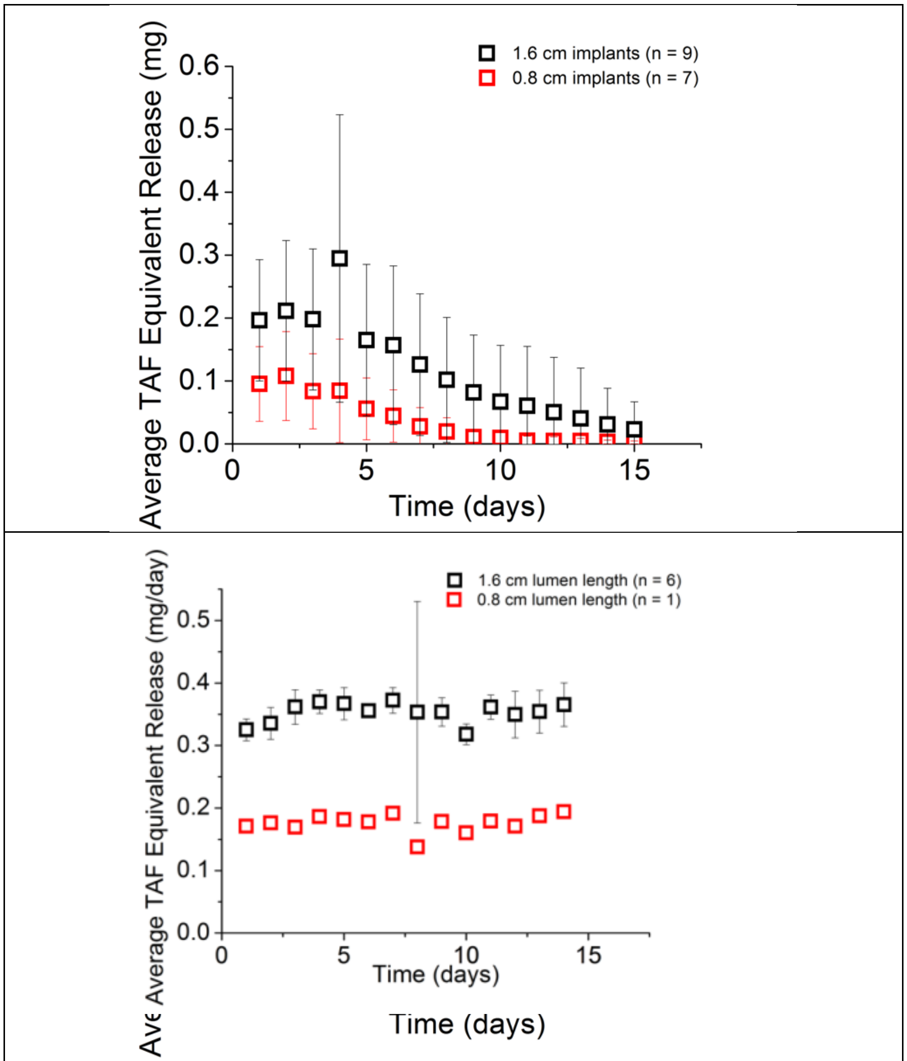

Figure S5. Generation A Implants not selected for histology were removed intact from NZW rabbits (top) at 12 weeks and rhesus macaques (bottom) at 12 weeks and placed on *in vitro* release to verify that expected release was being achieved.

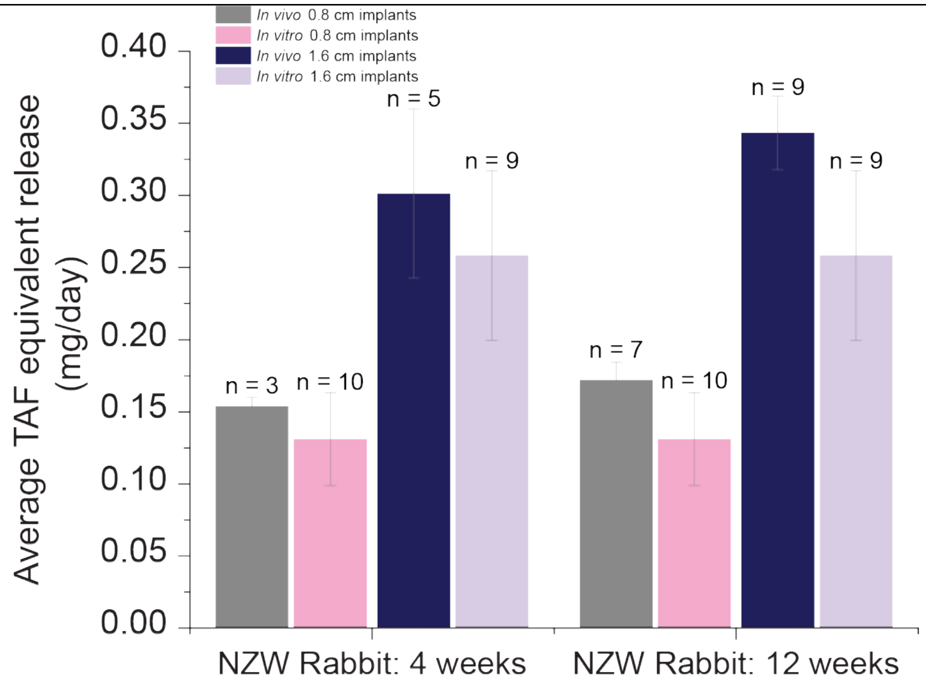

Figure S6. Comparison of average daily *in vitro* release over 7 to 91 days and the estimated *in vivo* release in NZW rabbits received at four weeks (left) and 12 weeks (right) from TAF Generation A implants.

Placebos

Table S4. Histological characteristic scores from four placebo implants in rabbits M29044 and M29045.

	**Response**			
Animal	**M29044**		**M29045**	
Implant	Left	Right	Left	Right
Polymorphonuclear cells	0	0	0	0
Lymphocytes	0	0	0	0
Plasma cells	0	0	0	0
Macrophages	0	0	0	0
Giant cells	0	0	0	0
Necrosis	0	0	0	0
Capsule thickness	2	2	1	1
Tissue infiltrate	0	0	0	0
Other				

**Subtotal**	**2**	**2**	**1**	**1**

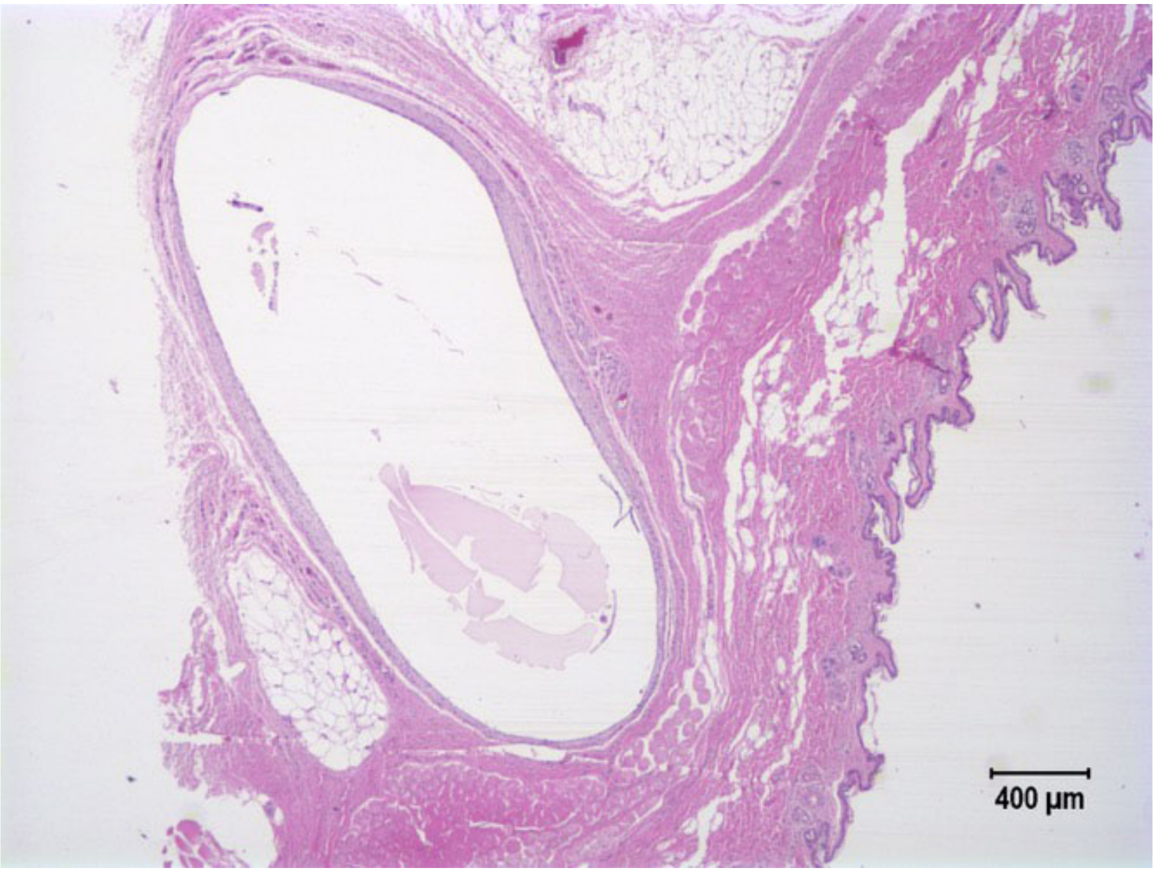

Figure S7. NZW Rabbit M29044. Placebo implant. Minimal inflammation detected in or around either implant. There is a thin fibrous tissue capsule around implants, but no inflammation observed in any of 8 sections. No significant lesions (NSL) are observed in the kidney, liver, spleen, lung, vagina or rectum.

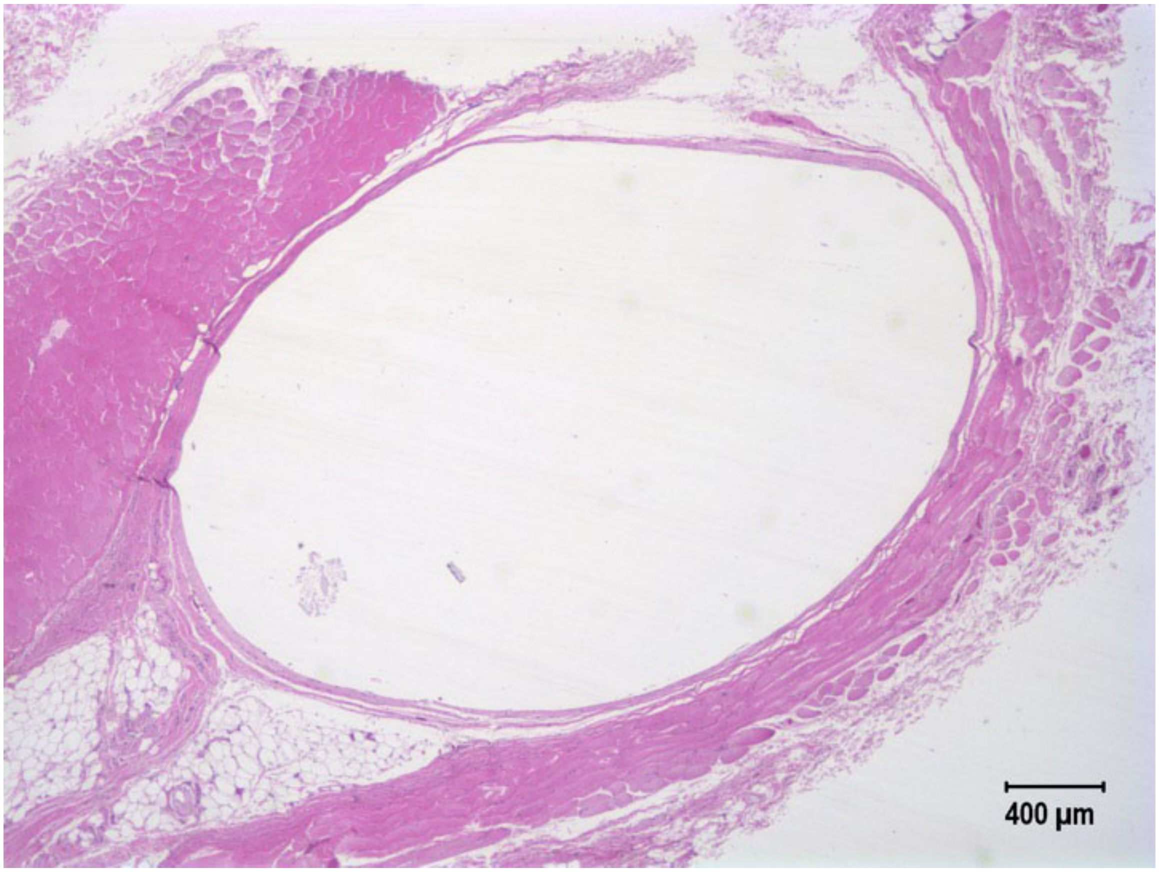

Figure S8. NZW Rabbit M29045. Placebo implant. Minimal inflammation in either implant. Thin fibrous tissue capsule around implants but no inflammation in any of 8 sections. NSL in kidney, liver, spleen, lung, vagina or rectum.

Group 1: *In vitro* release rate 0.13 mg/day

Table S5. Histological characteristic scores from rabbits M29047 – M29051. Each rabbit received a 0.8 cm active implant (right) and a contralateral placebo (left).

	**Response**							
**Animal**	**M29047**		**M29048**	**M29049**		**M29050**	**M29051**	
Implant	Left	Right	Left	Left	Right	Left	Left	Right
Polymorphonuclear cells	0	2	1	0	0	0	0	2
Lymphocytes	0	3	3	0	4	0	3	3
Plasma cells	0	3	3	0	3	0	1	3
Macrophages	1	3	3	0	3	0	1	3
Giant cells	0	0	0	0	1	0	0	1
Necrosis	0	1	3	0	3	0	0	2
Capsule thickness	2	4	3	2	4	2	1	2
Tissue infiltrate	0	2	1	0	2	0	1	1
Other								
**Overall total**	**3**	**18**	**17**	**2**	**18**	**2**	**7**	**17**

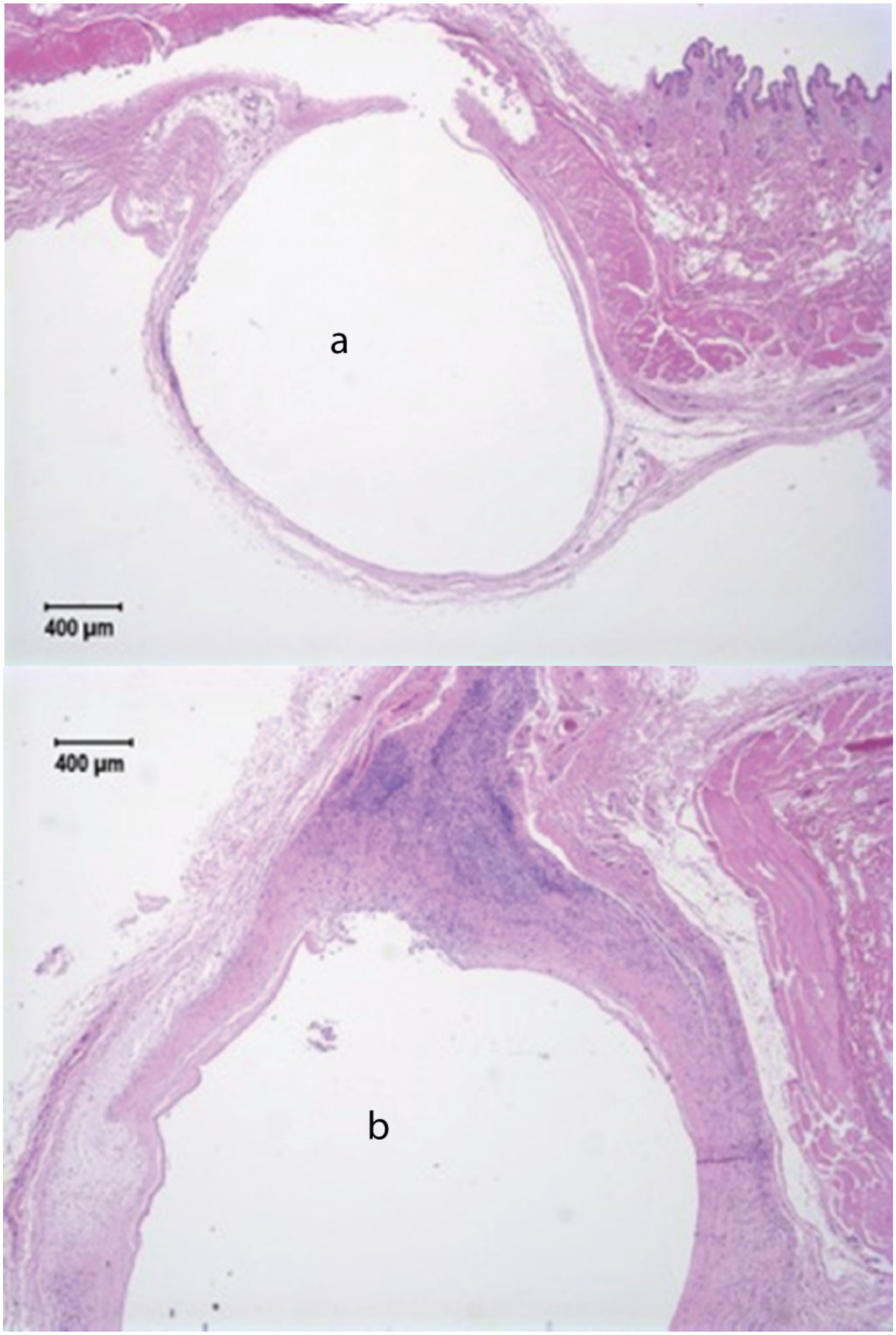

Figure S9. NZW Rabbit M29047**.** In vitro release rate 0.13 mg/day. Sections from left (a) usually have no inflammation although one section has mild to moderate inflammation. All

sections from the right (b) show moderate inflammation. NSL in kidney, liver, spleen, lung, vagina or rectum.

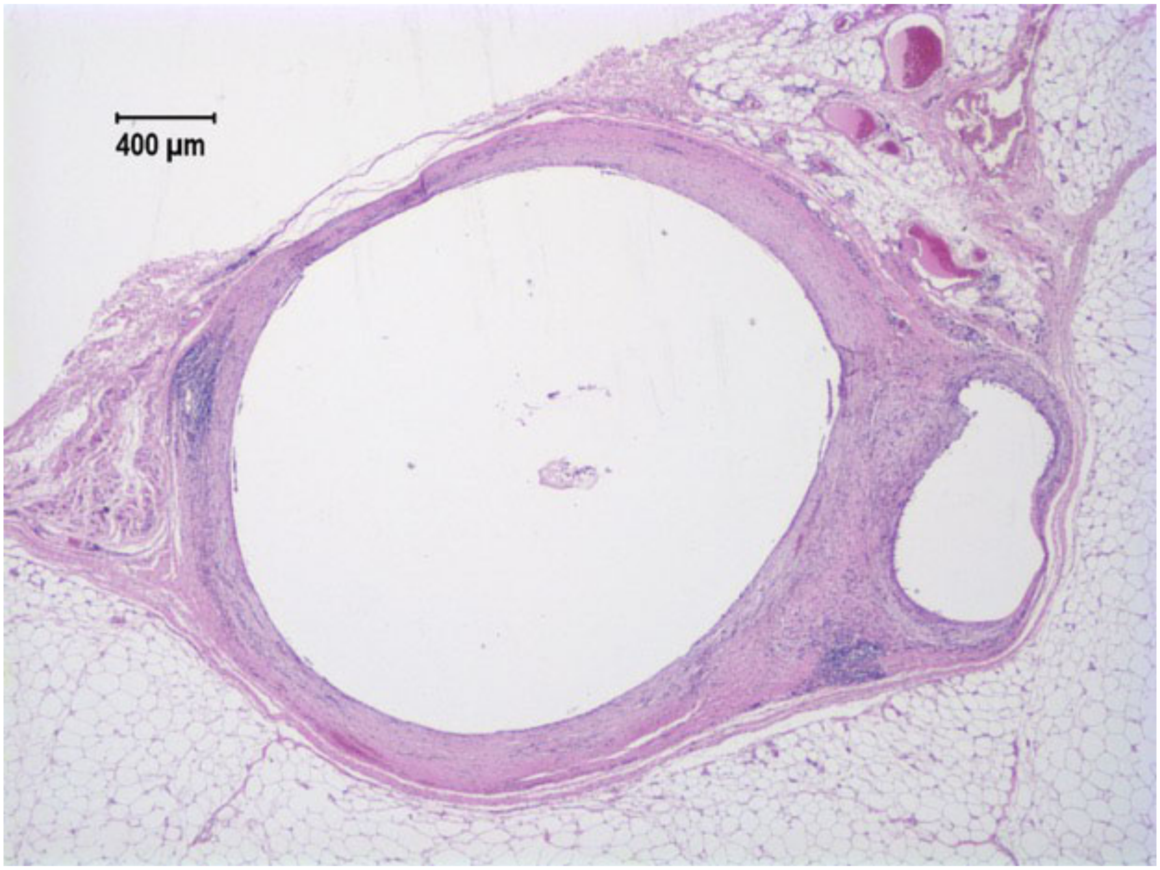

Figure S10. NZW Rabbit M29048. In vitro release rate 0.13 mg/day. Sections from the left implant show moderate chronic inflammation. There are NSL in kidney, liver, spleen, lung, vagina or rectum.

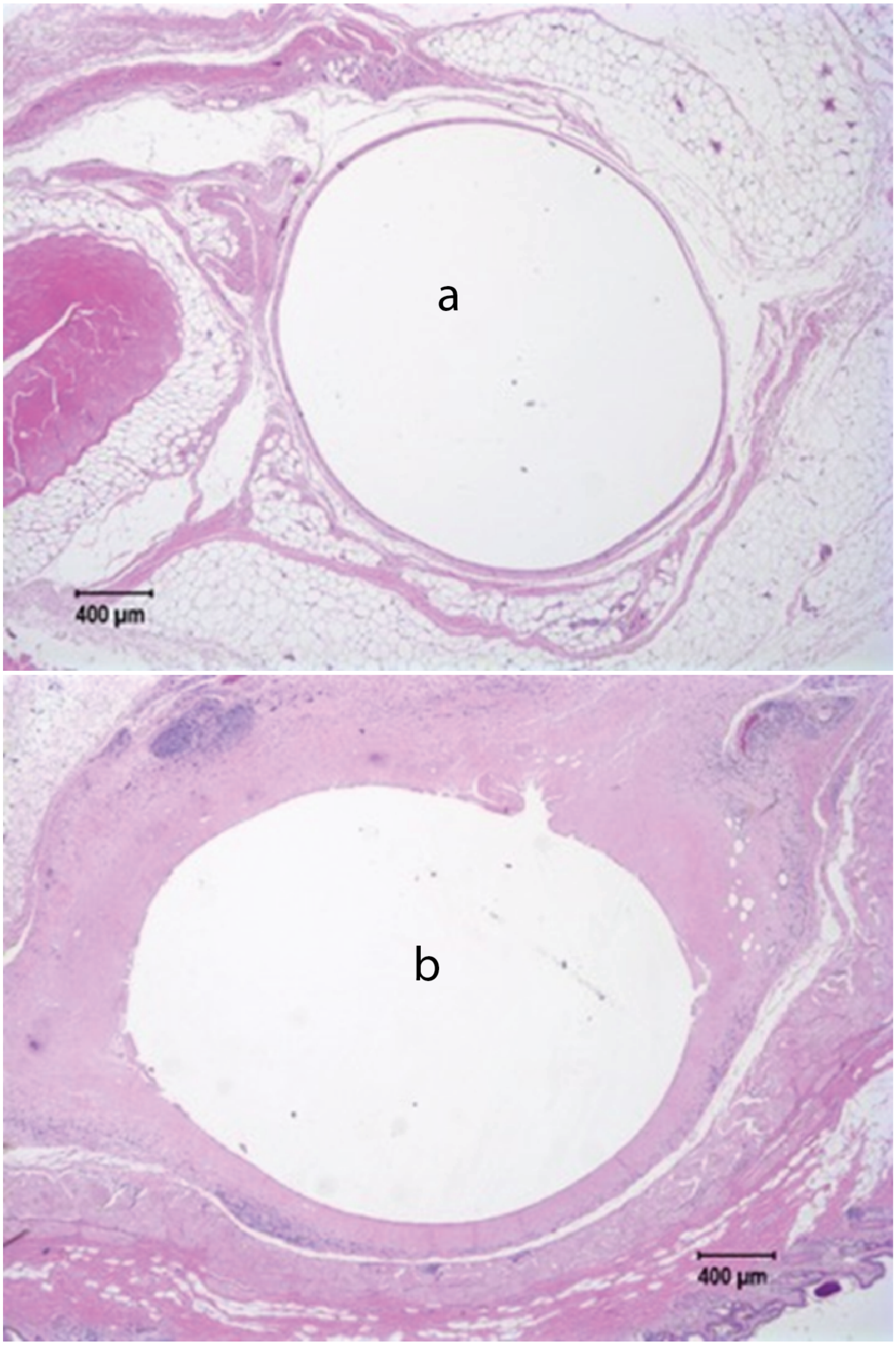

Figure S11. NZW Rabbit M29049**.** In vitro release rate 0.13 mg/day. The left implant (a) shows minimal inflammation, but the right (b) has chronic granulomatous inflammation and necrosis. There is also mild to moderate multifocal periportal lymphocytic hepatitis of unknown etiology. NSL in kidney, spleen, lung, vagina or rectum.

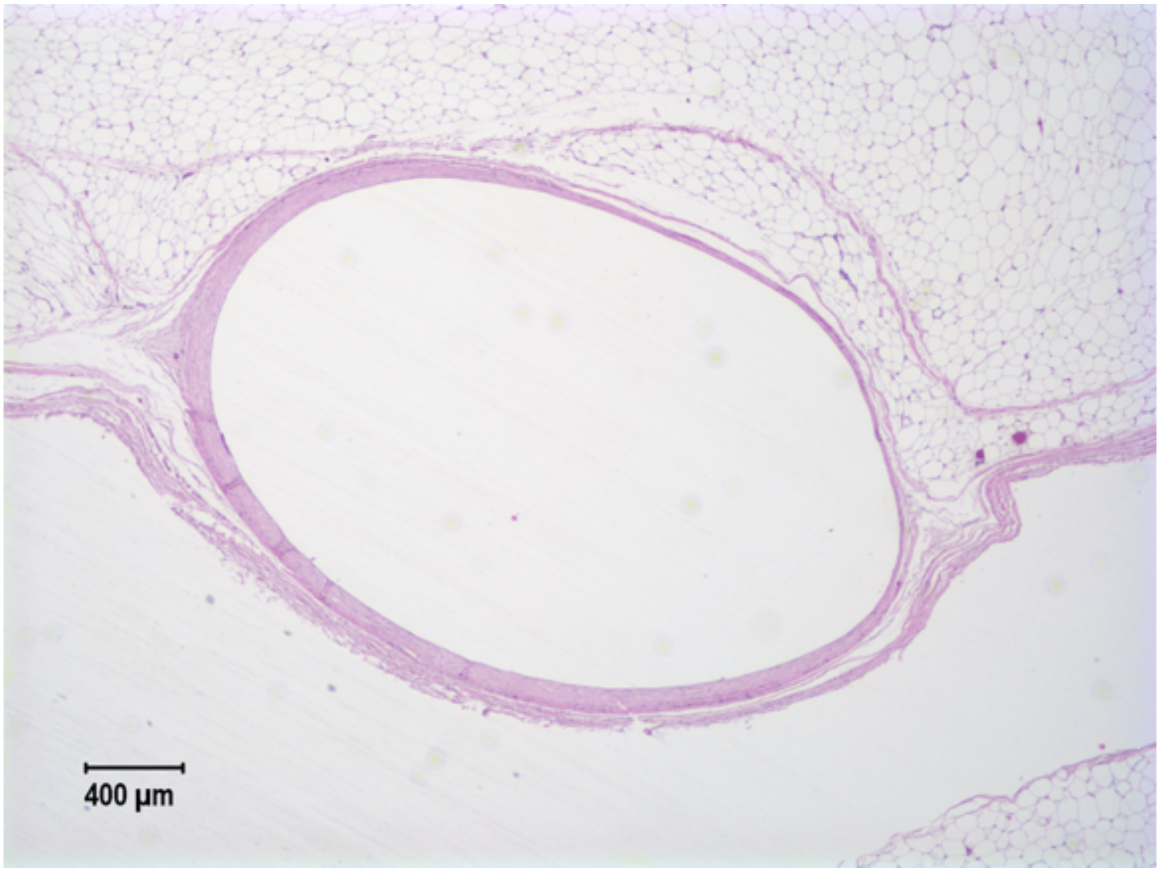

Figure S12. NZW Rabbit M29050. In vitro release rate 0.13 mg/day. There is minimal inflammation associated with the implant. There is mild lymphocytic periportal hepatitis of unknown etiology. NSL are observed in the kidney, spleen, lung, vagina or rectum.

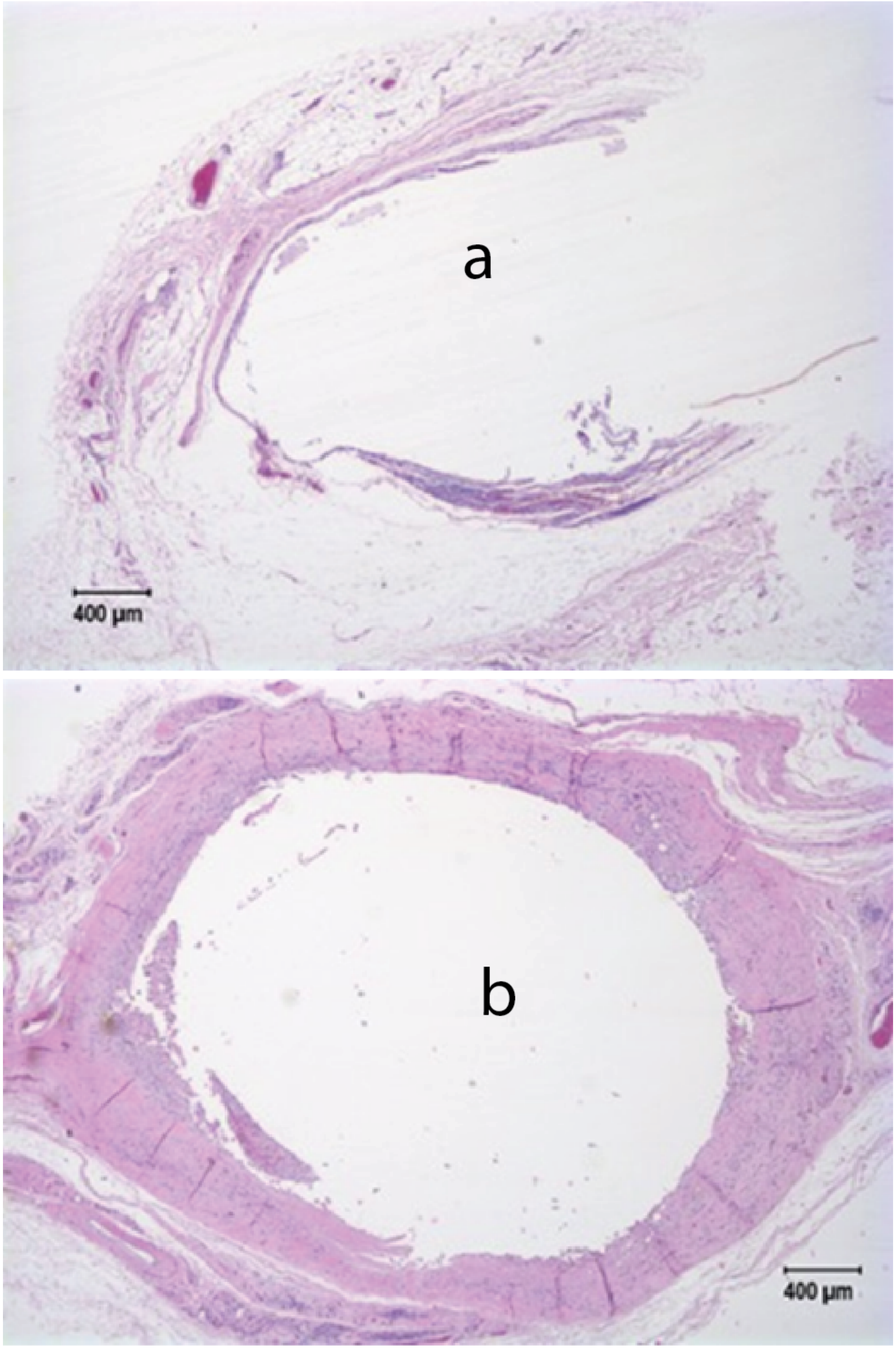

Figure S13. NZW rabbit M29051. In vitro release rate 0.13 mg/day. The right implant (b) has moderate to marked chronic inflammation around the implant, but the left (a) has only mild

mononuclear cell infiltrations. NSL are observed in the liver, kidney, spleen, lung, vagina or rectum.

Group 2: *In vitro* release rate 0.26 mg/day

Table S6. Histological characteristic scores from rabbits M29052 – M29056. Each rabbit received two active 0.8 cm implants.

	**Response**				
**Animal**	**M29052**	**M29053**	**M29054**	**M29055**	**M29056**
Implant	Right	Left	Left	Left	Right
Polymorphonuclear cells	4	3	2	2	4
Lymphocytes	4	4	4	2	4
Plasma cells	4	4	4	2	4
Macrophages	4	4	4	2	4
Giant cells	0	0	0	0	1
Necrosis	3	3	3	1	3
Capsule thickness	3	4	3	3	3
Tissue infiltrate	0	3	0	2	3
Other					
**Overall total**	**22**	**25**	**20**	**14**	**26**

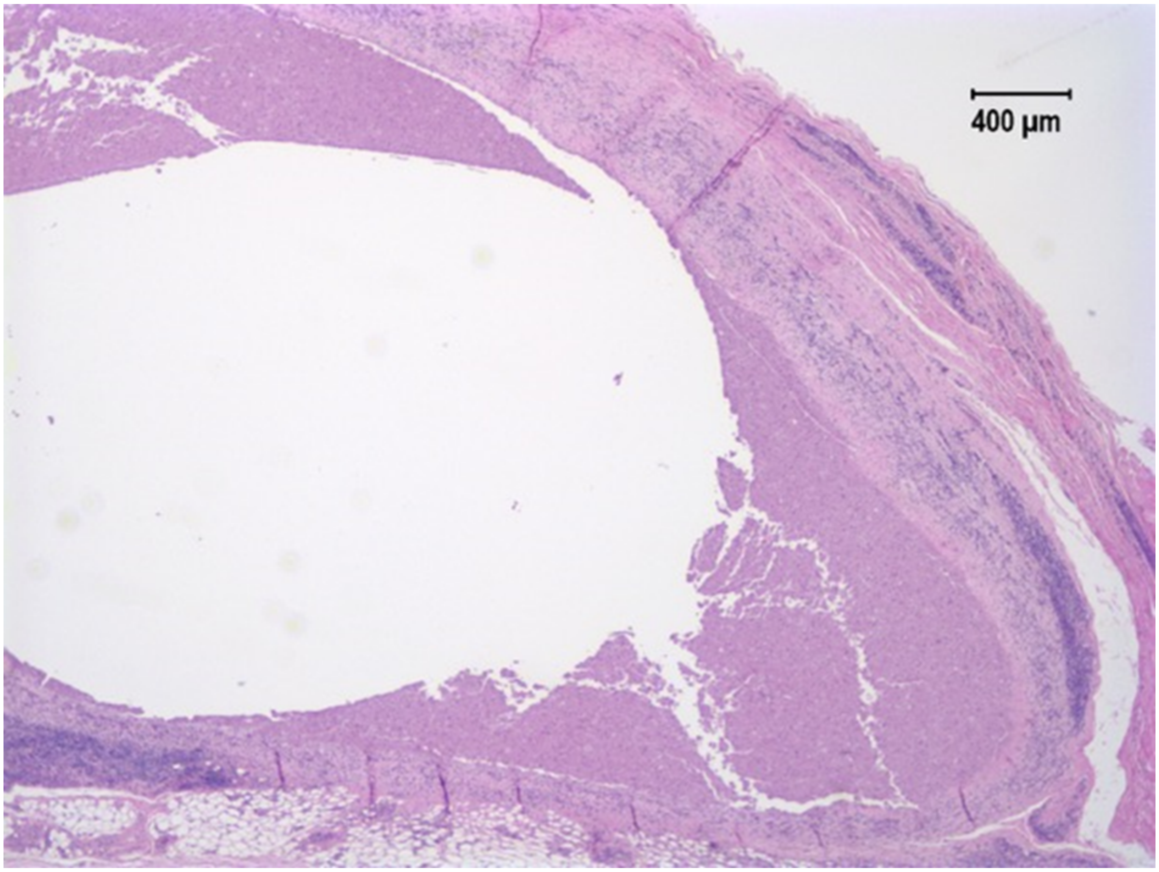

Figure S14. NZW Rabbit M29052. *In vitro* release rate 0.26 mg/day. All sections of the right implant have marked inflammation around the implant and necrotic cells in the center. NSL are observed in the liver, kidney, spleen, lung, vagina or rectum.

123

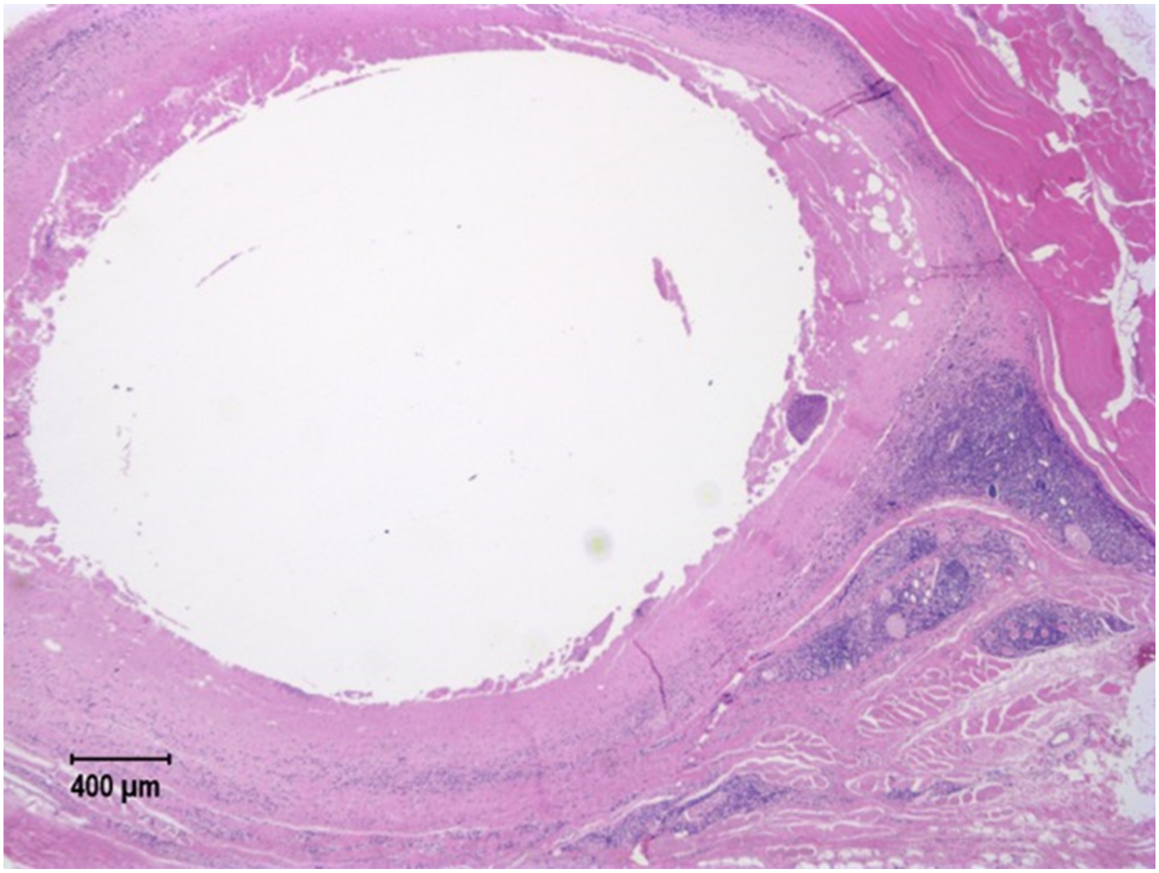

Figure S15. NZW Rabbit M29053**.** *In vitro* release rate 0.26 mg/day. All sections of the left implant have marked inflammation associated with the implant and necrotic tissue and cells in the center. NSL are observed in the liver, kidney, spleen, lung, vagina or rectum.

124

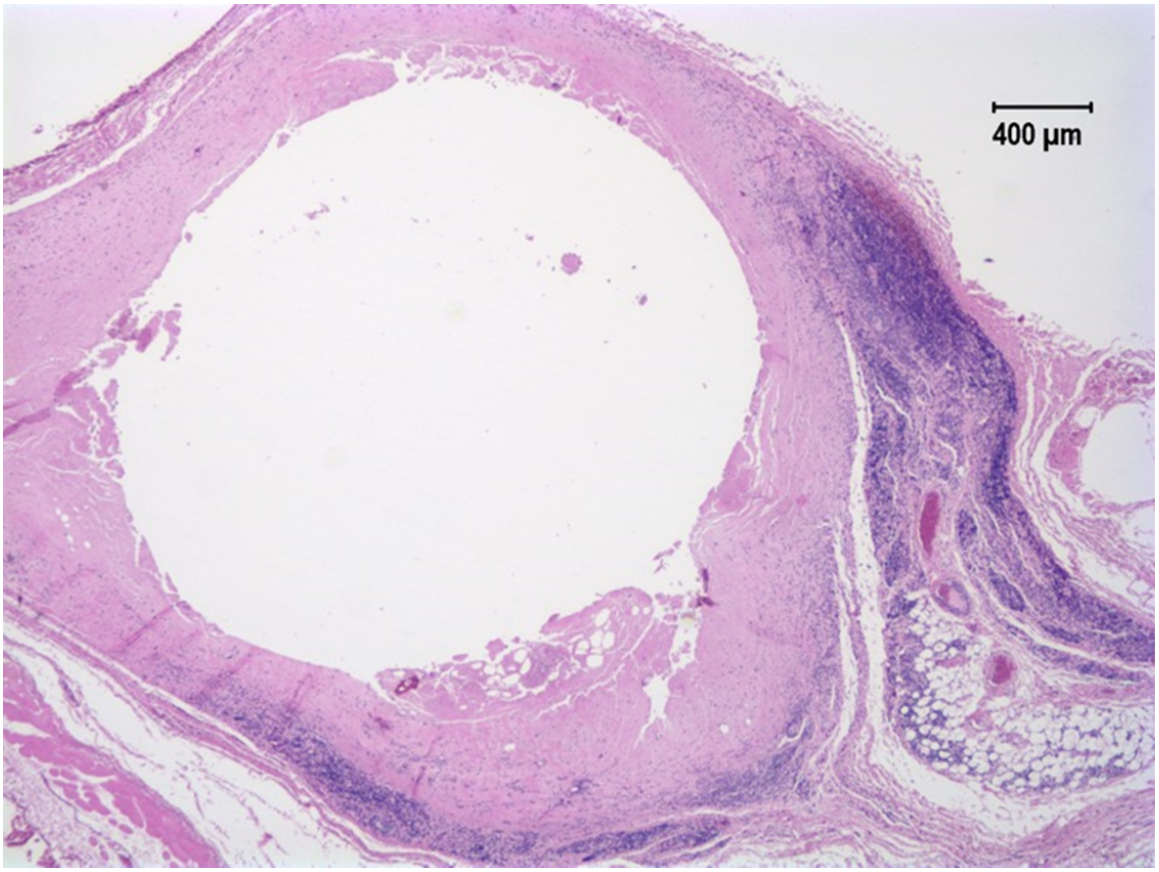

Figure S16. NZW Rabbit M29054. *In vitro* release rate 0.26 mg/day. All sections of the right implant have moderate to marked inflammation associated with the implant and necrotic tissue and cells in the center. NSL are observed in the liver, kidney, spleen, lung, vagina or rectum.

125

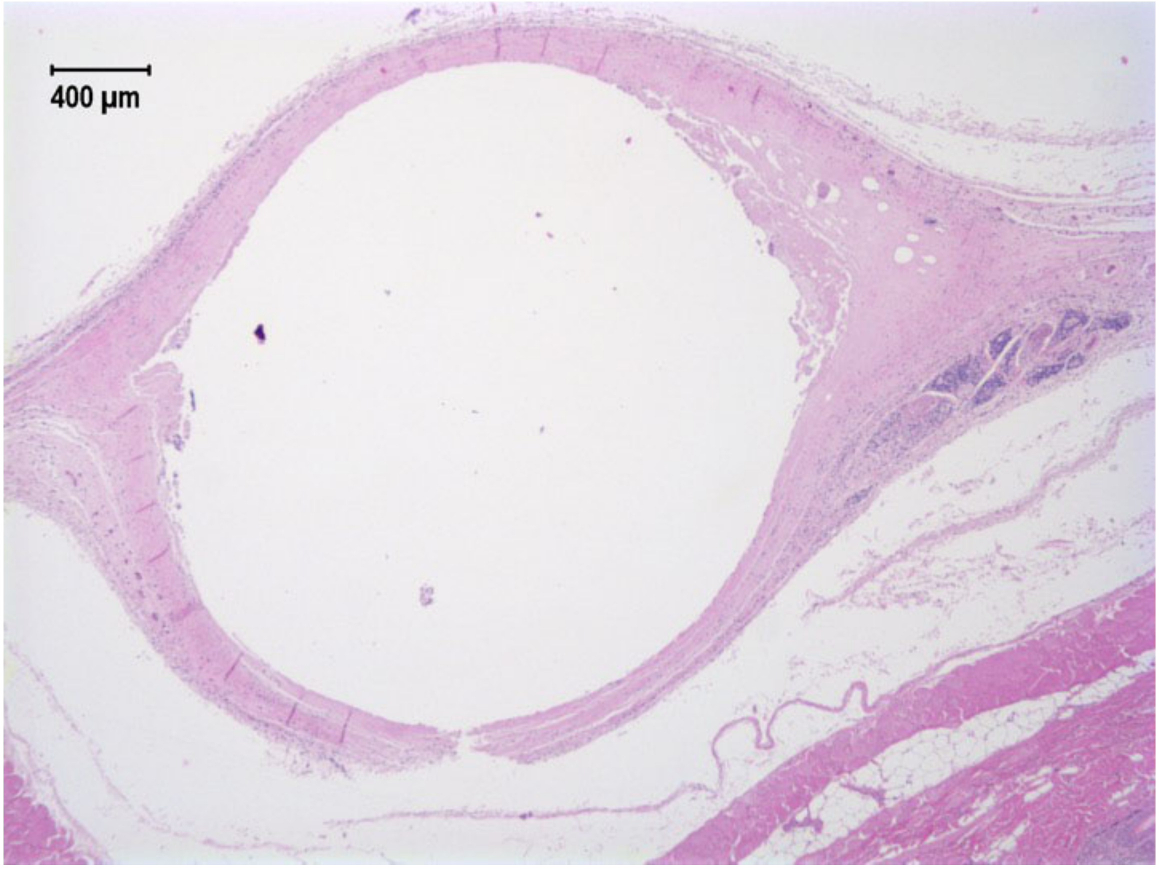

Figure S17. NZW Rabbit M29055. *In vitro* release rate 0.26 mg/day. There is moderate inflammation associated with the implant. There is mild lymphocytic periportal hepatitis. NSL are observed in the kidney, spleen, lung, vagina or rectum.

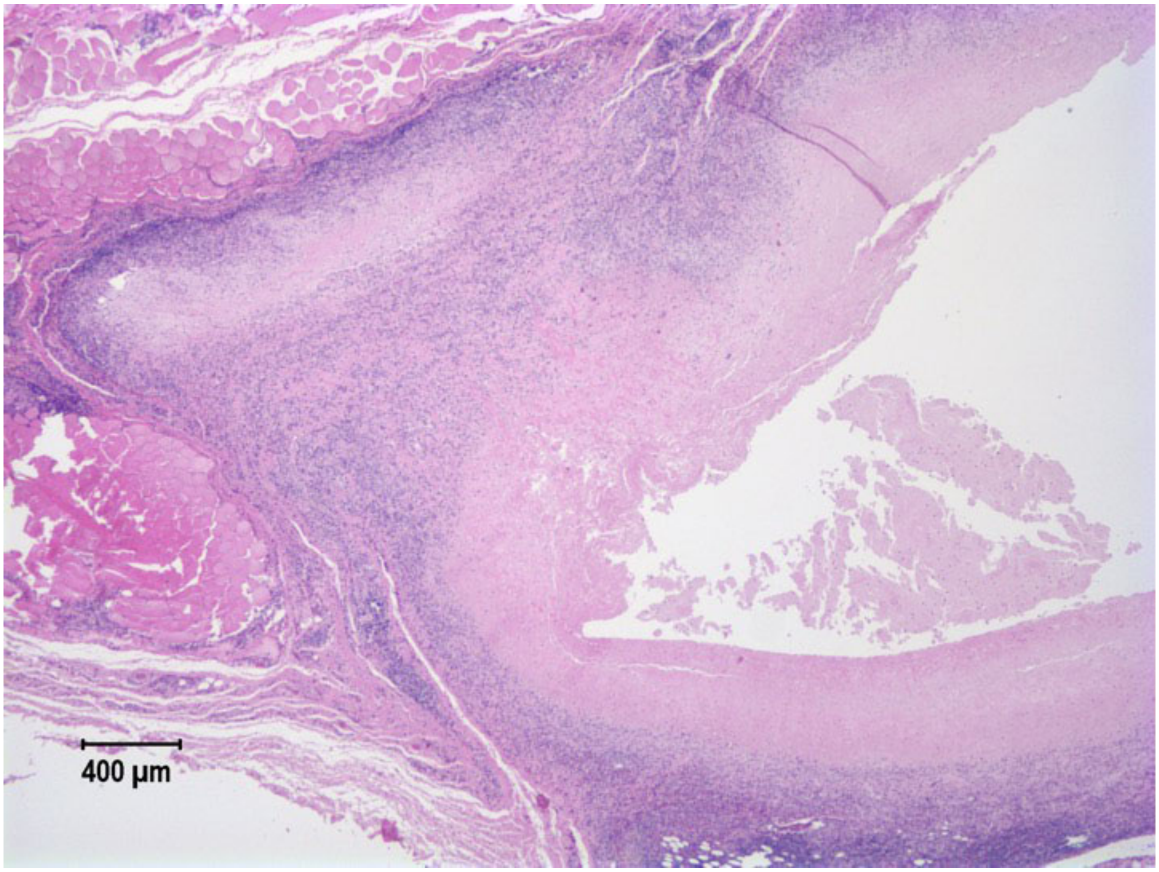

Figure S18. NZW Rabbit M29056. *In vitro* release rate 0.26 mg/day. There is marked inflammation and moderate necrosis associated with the implant. There is mild lymphocytic periportal hepatitis. NSL are observed in the kidney, spleen, lung, vagina or rectum.

Group 3: *In vitro* release rate 0.48 mg/day

Table S7. Histological characteristic scores for animals M29057 – M29061. Each animal received two active 1.6 cm implants.

	**Response**				
Animal	**M29057**	**M29058**	**M29059**	**M29060**	**M29061**
Implant	Right	Left	Left	Left	Right
Polymorphonuclear cells	2	4	4	4	4
Lymphocytes	3	4	4	4	4
Plasma cells	3	4	4	4	4
Macrophages	3	4	4	4	4
Giant cells	0	0	0	0	0
Necrosis	3	3	3	4	3
Capsule thickness	0	3	3	3	3
Tissue infiltrate	0	3	3	3	3
Other					
**Overall total**	**14**	**25**	**25 (marked inflammation)**	**26**	**25 (marked inflammation)**

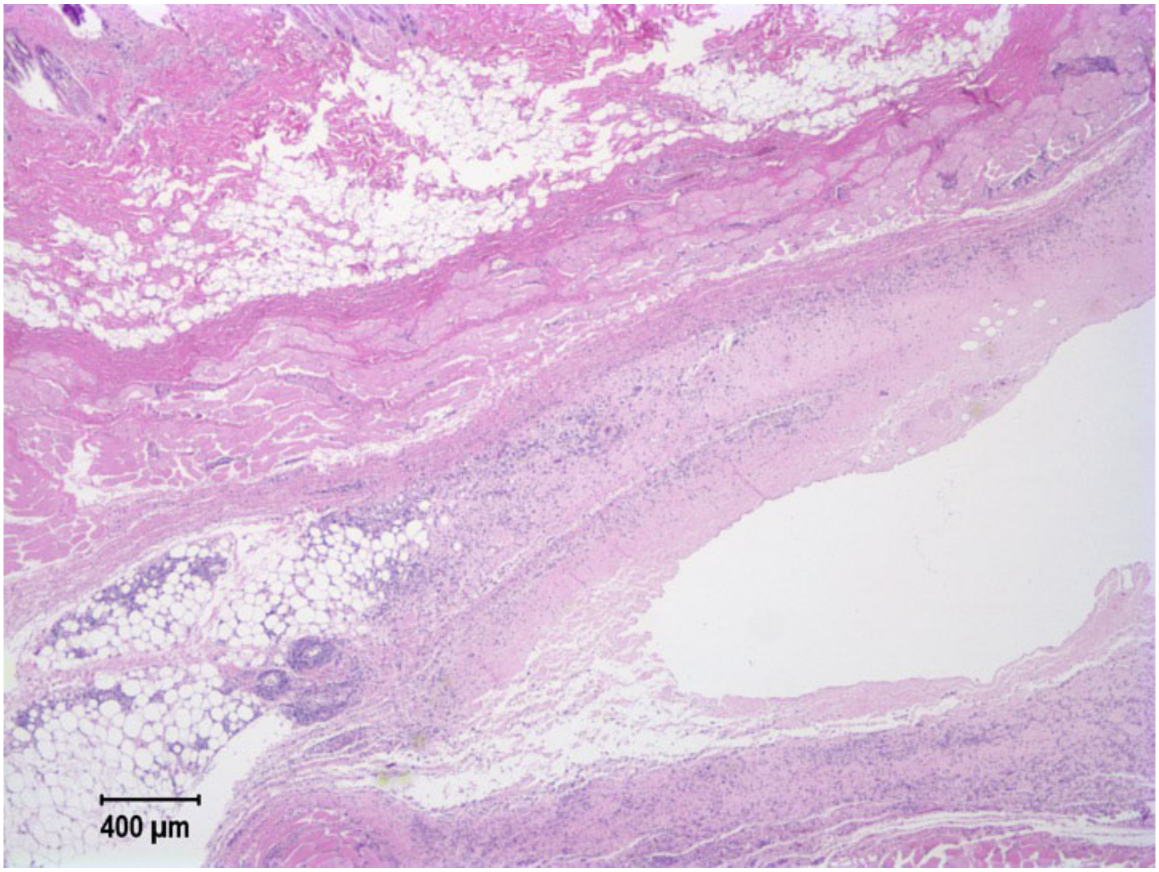

Figure S19. NZW Rabbit M29057. *In vitro* release rate 0.48 mg/day. There is moderate to marked inflammation and moderate necrosis associated with the implant. NSL are observed in the liver, kidney, spleen, lung, vagina or rectum

134

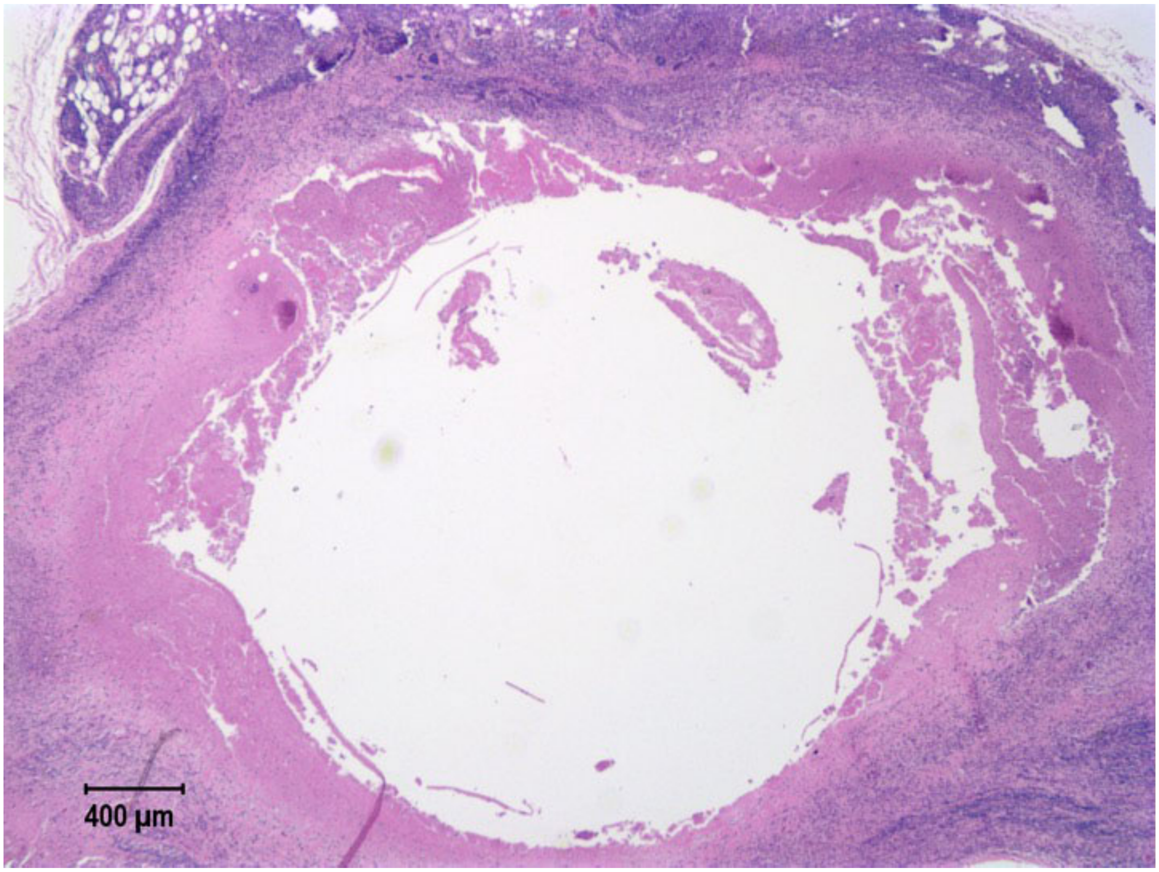

Figure S20. NZW Rabbit M29058. *In vitro* release rate 0.48 mg/day. All sections from the left implant show marked inflammation and moderate necrosis associated with the implant. NSL are observed in the liver, kidney, spleen, lung, vagina or rectum

135

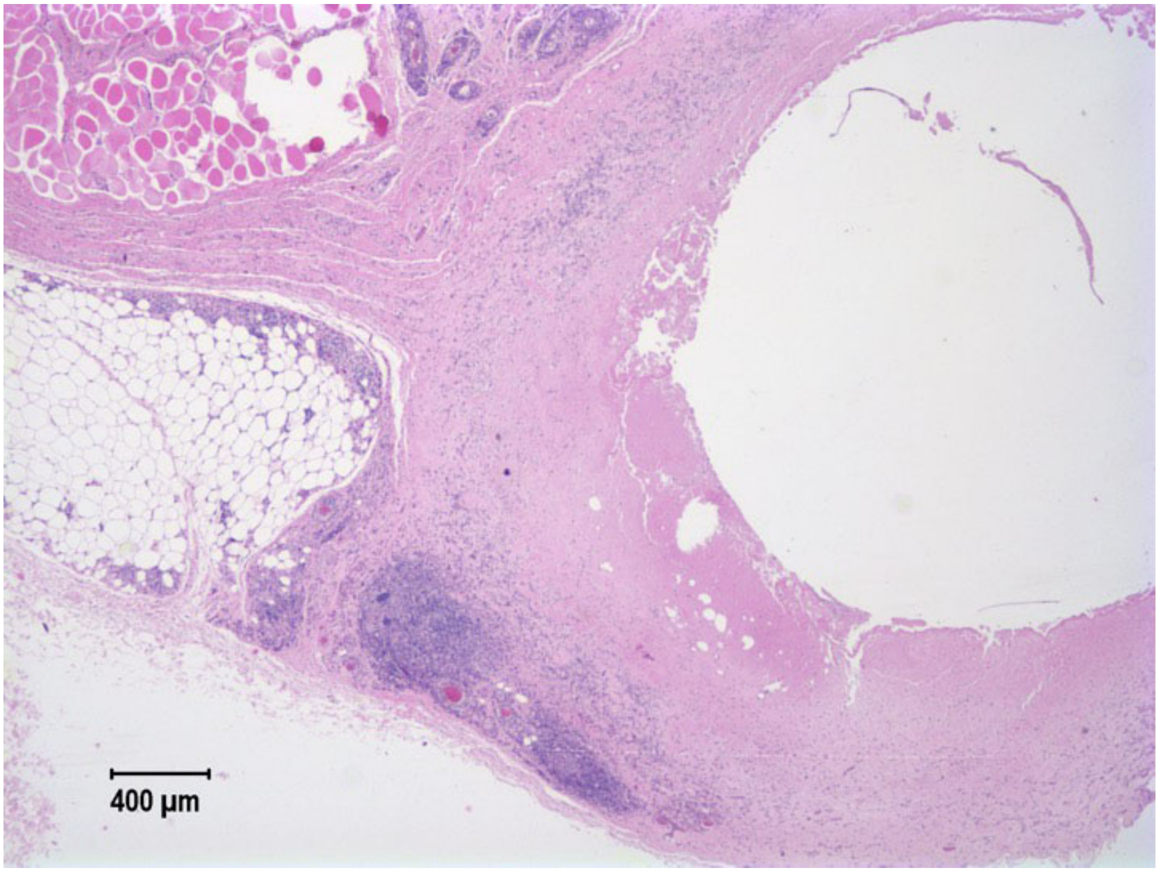

Figure S21. NZW Rabbit M29059. *In vitro* release rate 0.48 mg/day. All sections from the right implant show marked inflammation and moderate necrosis associated with the implant. NSL are observed in the liver, kidney, spleen, lung, vagina or rectum

136

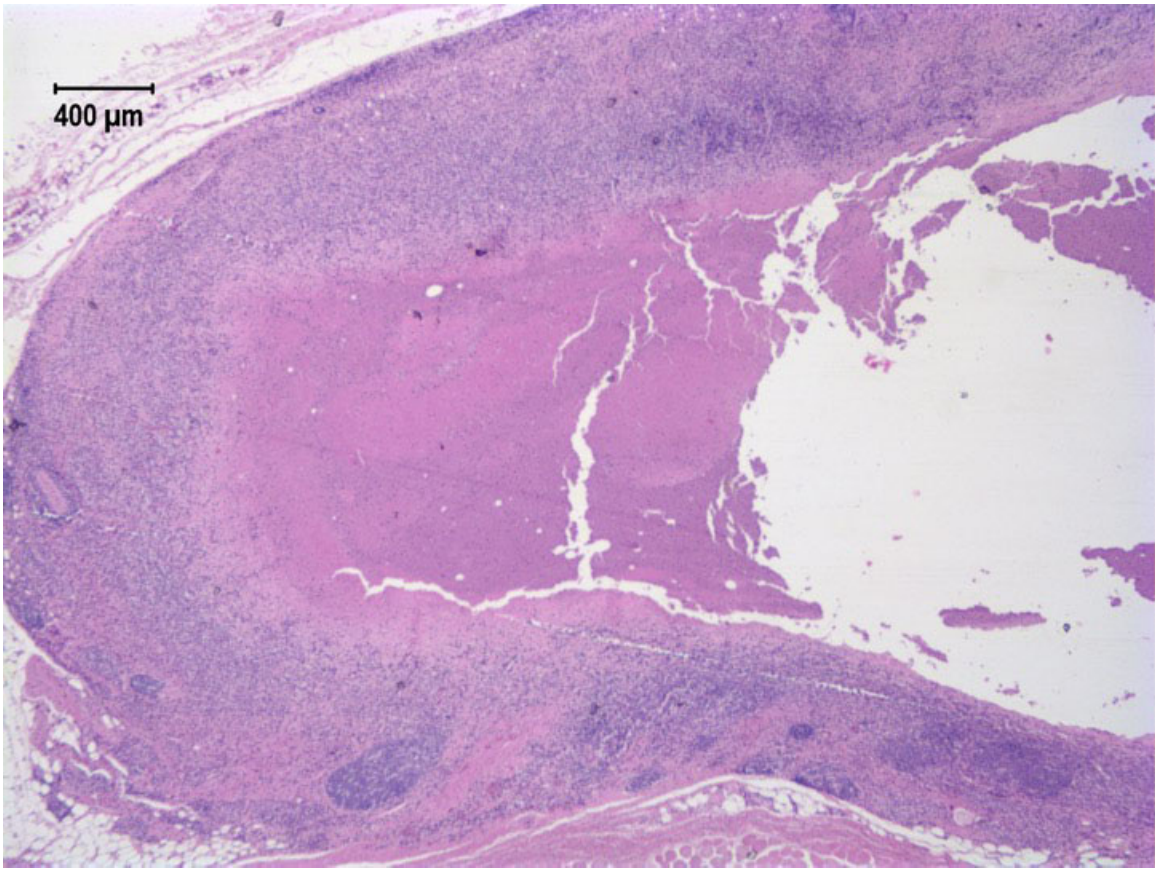

Figure S22. NZW Rabbit M29060. *In vitro* release rate 0.48 mg/day. All sections from the left implant show marked inflammation and moderate necrosis associated with the implant. NSL are observed in the liver, kidney, spleen, lung, vagina or rectum

137

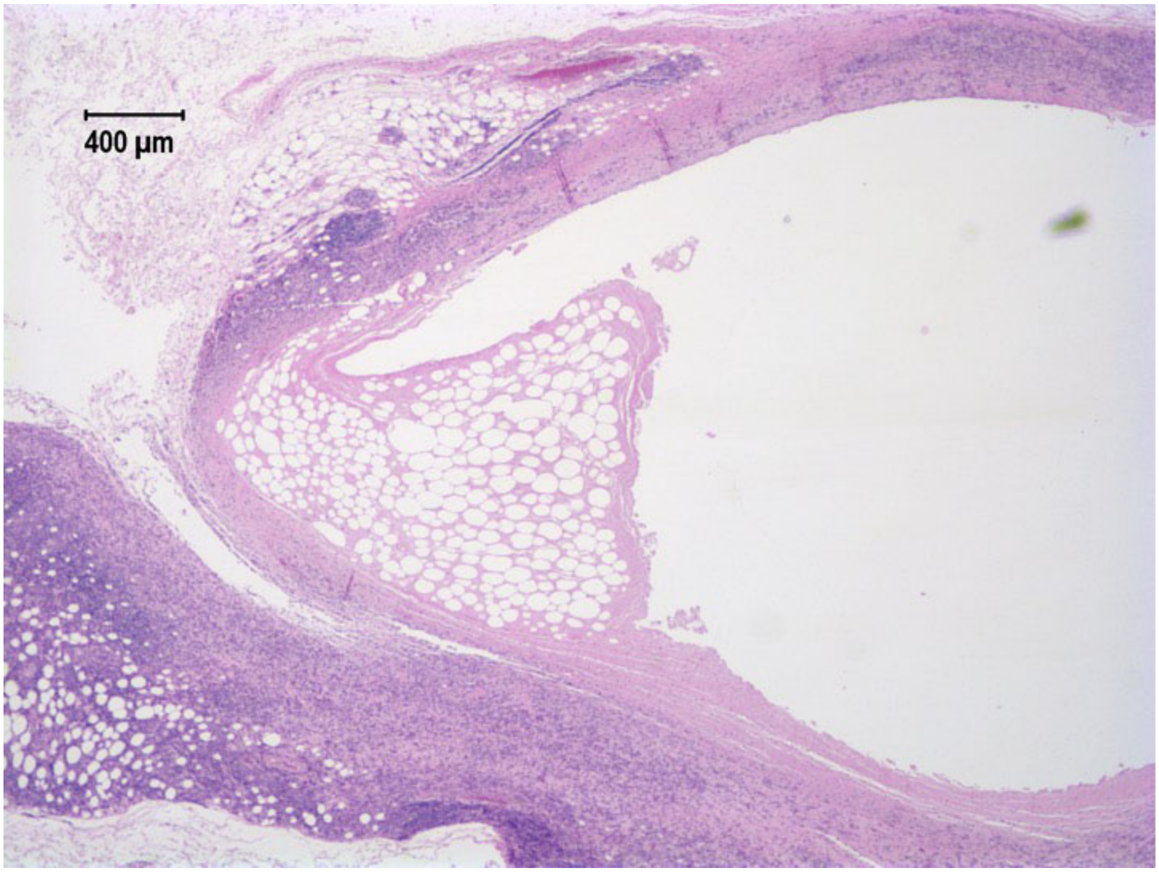

Figure S23. NZW Rabbit M29061. *In vitro* release rate 0.48 mg/day. All sections from the left implant show marked inflammation and moderate necrosis associated with the implant. NSL are observed in the liver, kidney, spleen, lung, vagina or rectum

Group 4: *In vitro* release rate of 0.72 mg/day

Table S8. Histological characteristic scores for animals M29062 – M29064. Each animal received three active 1.6 cm implants.

	**Response**		
Animal	M29062	M29063	M29064
Implant	Right	Right	Left
Polymorphonuclear cells	2	4	3
Lymphocytes	3	4	4
Plasma cells	2	4	4
Macrophages	2	4	4
Giant cells	0	0	0
Necrosis	1	3	3
Capsule thickness	2	3	3
Tissue infiltrate	3	3	3
**Overall total**	**15**	**25**	**24**

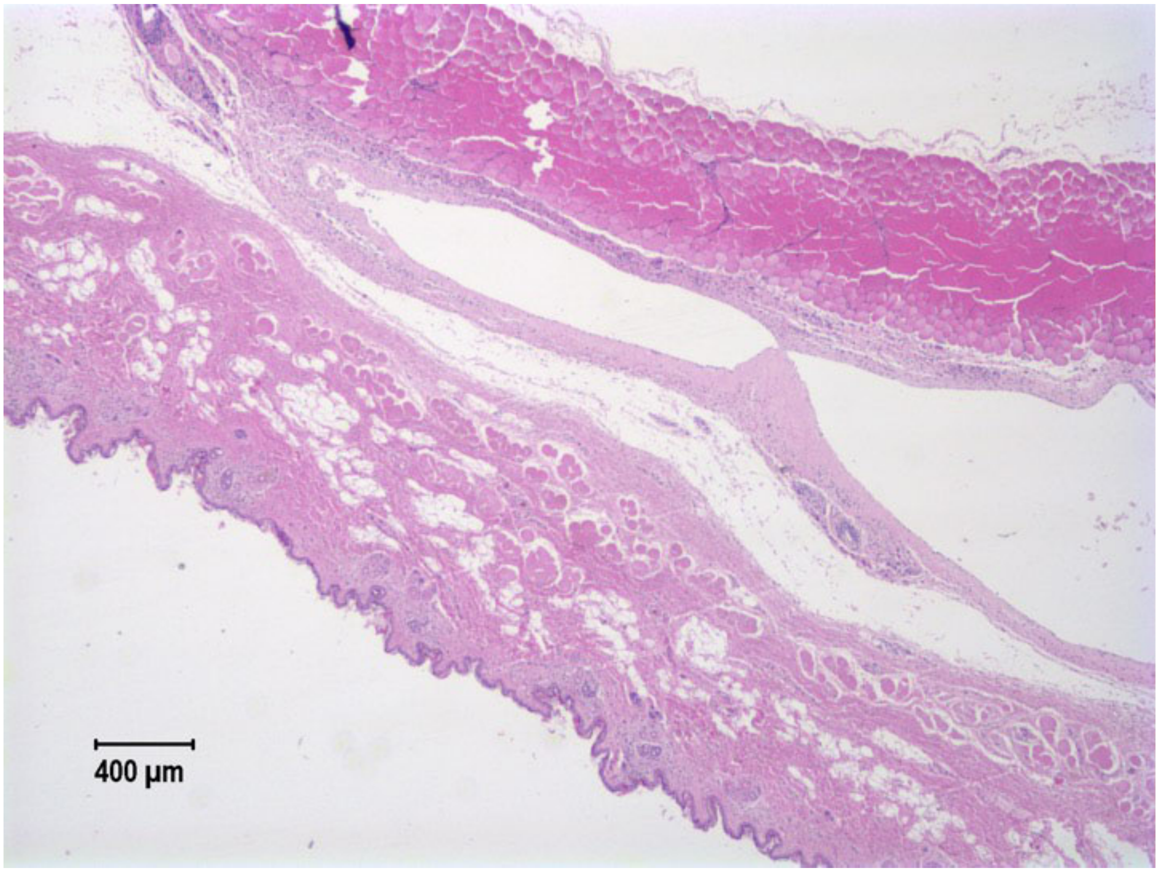

Figure S24. NZW Rabbit M29062. *In vitro* release rate 0.72 mg/day. All sections from the left implant show marked inflammation and moderate necrosis associated with the implant. NSL are observed in the liver, kidney, spleen, lung, vagina or rectum

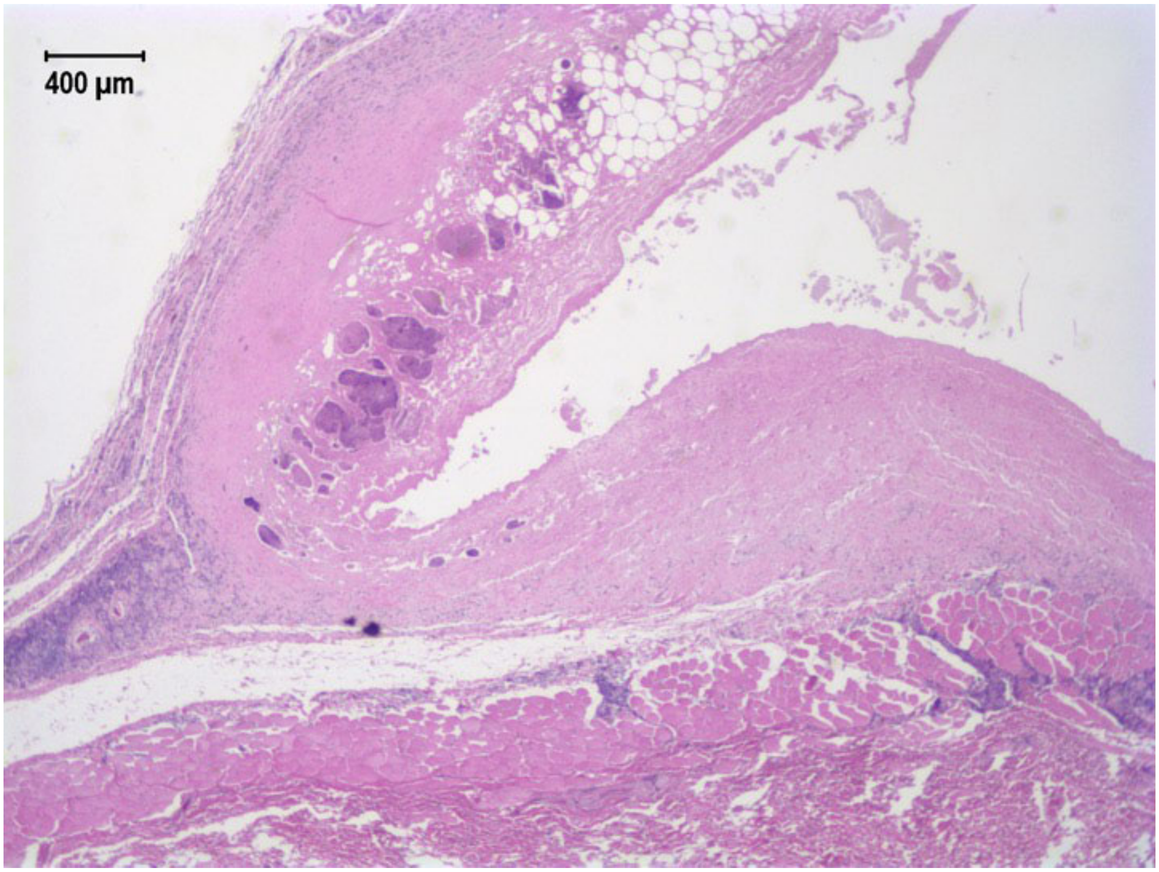

Figure S25. NZW Rabbit M29063. *In vitro* release rate 0.72 mg/day. All sections from the right implant show marked inflammation and moderate necrosis associated with the implant. NSL are observed in the liver, kidney, spleen, lung, vagina or rectum

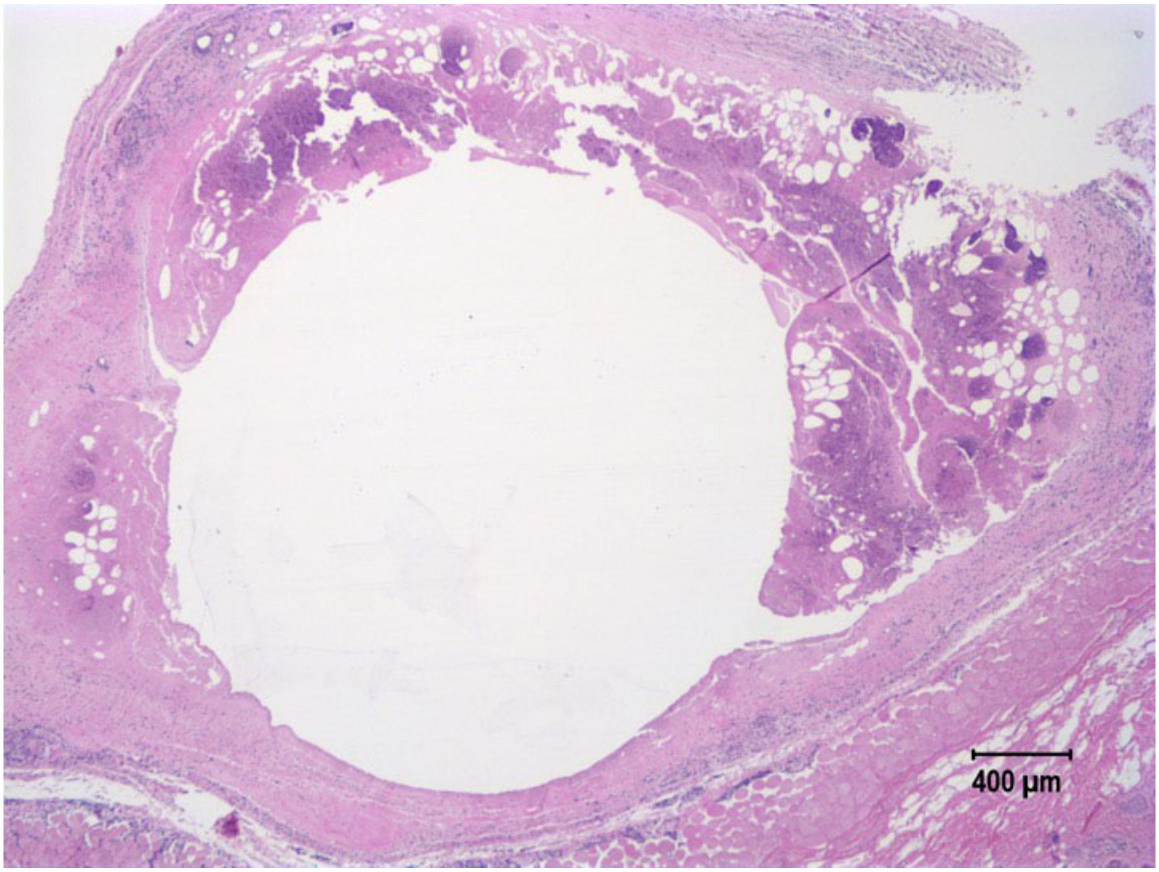

Figure S26. NZW Rabbit M29064. *In vitro* release rate 0.72 mg/day. All sections from the left implant show marked inflammation and moderate necrosis associated with the implant. NSL are observed in the liver, kidney, spleen, lung, vagina or rectum.

Supplemental 5. 4 week and 12-week histology report of TAF generation B implants in rhesus macaques.

Note: Each rhesus macaque received two 2 cm implants: one placebo (right), and one active (left).

Four-week histology reports:

Table S9. Histological characteristic scores for rhesus macaque DP15. Implant placed 2/22/2018; euthanized 3/22/2018.

**Animal# DP15 Necropsy# 18A100**	**Response**	
Implant	Left (Active) (1-4)	Right (Placebo) (1-4)
Polymorphonuclear cells	3	1
Lymphocytes	3	1
Plasma cells	3	1
Macrophages	3	2
Giant cells	2*	0
Necrosis	2	0
Capsule thickness	3	2
Tissue infiltrate	3	1
Other		
**Overall total**	**22**	**8**

*NB: There are significant multifocal areas of granulomatous inflammation associated with suture material which often coalesces with the implant margins, and most multinucleated giant cells (MNGC) appear to be associated with these rather than the implant.

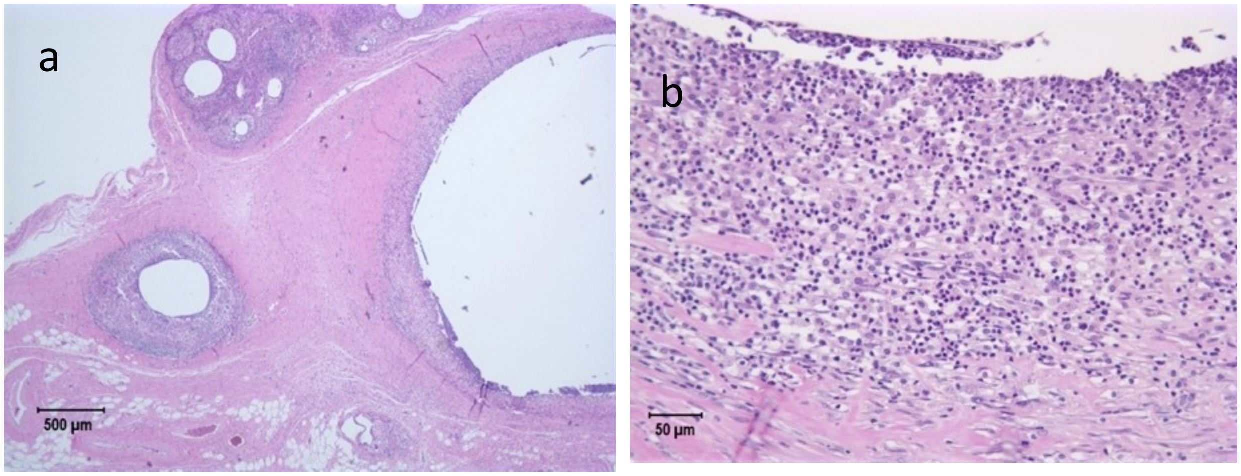

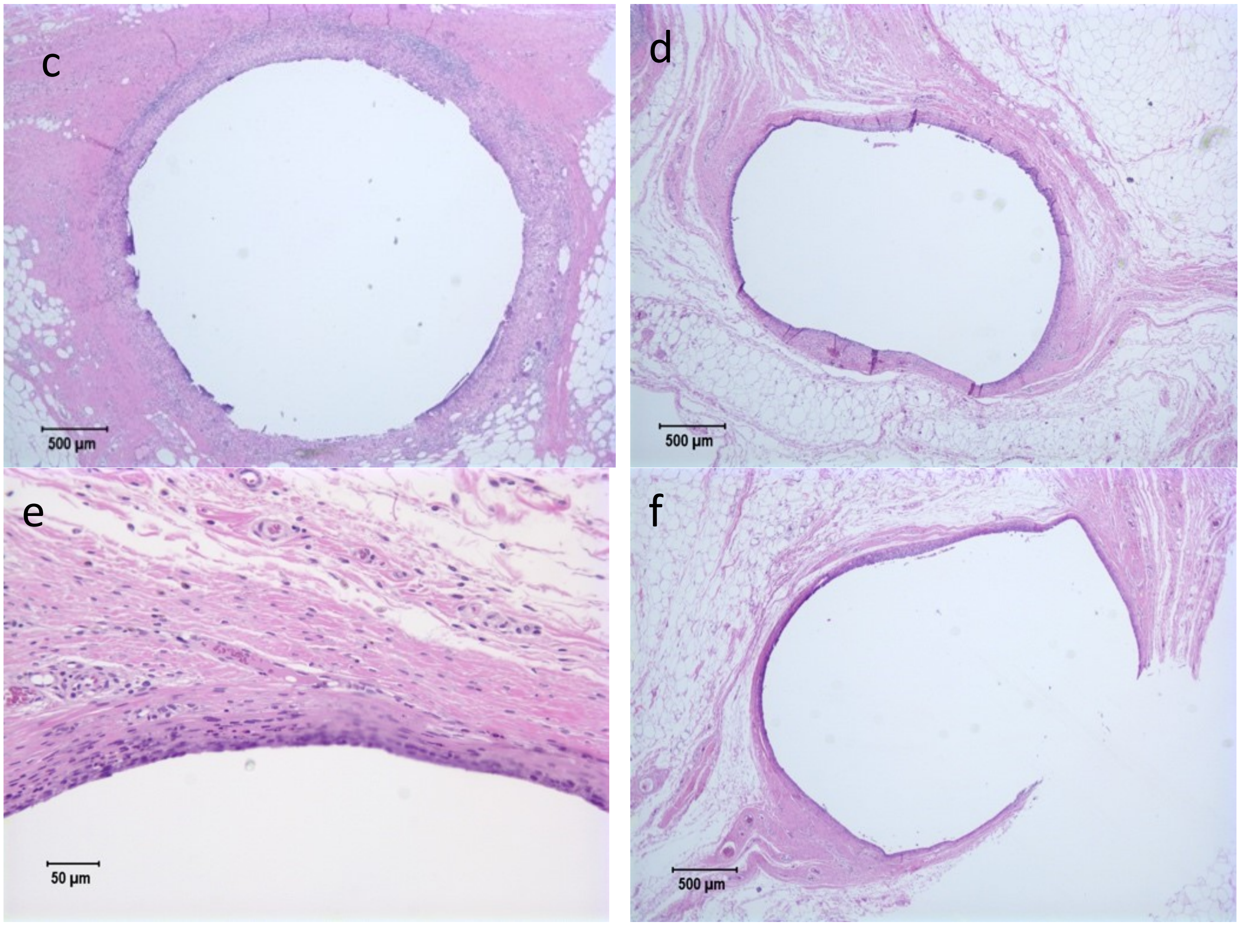

Figure S27. Rhesus macaque DP15. *In vitro* release rate 0.13 mg/day. The left (active) implant (a-c) shows a moderately thick band of heterophils, macrophages, lymphocytes, and plasma cells surrounding implant interspersed with moderate numbers of multinucleated giant cells in peripheral sections. Fibrous tissue and inflammation extend into adjacent connective tissue. The right (placebo) implant (d-f) clearly has less inflammation, yet adjacent connective tissues of both implants have some fibrosis and minimal inflammation. *NB: There are significant multifocal areas of granulomatous inflammation associated with suture material which often coalesces with the implant margins, and most MNGC appear to be associated with these rather than the implant.

Table S10. Histological characteristic scores for rhesus macaque GH84. Implant placed 2/22/2018; euthanized 3/22/2018.

**Animal# GH84 Necropsy# 18A101**	**Response**	
Implant	Left (Active) 1-4	Right (Placebo) 1-4
Polymorphonuclear cells	3	0
Lymphocytes	3	1
Plasma cells	2	1
Macrophages	2	1
Giant cells	1*	0

Necrosis	1	0
Capsule thickness	3	1
Tissue infiltrate	2	1
Other		
**Overall total**	**17**	**5**

***NB: There are significant multifocal areas of granulomatous inflammation associated with suture material which often coalesces with the implant margins, and most MNGC appear to be associated with these rather than the implant.**

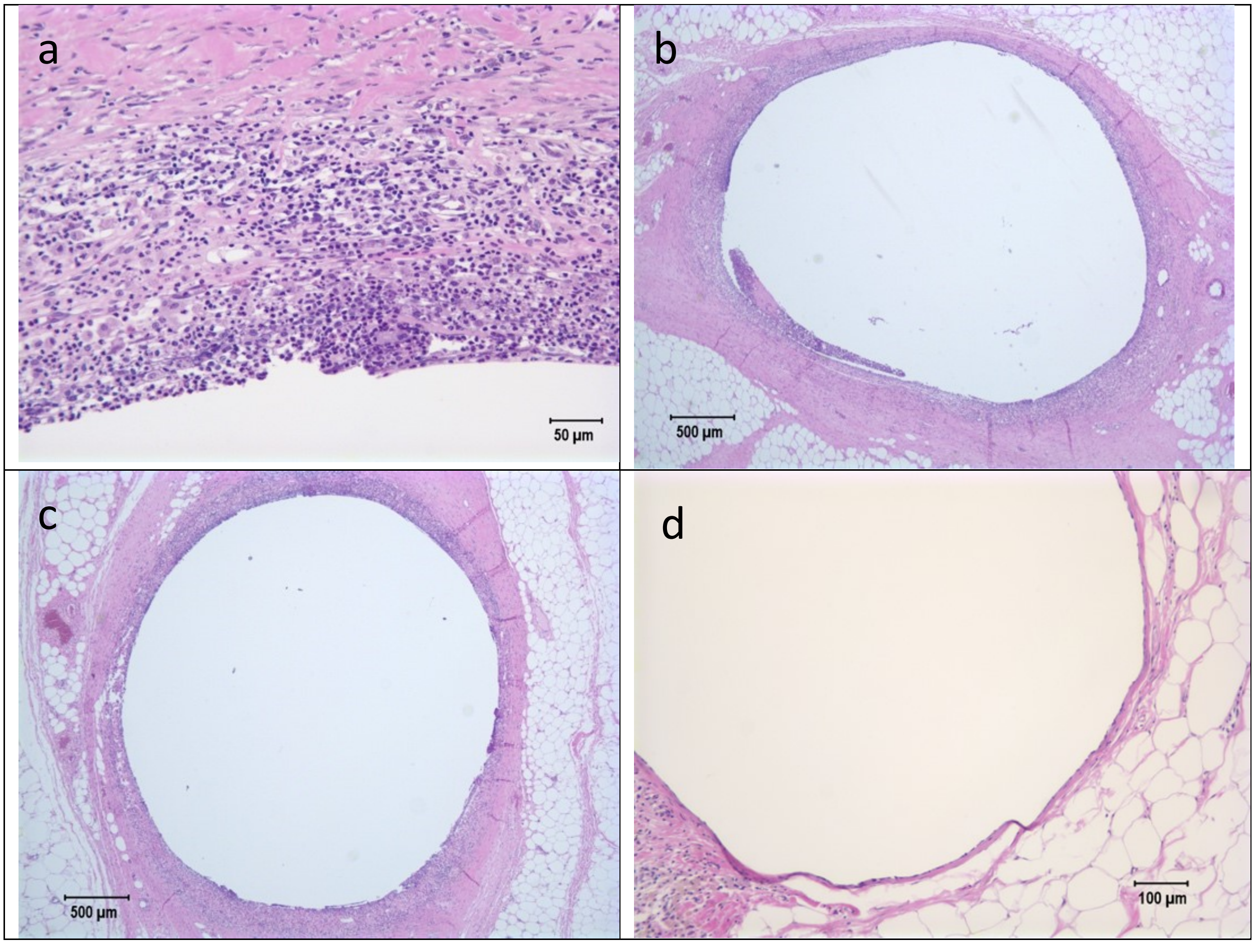

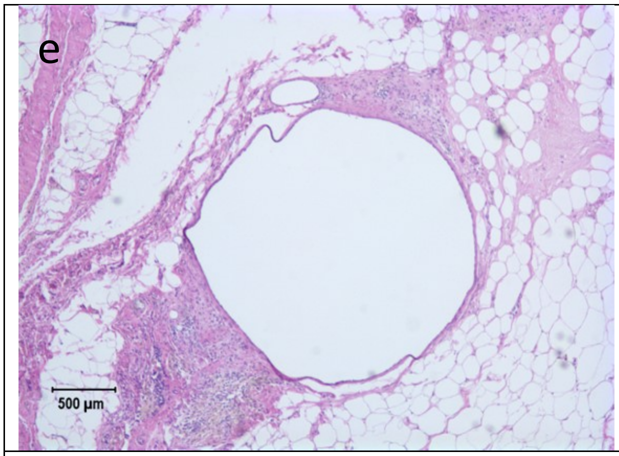

Figure S28. Rhesus macaque GH84. *In vitro* release rate 0.13 mg/day. The left (active) implant (a-c) shows a moderately thick band of heterophils, macrophages, lymphocytes, and plasma cells surrounding implant. Fibrous tissue and inflammation extend into adjacent connective tissue. Right (placebo) implant (d-e)has less inflammation, yet adjacent connective tissues of both implants have some fibrosis and minimal inflammation.

12-week histology reports:

Table S11. Histological characteristic scores for rhesus macaque FC48. An implant placed 2/22/2018; euthanized 5/17/2018.

**Animal# FC48<br>Necropsy# 18A219**	**Response**	
Implant	Left (Active) (1-4)	Right (Placebo) (1-4)
Polymorphonuclear cells	4	2
Lymphocytes	4	3
Plasma cells	4	2
Macrophages	3	3
Giant cells	0	0
Necrosis	2	1
Capsule thickness	3	2
Tissue infiltrate	4	3
Other		
**Overall total**	**24**	**16**

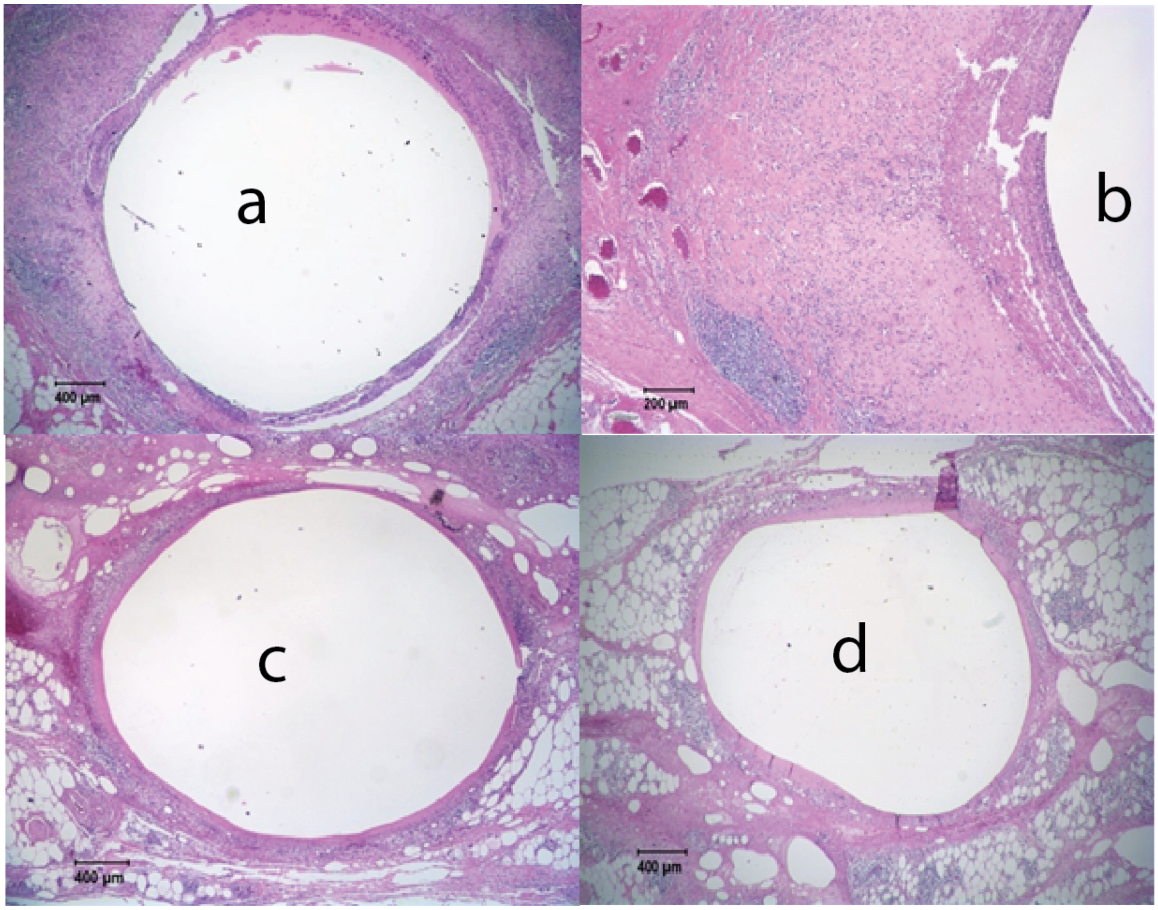

Figure S29. Rhesus macaque FC48. *In vitro* release rate 0.13 mg/day. Right implant (placebo) (c,d) has a thin capsule, and mild pericapsular infiltrates of lymphocytes and plasma cells, but deeper CT has multifocal aggregates of lymphocytes, plasma cells, edema, and hemorrhage. Left (active) implant (a,b) has thick capsule filled with proteinaceous fluid, heterophils, plasma cells, macrophages. Extensive fibrosis and lymphoplasmacytic inflammation extend into adjacent tissues

Table S12. Histological characteristic scores for rhesus macaque EC74. Implant placed 2/22/2018; euthanized 5/17/2018.

**Animal# EC74 Necropsy# 18A220**	**Response**	
Implant	*Left (Active) (1-4)	Right (Placebo) (1-4)
Polymorphonuclear cells	4	1
Lymphocytes	4	3
Plasma cells	4	2
Macrophages	4	4
Giant cells	4	3
Necrosis	4	1
Capsule thickness	3	3

Tissue infiltrate	4	3
Other		
**Overall total**	**31**	**20**

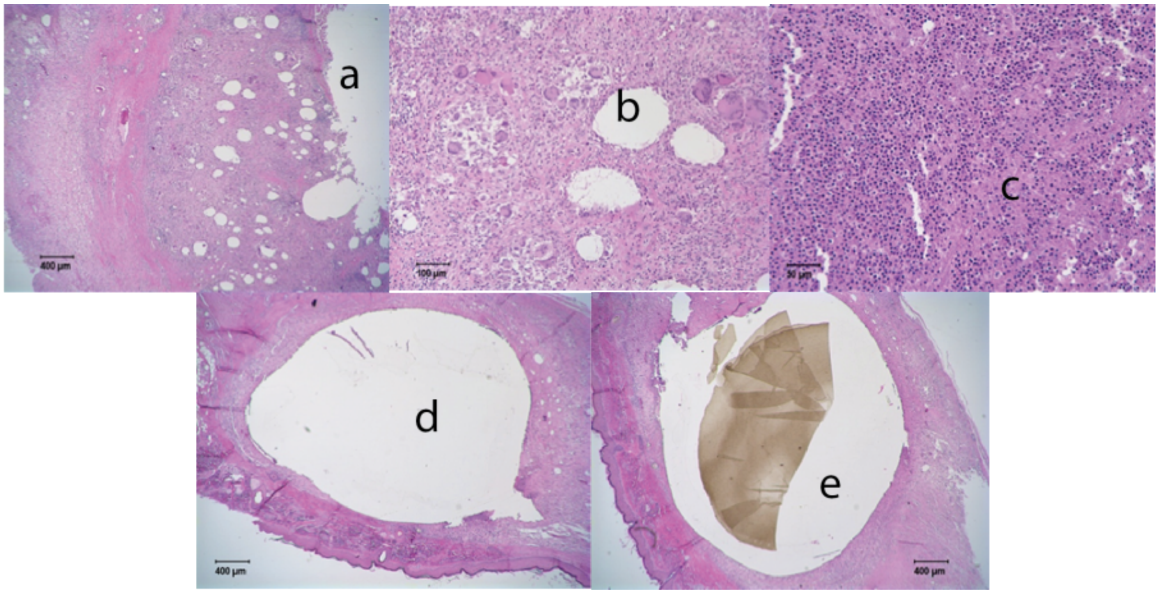

Figure S30. Rhesus macaque EC74. *In vitro* release rate 0.13 mg/day. *The left implant (active) (a, b, c) could not be located, but there was a purulent hemorrhagic abscess with fibrosis in the region and histology shows marked necrotic cellular material surrounded by severe granulomatous inflammation with abundant multinucleated giant cells. The left implant was lost through abscess formation before necropsy. The right implant (placebo) (d, e) has marked accumulations of macrophages and lymphocytes with moderate numbers of giant cells around the implant but minimal heterophils, plasma cells, or necrosis. Panel e shows a remnant of a sectioned piece of the membrane wall the occupies the implant volume after sectioning.

Table S13. Histological characteristic scores for rhesus macaque EE22. Implant placed 2/22/2018; euthanized 5/24/2018.

**Animal# EE22 Necropsy# 18A233**	**Response**	
Implant	Left (Active) (1-4)	Right (Placebo) (1-4)
Polymorphonuclear cells	4	0
Lymphocytes	4	1
Plasma cells	4	0
Macrophages	4	0
Giant cells	0	0
Necrosis	3	0
Capsule thickness	3	1
Tissue infiltrate	4	0
Other		

**Overall total**	**26**	**2**

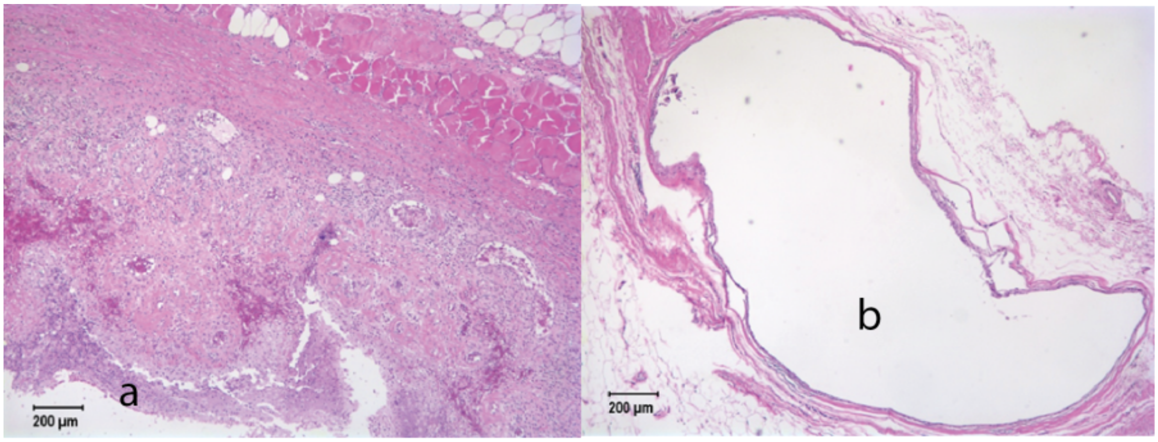

Figure S31. Rhesus macaque EE22. *In vitro* release rate 0.13 mg/day. Grossly there is an abscess containing serosanguinous watery fluid above the left scapular region. Histologically the left (active) implant (a) is surrounded by a thick capsule and thick layer of granulomatous inflammation with necrotic cellular material in the lumen. The right (placebo) implant (b) is surrounded by a thin capsule with minimal inflammation.

Table S14. Histological characteristic scores for rhesus macaque HC11. Implant placed 2/22/2018; euthanized 5/24/2018.

**Animal# HC11 Necropsy# 18A234**	**Response**	
Implant	Left (Active) (1-4)	Right (Placebo) (1-4)
Polymorphonuclear cells	4	1
Lymphocytes	4	2*
Plasma cells	4	1
Macrophages	4	0
Giant cells	3	0
Necrosis	3	0
Capsule thickness	3	2
Tissue infiltrate	3	1*
Other		
**Overall total**	**28**	**7=AV**

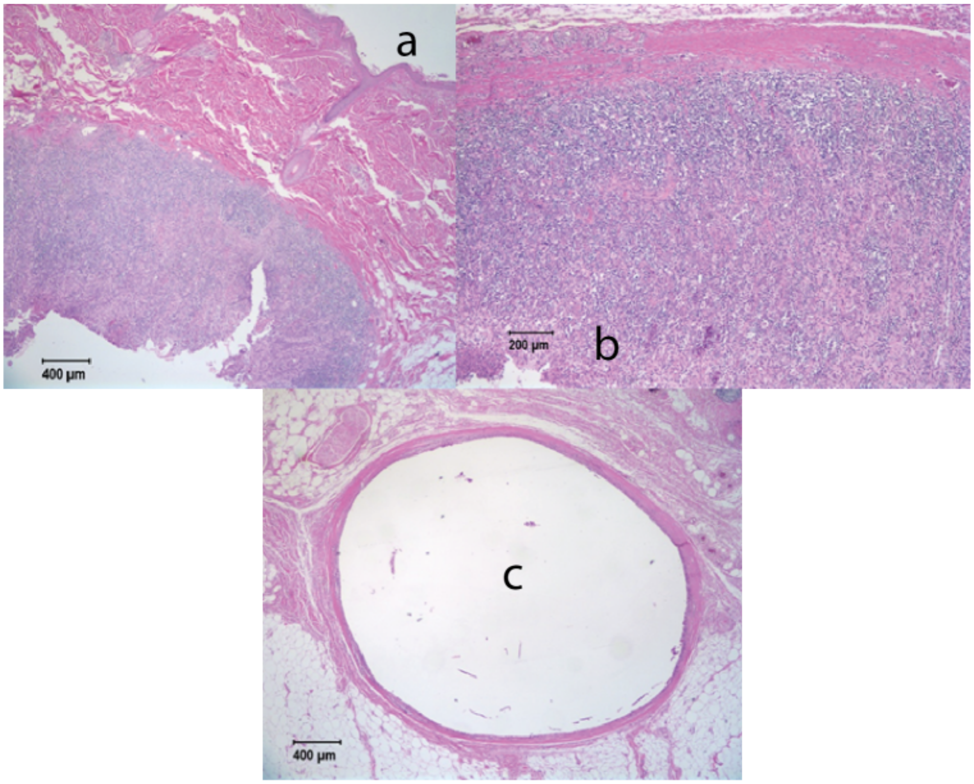

Figure S32. Rhesus macaque HC11. *In vitro* release rate 0.13 mg/day. Sections from left (active) implant (a,b) show thick fibrous capsule filled with heterophils, plasma cells, necrotic cellular debris, proteinaceous fluid, and occasional multinucleated giant cells. Little necrosis outside of capsule but multifocal aggregates of densely packed lymphocytes are in surrounding tissues. Right (placebo) implant (c) has minimal fibrosis or inflammation. A few heterophils and plasma cells in the lumen of the implant. Deeper tissues show mild infiltrate of lymphocytes and plasma cells but may be extensions from another implant.

Supplemental 6. Implant reactivity grade summary

Table S15. Implant reactivity scores and placebo-adjusted implant reactivity scores.

Animal	Implant Type	Time (week)	Number of implants tested	Average implant reactivity score (0-56)	Average placebo-adjusted implant reactivity score (S_{pair})[a]	Reactivity Grade
Rabbit	Placebo Gen A 0.8 cm	12	9	6		
Rabbit	Active Gen A 0.8 cm	12	8	37	31	Severe reaction
Rabbit	Active Gen A 1.6 cm	12	8	40	34	Severe reaction
Macaque	Placebo Gen B 2.0 cm	4	2	11		
Macaque	Active Gen B 2.0 cm	4	2	34	23	Severe reaction
Macaque	Placebo Gen B 2.0 cm	12	4	16		
Macaque	Active Gen B 2.0 cm	12	4	48	32	Severe reaction

a. $\bar{S}_{pair}$ from 0.0 up to 2.9 no reaction, $\bar{S}_{pair}$ from 3.0 up to 8.9 a slight reaction, $\bar{S}_{pair}$ from 9.0 up to 15.0 a moderate reaction, and $\bar{S}_{pair}$ >15.1 is assigned severe reaction grade as per the standard (2).

Supplemental 7. Molecular weight analysis of polymer wall material before and after in vivo exposure

Method: The core of the implants, recovered after the animal studies, was extracted for residual drug analysis. The tubing left over after the extraction procedure was processed to determine molecular weight. The tubing was dried in vacuum for 24 hours at room temperature. The dried tubing was dissolved in tetrahydrofuran (THF) and filtered through a 0.2 µm PTFE syringe filter. Gel permeation chromatography (GPC) was then used to measure the molecular weight of the tubing. A GPC system (Wyatt Technology, Santa Barbara, CA) consisting of a multi-angle light scattering detector (DAWN HELEOS-II 8 angle), and refractive index detector (Optilab T-rEX) was used. Software provided by the GPC instrument manufacturer (ASTRA software, Version 7.0) was used to process data. The GPC column used was a PLgel

Mixed-B GPC column (10 µm, 7.5 x 300 mm) in combination with PLgel guard column (10 µm, 7.5 x 50 mm). The flow rate of the mobile phase, THF, was set at 0.7 mL/min. The dn/dc value of EG85A was 0.0756 mL/g.

The measured weight average molecular weights in TAF implant controls and implants from macaques for 12 weeks were 98.3±4.0 kDa and 95.9±2.9 kDa, respectively (Table S15. The measured weight average molecular weights of the TAF implant controls and implants from macaques for 12 weeks were 97.0±0.7 kDa and 91.3±2.5 kDa, respectively (Table S16).

Table S16. The number and weight average molecular weights (M_n, M_w) and polydispersity index values (M_w/M_n) of tubing recovered from Generation A implants in NZW rabbits after 12 weeks

ID Number	M_n (kDa)	M_w (kDa)	Polydispersity (M_w/M_n)
TAF Implant Controls			
Control 1	51.5	98.4	1.911
Control 2	50.5	96.7	1.914
Control 3	48.4	92.4	1.910
Control 4	52.0	102.7	1.974
Control 5	53.3	101.1	1.897
Average (n=5)	51.2±1.8	98.3±4.0	1.921±0.030
TAF Implants in NZW Rabbits (12 weeks)			
Implant 1	52.7	97.9	1.857
Implant 2	48.7	91.2	1.875
Implant 3	50.0	93.8	1.875
Implant 4	50.6	95.2	1.880
Implant 5	49.1	92.0	1.874
Implant 6	50.2	92.4	1.839
Implant 7	49.6	93.6	1.886
Implant 8	51.4	95.2	1.853
Implant 9	52.0	95.9	1.842
Implant 10	50.4	98.2	1.949
Implant 11	49.3	95.0	1.926
Implant 12	52.5	100.0	1.904
Implant 13	52.0	98.1	1.888
Implant 14	52.9	100.8	1.904
Implant 15	51.0	93.5	1.834
Average (n=15)	50.8±1.4	95.5±2.9	1.879±0.033

Table S17. The number and weight average molecular weights (M_n, M_w) and polydispersity index values (M_w/M_n) of tubing recovered from Generation A implants in rhesus macaques after 12 weeks

ID Number	M_n (kDa)	M_w (kDa)	Polydispersity (M_w/Mn)
TAF Implant Controls			
Control 1	48.3	96.8	2.003
Control 2	47.2	97.0	2.054
Control 3	46.1	97.8	2.120
Control 4	47.8	96.2	2.015
Average (n=4)	47.4±0.9	97.0±0.7	2.048±0.053
TAF Implants in Macaques (12 weeks)			
Implant 1	44.7	92.4	2.069
Implant 2	45.9	89.5	1.948
Implant 3	41.9	92.3	2.203
Implant 4	40.4	89.0	2.202
Implant 5	45.5	91.0	2.002
Implant 6	42.7	88.6	2.074
Implant 7	46.1	91.5	1.986
Implant 8	45.2	90.1	1.992
Implant 9	48.5	96.9	1.998
Average (n=9)	44.5±2.5	91.3±2.5	2.053±0.094

Supplemental 8. TFV-DP tissue and TAF & TFV plasma levels in rhesus macaques with generation B implants

Table S18. Gen B rhesus macaque TFV-DP tissue levels. TFV-DP tissue levels for BLQ for TFV-DP was <5 fmol/sample.

**Animal ID**	**Time (weeks)**	**Specimen Type**	**Final [TFV-DP] (fmol/mg)**
EC74	12	Implant	26.8
FC48	12	Implant	1.06
EE22	13	Implant	9.13
HC11	13	Implant	BLQ
EJ87	0	Rectal	BLQ
GR10	0	Rectal	BLQ
EJ87	4	Rectal	BLQ
GR10	4	Rectal	BLQ
EC74	12	Rectal	BLQ
EC74	12	Rectal	BLQ
EE22	12	Rectal	BLQ

FC48	12	Rectal	11.8
FC48	12	Rectal	9.12
HC11	12	Rectal	BLQ
IT23	12	Rectal	2.18
JC07	12	Rectal	2.10
EE22	13	Rectal	BLQ
EE22	13	Rectal	BLQ
HC11	13	Rectal	1.21
HC11	13	Rectal	1.40
IT23	13	Rectal	1.80
JC07	13	Rectal	7.20
EJ87	0	Vagina	BLQ
GR10	0	Vagina	BLQ
EJ87	4	Vagina	BLQ
GR10	4	Vagina	BLQ
EC74	12	Vagina	BLQ
EC74	12	Vagina	BLQ
EE22	12	Vagina	BLQ
FC48	12	Vagina	BLQ
FC48	12	Vagina	0.994
EE22	13	Vagina	BLQ
EE22	13	Vagina	BLQ

Table S19. Gen B rhesus macaque TAF and TFV plasma levels.

Specimen ID	Time Point	Final [TAF] (ng/mL)	Final [TFV] (ng/mL)
DP15	Week 0	BLQ	BLQ
DP15	Week 1	0.050	0.358
DP15	Week 2	0.106	0.415
DP15	Week 3	0.111	0.487
DP15	Week 4	0.078	0.531
EC74	Week 0	BLQ	BLQ
EC74	Week 1	0.064	0.373
EC74	Week 10	BLQ	0.034
EC74	Week 12	BLQ	BLQ
EC74	Week 12	BLQ	BLQ
EC74	Week 2	BLQ	0.371
EC74	Week 3	0.035	0.621
EC74	Week 4	0.041	0.459

EC74	Week 5	0.803	0.07
EC74	Week 6	0.55	0.097
EC74	Week 7	0.674	0.059
EC74	Week 8	0.79	0.066
EC74	Week 9	BLQ	0.046
EE22	Week 0	BLQ	BLQ
EE22	Week 1	0.043	0.606
EE22	Week 10	BLQ	BLQ
EE22	Week 11	BLQ	BLQ
EE22	Week 12	BLQ	BLQ
EE22	Week 13	BLQ	BLQ
EE22	Week 13	BLQ	BLQ
EE22	Week 2	0.069	0.313
EE22	Week 3	BLQ	6.95
EE22	Week 4	0.048	0.696
EE22	Week 5	0.596	0.11
EE22	Week 6	0.529	0.109
EE22	Week 7	0.51	0.075
EE22	Week 8	0.624	BLQ
EE22	Week 9	BLQ	BLQ
FC48	Week 0	BLQ	BLQ
FC48	Week 1	0.040	1.05
FC48	Week 10	2.55	0.108
FC48	Week 12	0.819	0.124
FC48	Week 12	0.706	0.052
FC48	Week 2	0.053	0.941
FC48	Week 3	0.042	0.488
FC48	Week 4	0.062	0.560
FC48	Week 5	0.414	0.07
FC48	Week 6	0.56	0.115
FC48	Week 7	0.489	0.078
FC48	Week 8	0.726	0.101
FC48	Week 9	1.08	0.129
GH84	Week 0	BLQ	BLQ
GH84	Week 1	0.051	BLQ
GH84	Week 2	0.095	BLQ
GH84	Week 3	0.064	BLQ
GH84	Week 4	0.080	0.345
HC11	Week 0	BLQ	BLQ
HC11	Week 1	0.044	BLQ

HC11	Week 10	BLQ	BLQ
HC11	Week 11	0.663	BLQ
HC11	Week 12	BLQ	BLQ
HC11	Week 13	BLQ	BLQ
HC11	Week 13	BLQ	BLQ
HC11	Week 2	BLQ	BLQ
HC11	Week 3	BLQ	BLQ
HC11	Week 4	BLQ	0.578
HC11	Week 5	BLQ	BLQ
HC11	Week 6	BLQ	BLQ
HC11	Week 7	0.343	BLQ
HC11	Week 8	0.512	BLQ
HC11	Week 9	0.361	BLQ
IT23	Week 0	BLQ	BLQ
IT23	Week 1	0.045	BLQ
IT23	Week 10	0.491	0.045
IT23	Week 11	BLQ	0.038
IT23	Week 12	0.701	0.063
IT23	Week 13	0.73	0.048
IT23	Week 14	1.01	0.117
IT23	Week 2	0.031	BLQ
IT23	Week 3	BLQ	0.519
IT23	Week 4	0.038	0.485
IT23	Week 5	0.339	BLQ
IT23	Week 6	0.392	0.058
IT23	Week 7	0.437	0.054
IT23	Week 8	0.629	0.051
IT23	Week 9	3.66	0.038
JC07	Week 0	BLQ	BLQ
JC07	Week 1	BLQ	BLQ
JC07	Week 10	0.597	0.09
JC07	Week 11	0.444	0.059
JC07	Week 12	BLQ	0.057
JC07	Week 13	BLQ	0.074
JC07	Week 14	0.624	0.052
JC07	Week 2	BLQ	BLQ
JC07	Week 3	BLQ	BLQ
JC07	Week 4	BLQ	0.341
JC07	Week 5	0.481	BLQ
JC07	Week 6	BLQ	BLQ

JC07	Week 7	0.552	BLQ
JC07	Week 8	0.445	BLQ
JC07	Week 9	0.456	0.069

Supplemental 9. Use of a trocar for implantation in a PK and safety study of the generation B implant in rhesus macaques

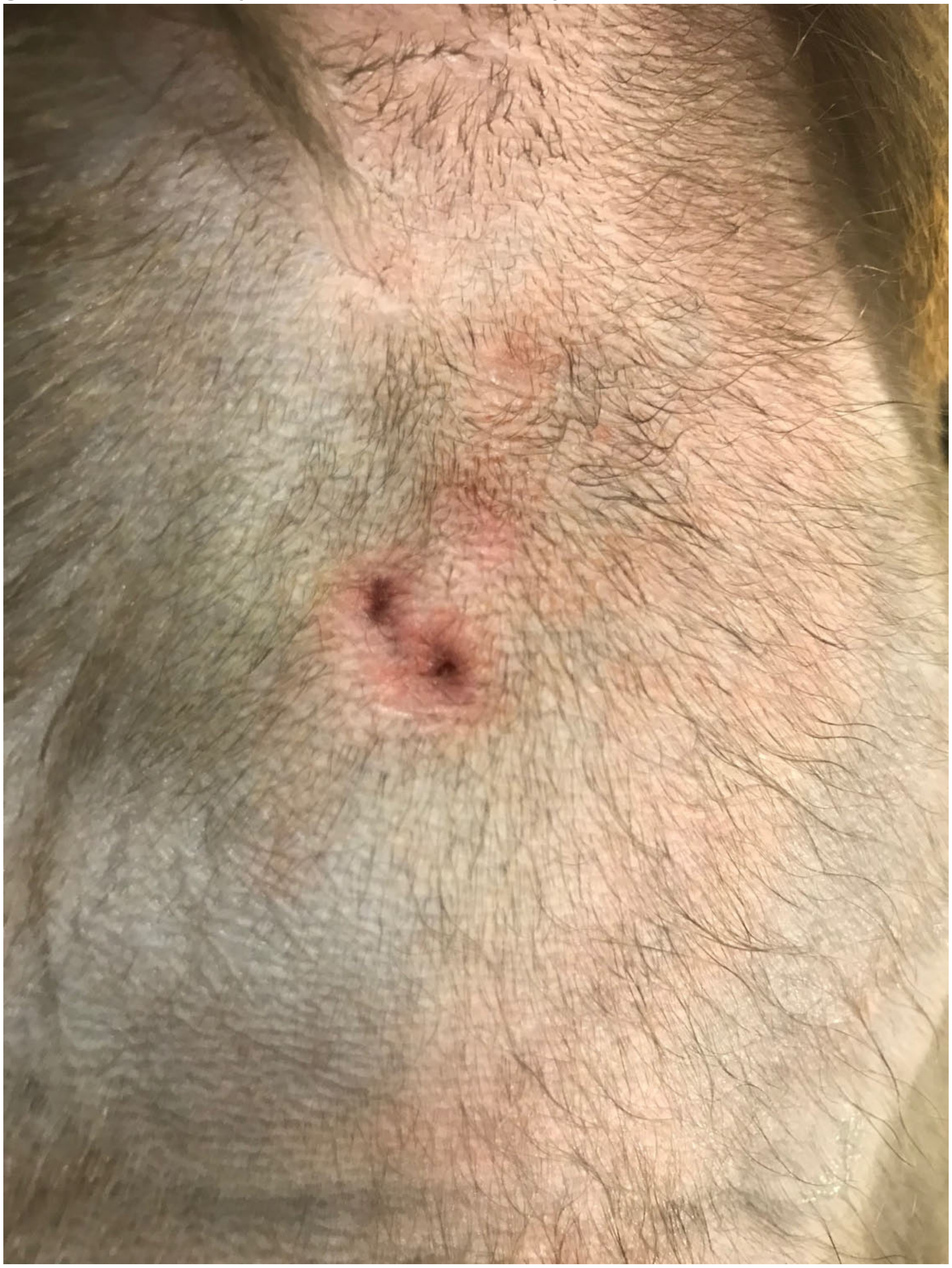

Figure S33. Trocar inserted implant resulted in a puncture like lesion. IT19 had 2 puncture-like lesions (with a small amount of bloody purulent discharge in the area of the left implant. The animal was started on antibiotics.

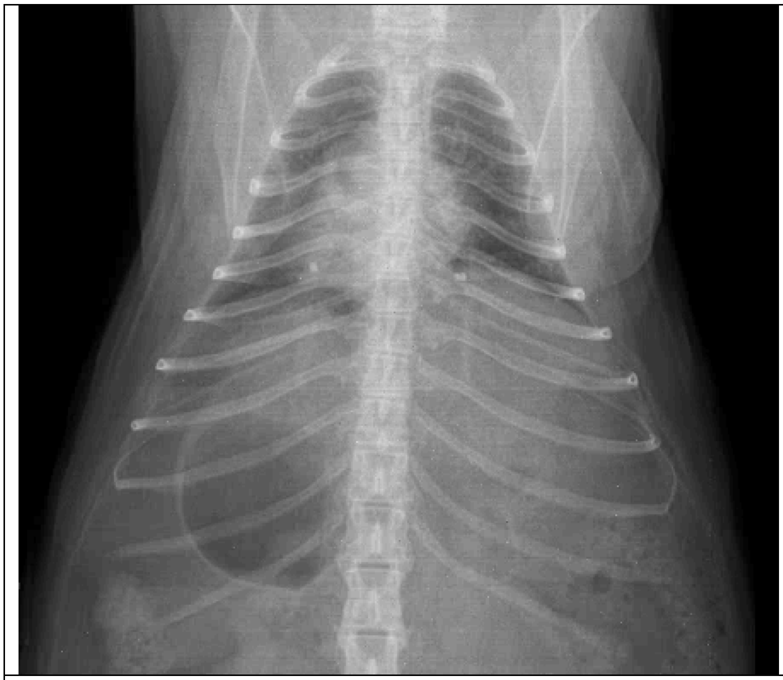

Figure S34. X-ray image of rhesus macaque with trocar inserted implants. An x-ray opaque barium sulfate pellet was added to each implant.

References

1. Clark JT, Clark MR, Shelke NB, Johnson TJ, Smith EM, Andreasen AK, Nebeker JS, Fabian J, Friend DR, Kiser PF. 2014. Engineering a segmented dual-reservoir polyurethane intravaginal ring for simultaneous prevention of HIV transmission and unwanted pregnancy. PLoS One 9:e88509.

2. International Organization for Standardization. 2016. Biological Evaluation of Medical Devices 3rd Edition. . International Organization for Standardization.
